# *Bacillus* spore probiotics for alleviating functional constipation in children: a randomized, double-blind, placebo-controlled trial

**DOI:** 10.1038/s43856-026-01517-6

**Published:** 2026-03-18

**Authors:** Hanh Thi Luong Nguyen, Hang Thi Hoang, Dung Phuong Le, Truong Quoc Duong, Ngoc Thi Ho Vuong, Mai Tuyet Truong, Hung Trong Nguyen, Anh Hoa Nguyen, Tung Dinh Pham, Anh Thi Van Nguyen

**Affiliations:** 1 National Institute of Nutrition, Hanoi, Viet Nam; 2 Spobiotic Research Center, ANABIO R&D Ltd. Company, Hanoi, Viet Nam; 3 Thai Nguyen University of Medicine and Pharmacy, Thai Nguyen, Viet Nam; 4 LiveSpo Pharma Ltd. Company, Hanoi, Viet Nam; 5 Faculty of Mathematics - Mechanics - Informatics, VNU University of Science, Vietnam National University, Hanoi, Viet Nam

**Keywords:** Constipation, Randomized controlled trials, Paediatric research, Microbiome

## Abstract

**Background:**

Functional constipation is common in children and often responds poorly to standard treatments. This study evaluated the efficacy and mechanisms of multi-strain *Bacillus* spore probiotics, which tolerate gastrointestinal conditions, in paediatric functional constipation.

**Methods:**

We conducted a randomized, double-blind, placebo-controlled trial (ClinicalTrials.gov NCT06154525, 4/12/2023) in preschool children (24–60 months) with functional constipation in Vietnam. A total of 111 participants were randomly assigned (1:1:1) to receive placebo or two multi-strain *Bacillus* spore probiotics (LiveSpo Kids or Preg-Mom, ≥3 billion CFU/5 mL registered; each tested at 3.7 billion CFU/5 mL) for 28 days. Primary outcomes were changes in functional constipation (main focus), anorexia, and underweight risk at day 28. Secondary outcomes included serum cytokines, stool IgA, and gut microbiota; stool samples from 10 healthy children provided a reference microbiota profile.

**Results:**

Both probiotic groups show significant improvements at day 28. Percentages of children with constipation decrease 3.7-fold in Kids and 5.1-fold in PregMom (*p* < 0.0001). Absolute Risk Reductions (ARR) are 52.38% (95%CI: 35.45%-77.26%) and 59.97% (95%CI: 44.48%-84.68%) in Kids and PregMom vs Placebo. Kids and PregMom groups improve anorexia (ARR: 24.40% (95%CI: 3.09%-49.44%) and 25.98% (95%CI: 4.69%-51.25%)) and underweight risk (ARR: 7.87% (95%CI: 0%-23.80%) and 19.30% (95%CI: 1.70%-37.50%)) vs. Placebo. Probiotics reduce serum IL-6 and IL-23, increase IL-10 and stool IgA, and shift the gut microbiota toward a composition more closely resembling healthy children, enriching beneficial species while reducing harmful ones.

**Conclusions:**

Multi-strain *Bacillus* spore probiotics alleviate functional constipation, improve immune markers, and modulate gut microbiota in children, supporting their potential as effective microbiome-targeted interventions.

## Plain language summary

Constipation is common in young children, leading to infrequent and painful bowel movements. Probiotics are live microorganisms that can provide health benefits when consumed, primarily by improving the composition of microorganisms within the gut. *Bacillus* spore probiotics can survive harsh gastrointestinal conditions, allowing them to reach the gut and act effectively. We tested two multi-strain *Bacillus* spore probiotics, LiveSpo Kids and Preg-Mom, in children aged 24 to 60 months with constipation. After drinking these probiotics twice daily for four weeks, children had significantly better bowel movements than those given water. They also showed better appetite and healthier weight gain. We propose these probiotics may be a safe and effective approach to treat childhood constipation, especially in areas with limited access to specialized medical care.

## Abstract (web summary)

Nguyen et al. conduct a randomized, double-blind, controlled clinical trial evaluating multi-strain Bacillus spore probiotics for treating functional constipation in preschool children. The probiotics significantly reduce constipation, anorexia, and underweight risk and improve immune markers and gut microbiota composition after 7–28 days of use.

## Introduction

Functional constipation, defined by the Rome IV criteria, accounts for ~95% of constipation cases, affecting roughly 14.4% of children worldwide^1,2^. Characterized by infrequent or difficult bowel movements with abdominal discomfort, it can impair growth and lead to behavioral distress if untreated. Conventional treatments, including diet, behavioral therapy, and laxatives, often provide only temporary or inconsistent relief^2^. Recent studies implicate gut microbiota’s dysbiosis in constipation, highlighting its role in intestinal health, immunity, and metabolism^3–5^. Probiotics may help restore microbial balance by modulating the microbiota, strengthening the intestinal barrier, and regulating immune signaling^6^ via interacting with the gut-associated lymphoid tissue^7^, influencing the production of cytokines and IgA production crucial for immune regulation and pathogen defense^8,9^.

Although probiotics are widely recognized for their broad health benefits, their efficacy in paediatric functional constipation remains under-investigated. Clinical trials using *Lactobacillus* and *Bifidobacterium* strains have shown gradual symptom improvement after 4–12 weeks of supplementation^10,11^. However, systematic reviews point out major limitations, including low methodological quality, risk of bias, inconsistent outcomes, and lack of strain standardization, which hinder comparisons^12,13^. The slow and variable efficacy of these traditional bacterial strains may stem from their sensitivity to heat, acidity, and bile, reducing their survival and intestinal delivery^10,12–15^. In contrast, *Bacillus* spore probiotics have attracted increasing attention for their safety, stability under extreme conditions, and gut health benefits^16,17^. After surviving gastric transit, *Bacillus* spores germinate in the ileum and colon, where their vegetative cells transiently colonise^18,19^ and secrete bacteriocin-like peptides with broad antimicrobial activity^20,21^. Among these, *Bacillus subtilis* CU1 was shown to stimulate human immune responses in cytokine and antibody production^22^. *B. subtilis* DE111 secretes diverse digestive enzymes that may support carbohydrate, protein, and lipid degradation^23^. *Bacillus clausii* (O/C, SIN, N/R, T) demonstrates immunomodulatory effects supporting intestinal homeostasis^24^, while *Bacillus coagulans* T242, CGI314 and Unique IS2 strains generate lactic acid and enhance beneficial microbiota, with evidence from both in vitro and clinical IBS studies^25–29^. Clinical studies on *B. clausii* and *B. coagulans* have reported constipation symptom relief, though significant effects were typically revealed only after 4–8 weeks^28–30^. Notably, these studies have not assessed immune markers and gut microbiota, leaving key mechanisms of probiotic unclear.

This study evaluated the safety and efficacy of two liquid multi-strain *Bacillus* spore formulations, LiveSpo Kids (*B. clausii* ANA39, *B. subtilis* ANA3) and LiveSpo Preg-Mom (*B. clausii* ANA39, *B. subtilis* ANA46, *B. coagulans* ANA40), in preschool children with functional constipation over 28 days. Primary outcomes included changes in the percentage of children meeting Rome IV criteria for constipation (the main focus), anorexia (National Institute of Nutrition criteria), and risk of underweight (WHO criteria). Secondary outcomes assessed serum cytokines (IL-6, IL-17, IL-23, TNF-α, IL-10), fecal IgA, and gut microbiota composition. We hypothesised that both formulations would rapidly relieve constipation and modulate immune and microbiota profiles. The findings confirm our hypotheses, showing that both probiotic formulations markedly reduce constipation, anorexia, and underweight risk in children, with substantial absolute risk reductions (ARRs) compared with placebo. They also improve inflammatory and immune markers and enrich beneficial gut bacteria. This probiotic approach represents a promising option for functional constipation, especially where specialist medical resources are limited.

## Methods

### Study design and ethics

**Fig. 1:**
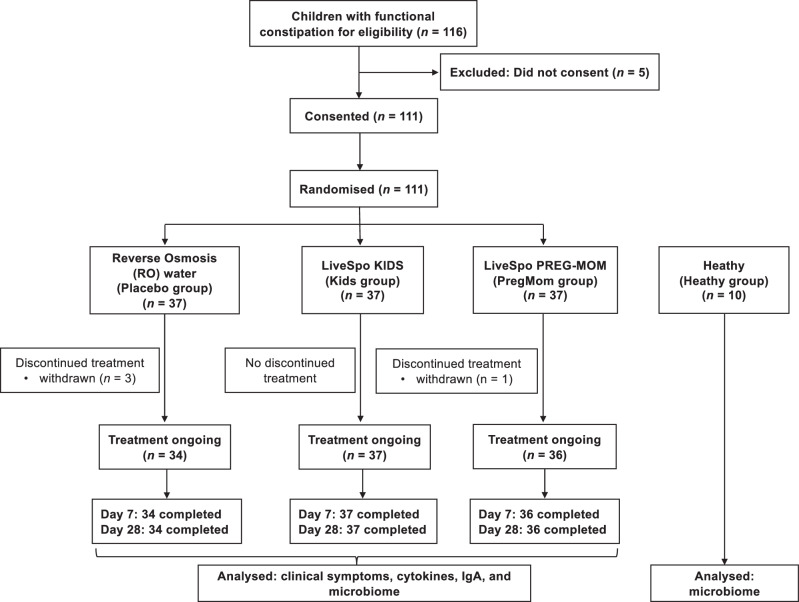
CONSORT diagram. CONSORT flow diagram of participant recruitment, allocation, follow-up, and analysis in the NCT06154525 clinical trial.

This community-based, multi-center, randomized, double-blind, controlled trial was conducted in three preschools (Lau Thuong, Trang Xa and Dan Tien), at Vo Nhai County, Thai Nguyen Province, Vietnam, from December 4, 2023, to July 30, 2024. The study was approved by the Ethics Committee of the Vietnam National Institute of Nutrition (Decision 1224/QLKH-VDD, November 14, 2023) and followed the principles of the Declaration of Helsinki and ICH-GCP guidelines. Written informed consent was obtained from all participants’ parents or guardians before enrollment. Participants could withdraw at any time and deny the use of their previously collected data, including baseline assessments, in analyses or reports. The trial was registered at ClinicalTrials.gov (NCT06154525, 4/12/2023). The flowchart of the study design is presented in Fig. 1.

### Sample size and participant recruitment

The sample size calculation was based on an expected 35% improvement in functional constipation symptoms with probiotic use, assuming that 75% of children in the placebo group and 40% in the probiotic group would remain symptomatic. With a significance level (*α*) of 0.05 and 80% power, the estimated required sample size was 30 participants per group^31^. To account for an anticipated 25% dropout rate, 37 children were enrolled in each group.

Participant enrollment, screening, and health assessments were conducted on-site at the three preschools between February and March 2024, enabling rapid recruitment and efficient 28-day follow-up, which was completed by April 2024. The remaining time was dedicated to preparation, personnel training, wet-lab experiments, data analysis, and reporting. All children at these preschools were screened by trained study physicians using a structured questionnaire based on Rome IV G7 criteria for pediatric functional constipation^32^. Children diagnosed with functional constipation were recruited into the interventional trial. At baseline, enrolled children were also assessed for anorexia and underweight risk through structured caregiver interviews and anthropometric measurements.

Inclusion criteria were: (1) aged 24–60 months with diagnosed functional constipation; (2) enterally fed and weaned from breastfeeding; and (3) guardians understood and agreed to the study.

Exclusion criteria included: (1) age outside 24–60 months or not residing in the study areas; (2) acute infections, liver/kidney diseases, preterm birth, low birth weight (<2500 g), chronic illnesses, or congenital abnormalities (e.g., Crohn’s disease, anorectal stenosis, megacolon, hypothyroidism, celiac disease); (3) use of medications that may affect gastrointestinal motility or the gut microbiota (e.g. laxatives or probiotics) before or during the study; and (4) guardians declined consent or failed to comply.

In parallel, at the same preschools, children who met similar demographic characteristics but did not meet the Rome IV G7 criteria for constipation, and who were free from anorexia, risk of underweight, or any gastrointestinal complaints, were recruited as a healthy reference group. These children did not participate in the intervention but provided stool samples for gut microbiota analysis as healthy controls.

### Randomization and blinding

A total of 111 preschool children aged 24–60 months with functional constipation were randomized in a 1:1:1 ratio to receive LiveSpo Preg-Mom, LiveSpo Kids, or placebo (reverse-osmosis water), using permuted blocks of three. Anorexia and risk of underweight were not used as stratification factors in this randomization. The randomization sequence was generated by an independent statistician. Each treatment group was assigned a non-identifiable product code (A, B, or C), with the securely stored code-to-group mapping known only to the statistician.

Identical opaque plastic ampoules were pre-labeled by the manufacturer with these product codes solely for blinding purposes and independent of the allocation sequence. Each participant was assigned a unique study ID. Allocation concealment was ensured using opaque, sealed, sequentially numbered envelopes (SNOSE) prepared from the randomization list. An independent staff member, not involved in recruitment or outcome assessment, opened the next envelope in sequence after enrollment and dispensed the correspondingly coded ampoule.

The allocation list linking product codes to treatment groups remained concealed from investigators, healthcare staff, preschool teachers, participants, and their guardians throughout the study. Access to the code was strictly limited to cases of emergency unblinding due to serious adverse events or after database lock for final analysis. Blinding was therefore fully maintained for all personnel and participants across the trial.

### Products and interventions

LiveSpo Preg-Mom, manufactured by LiveSpo Pharma in Hanoi, Vietnam, is a reverse osmotic (RO) water suspension containing *B. clausii* ANA39, *B. subtilis* ANA46, and *B. coagulans* ANA40 spores. For the batch used in this clinical trial, the verified viable count was 3.7 billion CFU per 5 mL ampoule. The product is labeled to contain ≥3 billion CFU/5 mL and is registered under number 7695/2020/DKSP with the Food Safety Department of the Ministry of Health, Vietnam. Similarly, LiveSpo KIDS contains *B. clausii* ANA39 and *B. subtilis* ANA3 spores. The batch used in this study also had a verified viable count of 3.7 billion CFU/5 mL, while the labeled specification is ≥3 billion CFU/5 mL, registered under number 1537/2023/DKSP. All products met microbiological and heavy metal safety standards before use in the trial.

Before the manufacture and clinical study, the strain underwent microbial and biochemical characterization, antibiotic susceptibility testing, 16S rRNA sequencing, and whole genome analysis. Summarized data on (i) microbial and biochemical characterization, (ii) antibiotic susceptibility, (iii) 16S rRNA sequencing analysis, and (iv) sequence analyses of antibiotic resistance and toxin genes in the whole genomes of *B. subtilis* ANA3, *B. subtilis* ANA46, *B. clausii* ANA39, and *B. coagulans* ANA40 are presented in the Supplementary Information (Supplementary Figs. 1–5 and Supplementary Tables 1–7). These results indicate that both strains are safe in *in-vitro* tests, demonstrate high spore formulation efficiency (>90%), are heat-stable (>65 °C), and can survive under both aerobic and anaerobic conditions. Among the four probiotic strains, only *B. subtilis* ANA3 carried two resistance-related genes, *aadK* (aminoglycoside) and *mph(K)* (macrolide). No mobile or transferable elements were found near these loci, confirming that both genes are chromosomally encoded and non-transferable. No other resistance genes were detected, indicating that the observed trimethoprim resistance is intrinsic. Acute and sub-acute toxicity studies of LiveSpo Kids and LiveSpo Preg-Mom conducted in mice at 250-fold human dose and rabbits at a threefold human dose by the Vietnam National Institute for Drug Quality Control demonstrated that both products are non-toxic (Supplementary Data 1 and 2).

The placebo (RO water) was packaged in identical opaque white ampoules designed for clinical use, ensuring complete visual masking from the probiotic ampoules. All ampoules were administered by direct drinking, making the solution’s turbidity invisible. Neither participants nor investigators could distinguish between placebo and probiotic products by appearance, taste, or smell. Probiotics and a placebo (RO water only) were given as two ampoules daily for 28 days. The administration was supervised at preschools during school hours; on weekends, the product was delivered to participants’ homes with instructions for continued use. Daily intake was documented on dose logs completed by teachers during school days and by parents at weekends, and logs were reviewed weekly by the study team to ensure adherence. Additionally, children in all three groups received standardized counseling on nutrition and the dietary management of constipation, following guidelines of the National Institute of Nutrition. Parents or caregivers were advised to encourage their children to follow dietary instructions, including increasing fiber intake, stay hydrated (1–1.5 L/day), limiting constipating foods, and maintaining healthy bowel habits throughout the 28-day study. No additional pharmacological treatment for constipation was administered during the study.

### Safety and primary outcome assessments

Predefined adverse events, including abdominal pain, nausea, vomiting, fever, rash, itching, diarrhea, and stool color change, were recorded daily on checklists by teachers or parents. Reports were reviewed daily by study physicians, who graded severity (mild, moderate, severe), assessed causality, ensured all events were followed to resolution, and any serious event was promptly reported to the ethics board for potential emergency unblinding.

Primary outcomes were captured at clinic visits via investigator-administered structured questionnaires (days 0, 7, 28) developed from Rome IV G7 criteria^32^, national nutrition guidance and underweight risk (WHO criteria)^33^; these assessment tools were ethics-approved and supervisor-verified before database entry. Parents also kept a daily diary documenting stool frequency and form (Bristol scale), soiling, pain, withholding or straining, diarrhea or undigested stool, intercurrent symptoms, and study-product intake or adverse reactions. The diaries were monitored, reminded, and reviewed weekly by supervisors and reconciled during study visits. Diary records supported recall but were not included in the primary analysis dataset.

Functional constipation was diagnosed if at least two of the following symptoms persisted for one month in children under 48 months or two months in children over 48 months: (i) ≤2 bowel movements per week, (ii) withholding behavior, (iii) hard stools or anal pain, (iv) large-diameter stools. In addition, straining during defecation and stool retention posture were also recorded as constipation-related symptoms. Improvement was defined as having fewer than two ROME IV G7 symptoms or complete symptom resolution. All constipation-related symptoms were assessed through structured interviews with parents using a pre-designed questionnaire to determine the presence or absence of each symptom. Stool consistency was evaluated using the Bristol Stool Form Scale, with parents identifying their child’s stool type from standardized images^34^.

For anorexia assessment at baseline, as no universally accepted guideline exists for pediatric anorexia in otherwise healthy children, apart from definitions for specific eating disorders such as anorexia nervosa, bulimia nervosa, or binge eating disorder^35^, we applied criteria based on the guideline of the National Institute of Nutrition, Vietnam. Children were diagnosed with anorexia if they exhibited any of the following symptoms in the past month: (i) food refusal, selective eating, prolonged food retention in the mouth; (ii) consuming less than half of age-recommended dietary intake; or (iii) consuming more than half a main meal but only under pressure and with prolonged eating duration (>30 min). This was evaluated through a structured questionnaire asking parents about their child’s eating behavior, including the presence of anorexia (yes/no), average duration of main meals (≤30 min, 30–60 min, or >60 min), and eating attitudes (multiple-choice: eating voluntarily, holding food in mouth, eating slowly but finishing meals, requiring force or threats, leaving >50% of meals, leaving <50% of meals). At follow-up visits (days 7 and 28), the assessment included changes in eating behavior since intervention initiation (improve progress, eat faster, eat with a better appetite, decrease appetite, or no change), food refusal (yes/no), food retention in the mouth (same as before, improved, or none), meal duration (≤30 min, 30–60 min, or >60 min), number of main meals and snacks per day, and leftover food behavior (same as before, improved, or none).

For evaluation of underweight and risk of underweight, children’s weight was measured using a calibrated electronic scale at days 0, 7, and 28. Weight-for-Age Z-scores (WAZ) were calculated based on the WHO Child Growth Standards for children under 60 months of age. Underweight was defined as WAZ <–2 SD according to WHO standards, while children with WAZ between –2 SD and –1 SD were classified as at risk of underweight, consistent with WHO growth chart interpretation guidelines.

For the three primary outcomes (constipation, anorexia, and underweight), ARR was calculated using the formula: ARR = % of patients in the Control group - % of patients in the probiotic intervention group. NNT (Number Needed to Treat) was the reciprocal of the ARR, indicating the number of patients need treatment to prevent one case of the condition.

### Secondary outcomes and *Bacillus* assessments

Secondary outcomes included: (1) changes in immune markers, with blood cytokine levels (IL-6, IL-17, IL-23, TNF-α, IL-10) assessed at day 28 versus day 0, and stool IgA levels at days 7 and 28 versus day 0; (2) stool microbiota composition at day 28 versus day 0.

Blood samples were collected at two time points (days 0 and 28), while stool samples were collected at three time points (days 0, 7, and 28). For stool sample collection, parents or guardians obtained approximately 5 g from the mid-portion of each child’s first morning stool into pre-labeled sterile screw-cap tubes without buffer. All samples were kept at 4 °C onsite and transferred on ice within three hours to prevent DNA and protein degradation. Blood samples were centrifuged to separate plasma for measuring IL-6, IL-17, IL-23, TNF-α, and IL-10. Stool samples were aliquoted into 200 mg portions for assays including stool IgA ELISA, microbiota analysis, and *Bacillus* detection. All samples were stored at −80 °C until analysis.

Pro- and anti-inflammatory cytokines (IL-6, IL-17, IL-23, TNF-α, IL-10) in all blood samples were quantified using ELISA kits (R&D Systems, MN, US)^36^. Similarly, IgA levels in all stool samples were measured at the same time points using ELISA kits (Thermo Fisher Scientific, US).

For microbiota analysis, a total of 70 representative stool samples were selected from four groups: healthy children (*n* = 10), and children with constipation in the Placebo, Kids, and PregMom groups (*n* = 10/group/time point at days 0 and 28). Stool samples from the three constipation groups (coded A, B, and C) were randomly selected using a stratified approach based on sex, nutritional status, recent antibiotic use, and defecation frequency to ensure baseline clinical diversity. Only stool samples from children without recent antibiotic exposure were included. Group codes were unblinded only after analysis completion. Stratification considered gender (male/female), nutritional status (malnourished vs. non-malnourished), recent antibiotic use (within 2 weeks), and defecation frequency (≤2 bowel movements/week). For the Healthy group, 10 samples were randomly selected to match age and gender distribution with the intervention groups. All samples were analyzed for changes in microbiota composition, including alpha and beta diversity and taxonomic classification at the phylum, family, genus, and species levels, using 16S rRNA sequencing (V3–V4 region) on the Illumina MiSeq platform (Macrogen, South Korea). Sequencing data were processed using CD-HIT-out-Miseq and rDnaTools, and analyzed with Qiime2 v2021.4 and the R microbiome v1.14.0 package.

Additionally, *B. subtilis, B. clausii*, and *B. coagulans* were detected in all stool samples on days 0, 7, and 28 using the real-time polymerase chain reaction (PCR) TaqMan probe method to monitor compliance with product or placebo use. Purified DNA from stool samples was used as the template for the real-time PCR assay. Primer and probe sequences designed for the assay are listed in Supplementary Table 8. Protocols were optimized following ISO 17025:2017 guidelines and performed on a 7500 Fast Dx qPCR system (Applied Biosystems™, Massachusetts, USA). Thermal cycling conditions were as follows: 95 °C for 15 min, then 45 cycles at 95 °C for 10 s and 60 °C for 30 s. For standardization, *C*_*t*_ values were set at <35.

### Statistics and reproducibility

As participants used their rights to withdraw all previously collected data, including baseline assessments, no baseline data were retained for excluded participants. As a result, intention-to-treat (ITT) analysis was not feasible, and only per-protocol (PP) analysis was conducted. Statistical analyses were performed using GraphPad Prism v10.0.0 and SPSS Statistics 29.0.0. Continuous variables were compared using *t*-tests or Mann–Whitney U tests, while categorical variables were analyzed using chi-square or Fisher’s exact tests. Longitudinal data were assessed using repeated measures analysis of variance (ANOVA) or linear mixed-effects models. Microbiome diversity and taxonomic composition were evaluated using Qiime2 and R microbiome tools. For within-group analyses of the three prespecified primary outcomes (constipation, anorexia, and risk of underweight), the Bonferroni-adjusted two-sided significance level was set at *p* < 0.0167 (0.05/3) to account for multiple comparisons. For between-group comparisons (PregMom and Kids vs. placebo), to account for multiplicity across the three prespecified primary outcomes, a Bonferroni-adjusted two-sided significance threshold of *p* < 0.0083 (0.05/6) was applied. Adjusted *p*-values are reported for these endpoints, with 95% confidence intervals provided for effect-size interpretation. Specific *p*-values and additional test details are provided in the figure legends.

Primary outcomes (functional constipation, anorexia, and underweight) were analyzed using *n* = 34 (Placebo), *n* = 37 (Kids), and *n* = 36 (PregMom). For cytokines and stool IgA, the number of measurable samples was equal to or lower than the number of enrolled participants because insufficient sample volume remained after failed and repeated ELISA or real-time PCR runs. The resulting analyzable sample sizes for Placebo, Kids, and PregMom were: 33, 35, and 36 for IL-6; 34, 37, and 34 for IL-17; 34, 35, and 34 for IL-23; 32, 36, and 34 for TNF-α; 34, 37, and 36 for IL-10; and 34, 37, and 36 for stool IgA. Gut microbiota profiling was performed on 10 biologically independent samples per group. No technical replicates were generated, and each sample represents an independent participant.

## Results

### Participant flow and baseline characteristics

Among the 116 children screened for eligibility, 111 consented and were randomized into three groups (*n* = 37/group): Placebo (receiving RO water), Kids (LiveSpo Kids probiotics), and PregMom (LiveSpo Preg-Mom probiotics). Participant recruitment took place between February 2024 and March 2024, with follow-up completed by April 2024. During the 28-day follow-up, 3 participants from the Placebo group and 1 from the PregMom group withdrew within 1–2 days after enrollment, as their parents or guardians changed their decision due to family objections. As a result, the final analyses included 34, 37, and 36 participants in the Placebo, Kids, and PregMom groups, respectively (Fig. 1). Withdrawal rates did not differ significantly between Placebo vs Kids (*p* = 0.2397) and Placebo vs PregMom (*p* = 0.6145). Because these early withdrawals requested deletion of their records, leaving no baseline data, all analyses were conducted on the per-protocol (PP) cohort, and baseline characteristics as well as outcome results pertain solely to this population.

Baseline demographic and clinical characteristics were generally comparable across groups (Table 1). Minor baseline imbalances were likely due to chance, given the modest per-arm sample size (1:1:1) and absence of stratification at randomization. Participants were randomized by an independent statistician using permuted blocks of three, with allocation concealed through sequentially numbered opaque sealed envelopes. Group identities remained blinded until database lock. Key demographic variables included age and gender distribution. Clinical characteristics assessed at baseline comprised health status indicators such as anorexia, acute respiratory infections, WAZ, recent use of opioids, digestive enzymes, probiotics, antibiotics, and fibre supplements. Constipation-related symptoms included bowel movement frequency, stool consistency, withholding behavior, hard stools or anal pain, large diameter stools, straining during defecation, and stool retention posture. Throughout the study, adherence to the assigned interventions was closely monitored.

**Table 1:** Baseline demographic and clinical characteristics of participants included in the per-protocol analysis

Characteristics		Placebo group (*n* = 34)	Kids group (*n* = 37)	PregMom group (*n* = 36)
Age (months) (median) (95% CI)		50.00 (45.03–50.32)	44.00 (41.44– 46.99)	43.00 (39.72– 46.00)
Gender				
Male *n* (%)		19 (55.88)	16 (43.24)	17 (47.22)
Female *n* (%)		15 (44.12)	21 (56.76)	19 (52.78)
The health status of children				
Functional constipation *n* (%)		34 (100.00)	37 (100.00)	36 (100.00)
Anorexia *n* (%)		23 (67.65)	28 (75.68)	27 (75.00)
Acute respiratory infections *n* (%)		7 (20.59)	10 (27.03)	7 (19.44)
Weight for age Z-score (WAZ)				
WAZ ≤ -1 SD *n* (%) (Underweight or at risk of underweight)		34 (100.00)	37 (100.00)	36 (100.00)
WAZ < -2 SD *n (%)* (Underweight)		13 (38.24)	11 (29.73)	11 (30.56)
The status of use: (2 weeks before treatment)				
Opioid *n* (%)		0 (0)	0 (0)	0 (0)
Digestive enzymes, probiotic *n* (%)		8 (23.53)	2 (5.41)	5 (13.89)
Antibiotic *n* (%)		6 (17.65)	9 (24.32)	5 (13.89)
Fiber (FOS, GOS) or drink enough water *n* (%)		3 (8.82)	3 (8.11)	7 (19.44)
Functional constipation symptoms				
Number of bowel movements ≤2 times/week *n* (%)		15 (44.12)	18 (48.65)	16 (44.44)
Stool consistency	Type 1 *n* (%)	2 (5.88)	5 (13.51)	4 (11.11)
	Type 2 *n* (%)	13 (38.24)	10 (27.03)	12 (33.33)
	Type 3 *n* (%)	19 (55.88)	22 (59.46)	20 (55.56)
Withholding defecation *n* (%)		19 (55.88)	18 (48.65)	15 (41.67)
Hard stools/anal pain *n* (%)		32 (94.12)	36 (97.30)	35 (97.22)
Large diameter stools *n* (%)		26 (76.47)	29 (78.38)	28 (77.78)
Straining during defecation *n* (%)		20 (58.82)	22 (59.46)	23 (63.89)
Posture for stool retention *n* (%)		19 (55.88)	21 (56.76)	18 (50.00)

As part of the compliance assessment, real-time PCR at day 0 showed no detectable bacilli in any group. By days 7 and 28, *B. subtilis* and *B. clausii* were detected in all samples of the Kids group, while *B. subtilis*, *B. clausii*, and *B. coagulans* were present in all samples of the PregMom group. No bacilli were detected in the Placebo group (Supplementary Figs. 6 and 7).

### Safety and clinical outcome improvement with *Bacillus* probiotics

During the 28-day follow-up period, no adverse reactions were reported in the two groups using LiveSpo Kids and Preg-Mom products. This includes the absence of diarrhea, vomiting, digestive issues, temporary gas or bloating, increased thirst, fever, or any signs of allergic reactions.

**Fig. 2:**
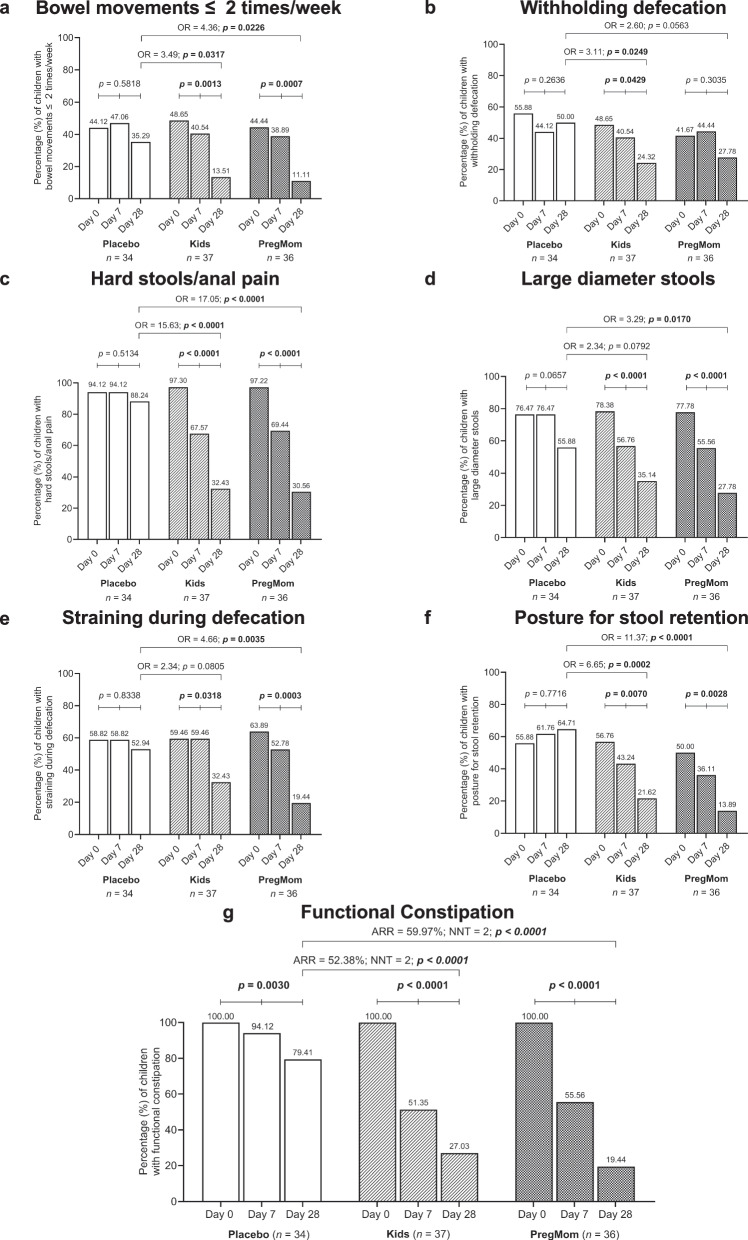
Effects of probiotic interventions on the proportion of children presenting individual constipation symptoms and functional constipation. Individual constipation symptoms include: **a** bowel movements ≤2 times/week; **b** withholding defecation; **c** hard stools/anal pain; **d** large-diameter stools; **e** straining during defecation; and **f** posture for stool retention. **g** The proportion of children meeting the diagnostic criteria for functional constipation (>2 symptoms). Measurements were obtained at Days 0, 7, and 28 in the Placebo, Kids, and PregMom groups. Within-group comparisons across time points were performed using the Cochran test. Between-group comparisons at the same time point were assessed using two-sided Fisher’s exact tests or two-sided Chi-square tests (**a–g**). The two-sided significance threshold was set at *p* < 0.05 and adjusted to *p* < 0.0167 and *p* < 0.0083 for within-group and between-group comparisons, respectively, to account for multiplicity across the three prespecified primary outcomes. Sample size: *n* = 34, 37, and 36 biologically independent participants for the Placebo, Kids, and PregMom groups, respectively. ARR (absolute risk reduction) denotes the difference in outcome rates between the probiotic and Placebo groups; NNT (number needed to treat) is the reciprocal of ARR and represents the number of children who need to be treated to prevent one additional case; OR (odds ratio) represents the odds of improvement in the probiotic groups relative to the Placebo group.

Assessed by 6 common symptoms (Rome IV G7 criteria), constipation was alleviated after 7 days of probiotics use, with more pronounced improvement seen on day 28 (Fig. 2). Thus, the percentage of children with bowel movements ≤2 times/week decreased significantly after 28 days, by 3.60-fold (*p* = 0.0013) and 4.00-fold (*p* = 0.0007) in the Kids and the PregMom groups, respectively, compared to a non-significant 1.25-fold decrease in the Placebo. The odds ratio (OR) for this improvement was 3.49 (95% CI: 1.12–10.05; *p* = 0.0317) and 4.36 (95% CI: 1.26–13.37; *p* = 0.0226), respectively (Fig. 2a and Supplementary Table 9). Similar trends were observed for all six indexes, with the highest efficacy quantitated by OR was observed in the PregMom group, followed by the Kids group, and the lowest in the Placebo group (Fig. 2b–f and Supplementary Table 9).

The change in the proportion of children with constipation conditions (defined as having two or more symptoms following ROME IV G7 criteria) was further quantitated in Results depicted in Fig. 2g which demonstrate significantly (*p* < 0.0001) reduced proportion of children having constipation on days 7 and 28 in the probiotics groups, even after applying alpha adjustment (*p* < 0.0167). In particular, at day 28, the decrease was 3.70 and 5.14-fold in the Kids and the PregMom groups, respectively. In comparison, the Placebo group revealed much lower recovering (5.88% at day 7 and 20.59% at day 28; *p* = 0.003). After 28 days of treatment, the ARR for constipation was 52.38% (95% CI: 35.45–77.26%) and 59.97% (95%CI: 44.48–84.68%) in Kids and PregMom groups, respectively (Fig. 2g and Supplementary Table 10). The NNT indicates that treating two children with LiveSpo Preg-Mom or LiveSpo Kids would prevent one additional case of constipation. Notably, these between-group differences (*p* < 0.0001) remained statistically significant after Bonferroni correction for multiplicity across the three prespecified primary outcomes (two-sided *α* = 0.0083). In conclusion, LiveSpo Preg-Mom, and to a less extend LiveSpo Kids, efficiently alleviated constipation, with effects observed as early as after 7 days of use.

In addition to relieving constipation, the probiotic intervention improved appetite-related signs in children with baseline anorexia (Supplementary Fig. 8). By day 28, both Kids and PregMom groups had higher odds of eating faster (Supplementary Fig. 8a and Supplementary Table 9), while the PregMom group also showed significant improvements in eating with better appetite (Supplementary Fig. 8b) and having meal times <30 min (Supplementary Fig. 8c and Supplementary Table 9) versus Controls (*p* values = 0.0016). After Bonferroni adjustment for the three primary outcomes (*p* < 0.0167), improvements in “eating with better appetite” and “meal time <30 min” in the PregMom group remained statistically significant, whereas increases in the remaining indicators showed positive trends but did not reach the adjusted significance threshold. Taken all the data for anorexia, within-group reductions from baseline remained significant even after adjustment, indicating a consistent improvement over time during the intervention (Supplementary Fig. 8d). The proportion with anorexia falls markedly, with an ARR of 24.40% (95% CI: 3.09–49.44%) for Kids (*p* = 0.0390) and 25.98% (95% CI: 4.69–51.25%) for PregMom (*p* = 0.0292), and an NNT of 4 for either probiotic (Supplementary Fig. 8d and Supplementary Table 10). These between-group differences did not meet the Bonferroni-adjusted threshold (two-sided *α* = 0.0083). Weight gain outcomes also improved (Supplementary Fig. 9). By day 28, both probiotic groups gained 0.30 kg compared to no gain in Controls (Supplementary Fig. 9a) and had significant greater median WAZ increases (Supplementary Fig. 9b) (all *p* values < 0.0001), which remained significant after Bonferroni adjustment (*p* < 0.0167). The proportion of children with underweight or at risk of underweight decreased significantly in the PregMom group (ARR: 19.30%; 95% CI: 1.70–37.50%; *p* = 0.0281) and non-significantly in the Kids group (ARR: 7.87%; 95% CI: 0–23.80%; *p* = 0.3591) (Supplementary Fig. 9c and Supplementary Table 10). No statistically significant between-group differences were observed after Bonferroni adjustment (two-sided *α* = 0.0083). Similarly, the proportion of children who were underweight only also declined in both probiotic intervention groups, although the changes were not statistically significant (Supplementary Fig. 9d). These findings suggest *Bacillus* spore probiotics support appetite, weight gain, and reduced underweight risk, with greater effects observed in the PregMom group.

### Modulation of blood cytokine profile and fecal IgA by *Bacillus* probiotics

**Fig. 3:**
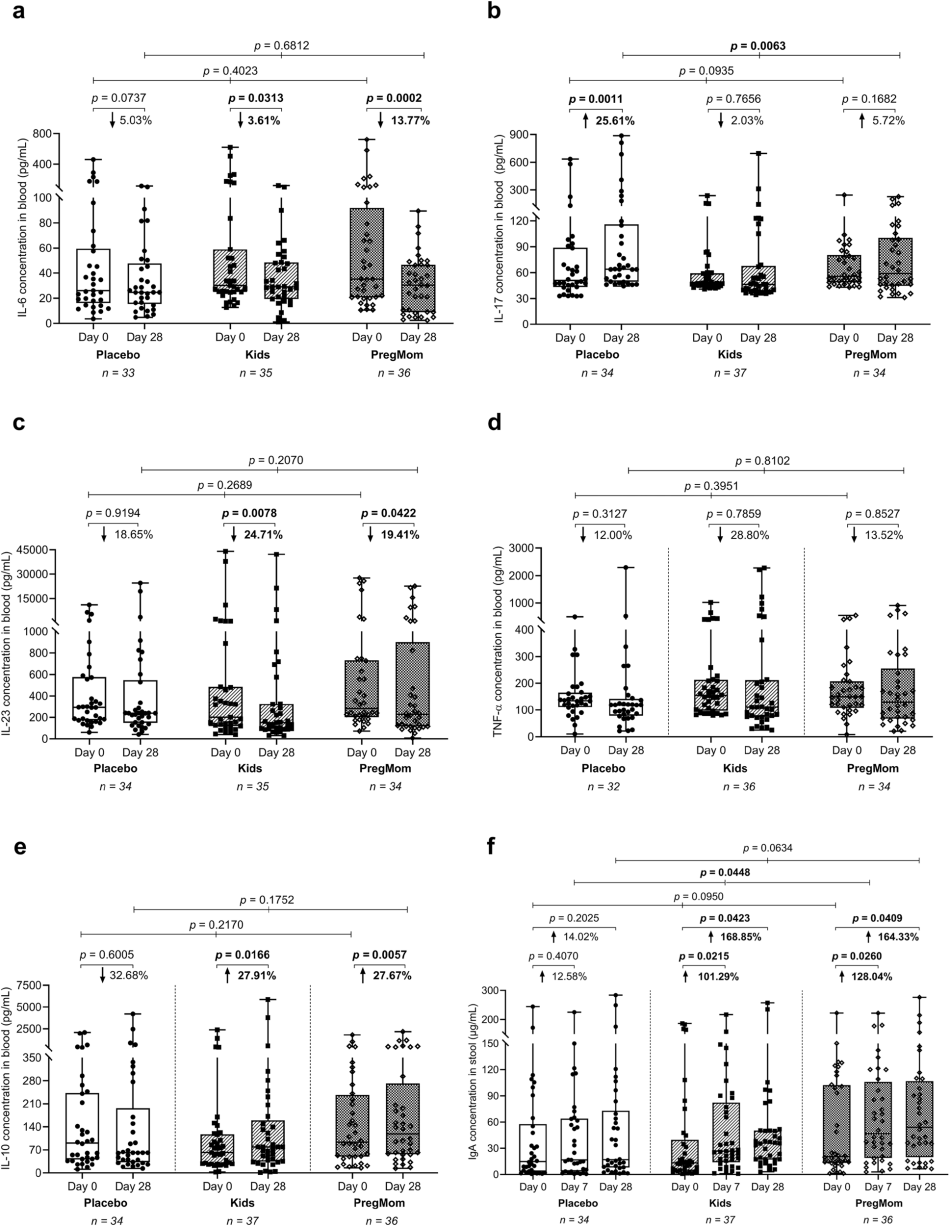
Effects of probiotic interventions on reducing blood pro-inflammatory cytokines and increasing anti-inflammatory IL-10 and fecal IgA in children with functional constipation. Pro-inflammatory cytokine levels (IL-6, IL-17, IL-23, and TNF-α) (**a**–**d**) and anti-inflammatory IL-10 levels (**e**) in blood samples at day 28 compared with day 0 within the same group. Fecal IgA levels (**f**) at days 7 and 28 compared with day 0 within the same group. Differences between two time points within a group were assessed using the two-sided Wilcoxon signed-rank test, while differences among the three groups at the same time points were assessed using the Kruskal–Wallis test, with significance set at *p* < 0.05 (**a**–**f**). Sample sizes: biologically independent samples for Placebo, Kids, and PregMom were: 33, 35, and 36 for IL-6; 34, 37, and 34 for IL-17; 34, 35, and 34 for IL-23; 32, 36, and 34 for TNF-α; 34, 37, and 36 for IL-10; and 34, 37, and 36 for stool IgA.

To support the clinical data, we assessed the effects of the two *Bacillus* spore probiotics on blood markers of inflammation, focusing on changes in key pro-inflammatory cytokines (IL-6, IL-17, IL-23, and TNF-α) from day 0 to day 28. As summarized in Fig. 3a–d, the probiotics yielded significant reductions in the levels of IL-6 and IL-23 at day 28 vs day 0, while that of TNF-α showed only non-significant trends of decrease. Specifically, at day 28 compared to day 0, there was significant reductions in the levels of IL-6 by 13.77% (*p* = 0.0002) in the PregMom group (Fig. 3a), and of IL-23 by 24.71% (*p* = 0.0078) and 19.41% (*p* = 0.0422) in the Kids and PregMom groups, respectively (Fig. 3c). In contrast to the Placebo group which showed significantly (*p* = 0.0011) increased IL-17 level during the follow-up, this cytokine level remained almost unchanged in the two probiotic groups (Fig. 3b).

Along with the decrease in pro-inflammatory markers, the increase in blood anti-inflammatory cytokine IL-10 and fecal mucosal IgA may provide indicators of favorable shifting the immune balance. Thus, at day 28, the Kids and PregMom groups exhibited increases of IL-10, 27.91% (*p* = 0.0166) and 27.67% (*p* = 0.0057), respectively, versus a nonsignificant trend of 33.68% decrease in the Placebo (Fig. 3e). Furthermore, there was a strong and statistically significant induction of IgA (101.29–168.85%) in both Kids and PregMom groups at days 7 and 28, while the change in IgA level in the Placebo group was modest (12.58–14.02%) and was not significant (Fig. 3f).

Overall, daily supplementation with LiveSpo Kids and Preg-Mom for 28 days was associated with a remarkable shift of the systemic cytokine profile toward reducing the pro-inflammatory IL-6 and IL-23, alleviating the IL-17 activation, while increasing the anti-inflammatory IL-10 and fecal IgA compared to the Placebo group.

### Restoration of gut microbiota with *Bacillus* probiotics

**Fig. 4:**
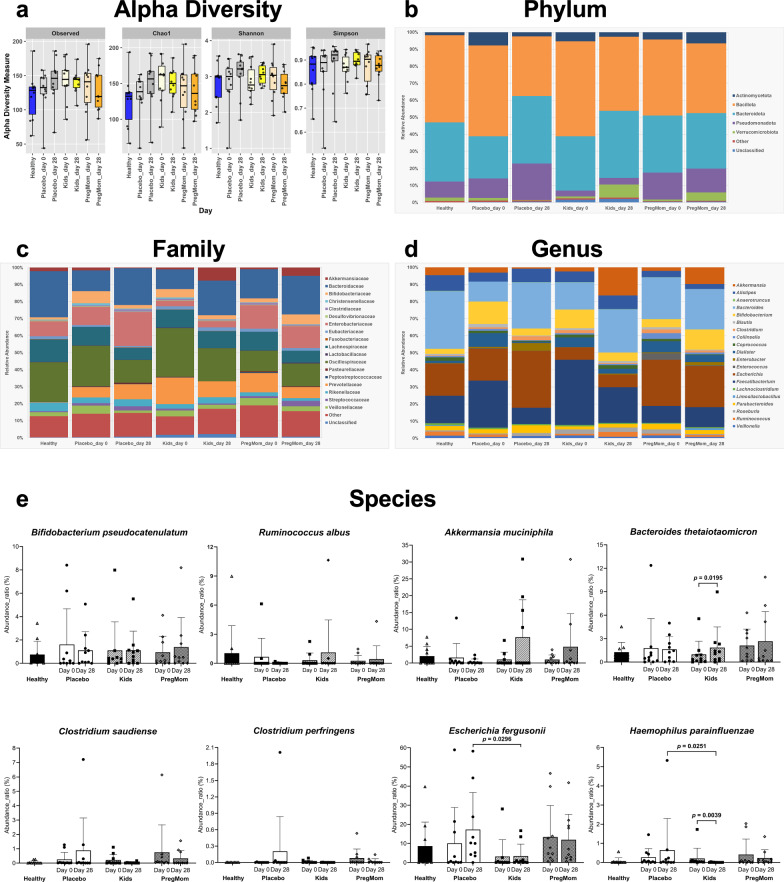
Effects of probiotic interventions on gut microbiota, including alpha diversity and the distribution of major phyla, families, genera, and species in stool samples based on 16S rRNA metagenomic analysis. **a** Alpha diversity; **b**–**d** distributions of major phyla, families, and genera in stool samples comparing Control, Kids, and PregMom groups at day 28 versus day 0, with the Healthy group used as reference; **e** changes in the eight most abundant native gut-associated species in the Control, Kids, and PregMom groups at day 28 versus day 0, also referenced to the Healthy group. The differences between two time points within a group were assessed using the two-sided signed-rank Wilcoxon test, while differences among the two groups at the same time points were assessed using the two-sided Mann-Whitney test, with significance set at *p* < 0.05. Sample size: *n* = 10 biologically independent samples per group per time point (Healthy, Placebo, Kids, PregMom) (**a**–**e**). Abundance ratio (%) refers to the relative abundance of each bacterial species relative to the total microbial reads within each stool sample.

Gut microbiota changes in children with constipation after 28 days of probiotic or placebo use were analyzed compared to the reference Healthy group. Before intervention, alpha diversity indices (OTU, Chao1, Shannon, and Simpson) were slightly higher in children with constipation than in healthy children. After 28 days, OTU and Chao1 indices showed a non-significant increase in Placebo, but a non-significant decrease in the Kids and PregMom (Fig. 4a). Beta diversity analysis revealed no clear clustering among groups (Supplementary Fig. 10).

At the phylum level (Fig. 4b), the five main phyla—Bacillota, Bacteroidota, Pseudomonadota, Actinomycetota, and Verrucomicrobiota—were detected in the Healthy group with decreasing relative abundances. By day 28, in Placebo, Bacillota, Actinomycetota, and Verrucomicrobiota, and decreased 1.52-, 3.14-, 3.88-fold, while Bacteroidota and Pseudomonadota increased 1.60- and 1.86-fold, respectively. The Placebo group diverged from the microbiota composition observed in Healthy group, whereas the two probiotic groups remained more similar at the phylum level. The probiotic groups maintained stable phyla abundances, except for Verrucomicrobiota, which increased in both Kids (7.31-fold) and PregMom (4.65-fold). Actinomycetota declined in Kids (1.98-fold) but increased in PregMom (1.56-fold), remained within the relative range observed in Healthy group.

At the family and genus levels (Fig. 4c, d), by day 28, *Bifidobacteriaceae* increased 2.47-fold in PregMom but decreased 3.33-fold in Placebo. *Akkermansiaceae* and *Akkermansia* increased in Kids (7.31-fold) and PregMom (4.70-fold) but declined in Placebo (3.88-fold). In contrast, *Clostridiaceae* (*Clostridium*) and *Pasteurellaceae* (*Haemophilus*) increased in Placebo (2.48- and 2.43-fold) but decreased in Kids (1.80- and 31.09-fold) and PregMom (1.86- and 1.76-fold), approaching the levels observed in the Healthy group. *Ruminococcus* decreased the most in Placebo (8.93-fold) but increased in Kids (3.01-fold) and remained unchanged in PregMom, reaching levels close to those in Healthy children. *Escherichia* increased 1.72-fold in Placebo with no significant change in the probiotic groups.

Regarding beneficial species (Fig. 4e, upper lane), *Bifidobacterium pseudocatenulatum* remained stable in the Kids group and increased 1.48-fold in PregMom, while decreasing in Placebo. *Ruminococcus albus* declined sharply in Placebo (26.39-fold) but increased in PregMom (1.68-fold) and Kids (3.48-fold). *Akkermansia muciniphila* dropped in Placebo (3.88-fold) but increased in PregMom (4.70-fold) and Kids (7.31-fold). *Bacteroides thetaiotaomicron* decreased slightly in Placebo but increased in Kids (1.88-fold; *p* = 0.0195) and PregMom (1.26-fold). Harmful species (Fig. 4e, lower lane) tended to increase in Placebo but decrease with probiotics. *Clostridium saudiense* increased 3.52-fold in Placebo but declined in PregMom (2.32-fold) and Kids (8.60-fold). *Clostridium perfringens* increased 176.46-fold in Placebo, while Kids and PregMom showed reductions of 31.55-fold and 3.39-fold, respectively. *Haemophilus parainfluenzae* increased 2.42-fold in Placebo but decreased significantly in Kids (31.09-fold, *p* = 0.0039) and PregMom (1.76-fold). Taken together, our data suggest favorable microbiota modulation characterized by an increase in beneficial bacteria and a reduction in potentially harmful ones, supporting the probiotic’s role in restoring gut microbial balance despite the limited sample size.

## Discussion

This study is the first community-based, multi-center, randomized, double-blind, controlled trial to evaluate the safety and efficacy of multi-strain *Bacillus* probiotics in children with functional constipation, supported with analysis of immunomodulation and gut microbiota modulation as background mechanisms of clinical effects. All early withdrawals occurred within the first few days due to a lack of final family consensus rather than any product-related issue, making ITT analysis unfeasible. Consequently, only PP analysis was performed, with minimal risk of substantial bias given the small proportion (3.6%) of withdrawals. Moreover, as withdrawals were more frequent in the placebo group (8.1% vs. 0–2.7% in the probiotic groups), any potential bias would likely reduce rather than exaggerate the observed efficacy. Both tested probiotics were well-tolerated, with no adverse reactions reported during treatment, indicating their safety for children and supporting previous findings^29,30,36–38^. Although intervention products were labeled with simple codes (A/B/C), which could theoretically introduce perceptual bias at the participant level (e.g., alphabetical ranking effects), allocation concealment was ensured by an independent staff member with exclusive access to the randomization list. Both participants and study staff remained fully blinded until database lock, thereby minimizing the likelihood of meaningful selection or allocation bias.

The two multi-strain *Bacillus* spore probiotics in our study demonstrated a significant alleviation of constipation symptoms and reduced proportion of children with constipation within 7 days, with stronger effects by day 28. Some improvement in constipation symptoms was also observed in the placebo group, which may be attributed to the dietary and hydration advice provided to all participants at baseline. Such lifestyle adjustments are known to alleviate mild constipation even in the absence of probiotic intervention. Although the trial was not designed for direct comparison between probiotic formulations, placebo-adjusted improvements appeared numerically larger in the LiveSpo Preg-Mom group (three strains) than in LiveSpo Kids (two strains). This pattern may reflect a potential additive contribution of *B. coagulans* when combined with *B. subtilis* and *B. clausii*. These findings provide evidence of clinically meaningful improvements within 7–28 days, thereby helping to address the limited data on spore-forming probiotics for childhood constipation highlighted in recent systematic reviews^13^. Given the rapid onset of symptom relief observed by day 7, these *Bacillus*-based probiotics may represent a promising alternative or adjunct to conventional laxatives in primary care, particularly for children with recurrent symptoms or intolerance to pharmacological options.

Our findings also show that, in children with constipation accompanied by anorexia and risk of underweight, *Bacillus* spore probiotics significantly reduced anorexia, with a tendency toward stronger effects in the 3-strain LiveSpo PregMom rather than in the 2-strain LiveSpo Kids, compared with the placebo. This anorexia-improving effect is consistent with a recent meta-analysis of 26 randomized controlled trials (1536 participants) showing that probiotic supplementation can improve appetite^39^. Furthermore, the improvements in weight gain and the reduced risk of underweight further underscore the additional benefits of *Bacillus* spore probiotics in this population, in line with findings from several other studies^40,41^.

Most probiotic studies on constipation focus on clinical outcomes, with few examining gut microbiota and mucosal immunity. These factors regulate immune balance and gut health through macrophage activation, IgA stimulation, and cytokine modulation^42^. Our study found a significant induction of fecal IgA by probiotics early as on day 7, suggesting an enhanced mucosal protection^9,43^. Moreover, elevated IL-10 levels alongside reductions in IL-6^44^ and IL-23, and suppression of IL-17 expression, suggest that probiotics may exert anti-inflammatory effects, potentially through downregulation of the Th17 pathway^42,45^. These immunological effects may represent one mechanism by which probiotics alleviate symptoms of functional constipation. Increased Th2-driven IgA production may strengthen mucosal barrier protection by stabilizing the mucus layer and reducing local inflammation, while modulation of Th17-associated cytokines (reduced IL-6, IL-17, IL-23 and increased IL-10) indicates a shift toward an anti-inflammatory milieu. Together, these changes could improve intestinal function and contribute to the observed relief of constipation symptoms.

The gut microbiota plays key role in the onset and development of functional constipation^46^. While *Lactobacillus* and *Bifidobacterium* probiotics have been shown to improve gut microbiota, probiotic effects in children with constipation remain unstudied^13^. To the best of our knowledge, we for the first time demonstrated that *Bacillus* spore probiotics improve gut microbiota in children with functional constipation, in particular via stimulating beneficial genera (*Ruminococcus, Akkermansia, Bifidobacterium*) and reducing the harmful ones (*Clostridium, Haemophilus*). Increases in *A. muciniphila* and recovery of *Ruminococcus* species may contribute to a healthier intestinal environment, in line with previous studies linking these taxa to mucus stabilization and short-chain fatty acids (SCFAs)-mediated gut motility^46,47^. Nevertheless, since SCFAs were not measured in our study, this interpretation should be considered exploratory and warrants further investigation. Despite the limited microbiome sample size, the microbiota results appear to align with the clinical and immunological data.

Real-time PCR detection of *Bacillus* DNA in stool samples confirms product compliance. The greater relative efficacy observed for the Preg-Mom formulation may reflect differences in strain composition, though specific functional contributions were not assessed in this study and require further investigation. Strengths of this study include its robust design and comprehensive integration of clinical, immunological, and microbiological endpoints. Despite the study’s strengths, several limitations warrant acknowledgement. First, anorexia in this study was defined according to the diagnostic criteria of the Vietnam National Institute of Nutrition, as no globally standardized definition currently exists for pediatric anorexia in otherwise healthy children. These criteria, including food refusal, selective eating, prolonged eating (>30 min), or intake <50% of age-recommended needs, are widely applied in community-based pediatric nutrition research in Vietnam and were considered the most appropriate for this population. Although anorexia was prespecified as one of the primary outcomes to capture broader child health benefits alongside constipation, this locally adapted definition may limit comparability with studies using different diagnostic frameworks and reduce the generalizability of appetite-related findings. The findings on anorexia improvement are meaningful in the subgroup of children with constipation and risk of underweight, but should be interpreted with caution for children in general, and require validation in future trials specifically designed for anorexia outcomes. Second, although all participants received standardized advice on fiber intake and hydration, actual consumption was not systematically recorded. Therefore, residual confounding by diet cannot be fully excluded. Third, adherence to lifestyle recommendations may have varied between groups and was not objectively assessed. Fourth, although placebo and probiotic ampoules were visually identical and opaque, administration occurred at home without direct supervision; therefore, variability in product handling or adherence cannot be fully excluded and may represent a potential source of performance bias, with treatment recognition likely minimal. Fifth, the number of children with both anorexia and underweight was small, and microbiota sequencing was performed in only 10 participants per group for each time point, limiting statistical power and generalizability. Accordingly, these exploratory findings should be interpreted cautiously. Finally, although the 28-day follow-up does not provide information on the long-term durability of the effects or relapse rates, future studies should include extended follow-up and larger cohorts to further assess sustained efficacy and long-term outcomes. Future research should include larger cohorts, extended follow-up, and mechanistic studies to clarify sustained efficacy and inform optimized, potentially personalized, probiotic strategies for children with functional constipation.

In conclusion, the studied multi-strain *Bacillus* spore probiotics were well tolerated, revealing no adverse effects. They demonstrated rapid improvements within 28 days in functional constipation, with greater relative efficacy observed in LiveSpo Preg-Mom (3 *Bacillus* strains), followed by LiveSpo Kids (2 *Bacillus* strains). Notably, both probiotic groups exhibited clear patterns of immunomodulatory effects, gut microbiota restoration by boosting beneficial and reducing harmful genera. This probiotic therapy may represent a promising intervention for functional constipation, particularly in settings where access to long‑term pharmacological options is limited. Longer‑term trials with controlled dietary monitoring and larger microbiota samples are warranted to confirm sustainability and dissect strain‑specific mechanisms.

## Supplementary information

Supplementary Information

Description of Additional Supplementary files

Supplementary Data 1

Supplementary Data 2

Supplementary Data 3

## Data Availability

All sequencing data generated in this study have been deposited in the National Center for Biotechnology Information (NCBI) under BioProject accession PRJNA1365323, available at https://www.ncbi.nlm.nih.gov/bioproject/PRJNA1365323/. Additional materials, including the full Study Protocol and Statistical Analysis Plan, are available from the corresponding author (vananhbiolab@gmail.com) upon reasonable request and with approval from the National Institute of Nutrition and the Spobiotic Research Center, ANABIO R&D Ltd. Requests will be reviewed within 2–3 weeks. Access is limited to academic, non-commercial research purposes only, subject to applicable ethical and confidentiality requirements.
