# Supplementary material for: Bacillus spore probiotics for alleviating functional constipation in children: a randomized, double-blind, placebo-controlled trial
Source: Commun Med (Lond). 2026 Mar 18;6:148. doi: 10.1038/s43856-026-01517-6 (PMC13000159; doi:10.1038/s43856-026-01517-6)
Supplement: Supplementary file 1 — Supplementary Information [file 43856_2026_1517_MOESM1_ESM.pdf]

Supplementary Information

Bacillus Spore Probiotics for Alleviating Functional Constipation in Children: A Clinical Trial

---

Table of contents

I. Supplementary figures 2
II. Supplementary tables 12

I. Supplementary figures

a

Description	Max Score	Total Score	Query Cover	E value	Per. Ident	Accession
Bacillus subtilis strain HU58 16S ribosomal RNA (rrnE) gene, partial sequence	2649	2649	100%	0.0	100.00%	EF101709.1
Bacillus subtilis strain CH14 16S ribosomal RNA gene, partial sequence	2639	2639	100%	0.0	99.79%	KM492823.1
Bacillus subtilis strain RSA5 16S ribosomal RNA gene, partial sequence	2636	2636	99%	0.0	99.86%	JQ887981.1
Bacillus sp. strain ZJ-5 16S ribosomal RNA gene, partial sequence	2634	2634	100%	0.0	99.72%	KY283146.1
Bacillus subtilis strain GXG-5 16S ribosomal RNA gene, partial sequence	2634	2634	100%	0.0	99.72%	KY711183.1
Bacillus subtilis strain M015 16S ribosomal RNA gene, partial sequence	2634	2634	100%	0.0	99.72%	KP192484.1
Bacillus sp. JZ252 16S ribosomal RNA gene, partial sequence	2634	2634	100%	0.0	99.72%	KU312057.1
Bacillus subtilis strain NAP4 16S ribosomal RNA gene, partial sequence	2634	2634	99%	0.0	99.79%	KJ872855.1
Bacillus subtilis strain FJM14 16S ribosomal RNA gene, partial sequence	2634	2634	100%	0.0	99.72%	KR493016.1
Bacillus sp. M28(2014) 16S ribosomal RNA gene, partial sequence	2634	2634	99%	0.0	99.79%	KM925012.1

b

Description	Scientific Name	Max Score	Total Score	Query Cover	E value	Per. Ident	Acc. Len	Accession
Bacillus subtilis strain DSM 10 16S ribosomal RNA, partial sequence	Bacillus subtilis	2494	2494	100%	0.0	100.00%	1517	NR_027552.1
Bacillus subtilis strain JCM 1465 16S ribosomal RNA, partial sequence	Bacillus subtilis	2494	2494	100%	0.0	100.00%	1472	NR_113265.1
Bacillus subtilis strain NBRC 13719 16S ribosomal RNA, partial sequence	Bacillus subtilis	2494	2494	100%	0.0	100.00%	1475	NR_112629.1
Bacillus subtilis subsp. inaquosorum strain BGSC 3A28 16S ribosomal RNA, partial sequence	Bacillus inaquosorum	2488	2488	100%	0.0	99.93%	1538	NR_104873.1
Bacillus subtilis strain IAM 12118 16S ribosomal RNA, complete sequence	Bacillus subtilis	2488	2488	100%	0.0	99.93%	1550	NR_112116.2
Bacillus subtilis strain BCRC 10255 16S ribosomal RNA, partial sequence	Bacillus subtilis	2488	2488	100%	0.0	99.93%	1468	NR_116017.1
Bacillus tequilensis strain 10b 16S ribosomal RNA, partial sequence	Bacillus tequilensis	2488	2488	100%	0.0	99.93%	1456	NR_104919.1
Bacillus subtilis subsp. subtilis strain 168 16S ribosomal RNA, complete sequence	Bacillus subtilis subsp. subtilis	2483	2483	100%	0.0	99.85%	1550	NR_102783.2
Bacillus subtilis subsp. spizizenii strain NRRL B-23049 16S ribosomal RNA, partial sequence	Bacillus spizizenii	2483	2483	100%	0.0	99.85%	1409	NR_024931.1
Bacillus subtilis subsp. spizizenii strain NBRC 101239 16S ribosomal RNA, partial sequence	Bacillus spizizenii	2483	2483	100%	0.0	99.85%	1475	NR_112686.1

c

Description	Max Score	Total Score	Query Cover	E value	Per. Ident	Accession
Bacillus clausii strain BRM043935 16S ribosomal RNA gene, partial sequence	2547	2547	100%	0.0	99.57%	MH305350.1
Bacillus clausii strain ENTPro, complete genome	2547	17759	100%	0.0	99.57%	CP012475.1
Bacillus clausii strain ANA38 16S ribosomal RNA gene, partial sequence	2542	2542	100%	0.0	99.50%	MT110681.1
Bacillus clausii strain ANA37 16S ribosomal RNA gene, partial sequence	2542	2542	100%	0.0	99.50%	MT110679.1
Bacillus clausii strain ANA36 16S ribosomal RNA gene, partial sequence	2542	2542	100%	0.0	99.50%	MT107136.1
Bacillus clausii strain ANA35 16S ribosomal RNA gene, partial sequence	2542	2542	100%	0.0	99.50%	MT107086.1
Bacillus clausii strain SL4-4 16S ribosomal RNA gene, partial sequence	2542	2542	100%	0.0	99.50%	MK312486.1
Bacillus rhizosphaerae strain WA12 16S ribosomal RNA gene, partial sequence	2542	2542	100%	0.0	99.50%	KT595230.1
Bacillus clausii strain E2 16S ribosomal RNA gene, partial sequence	2542	2542	100%	0.0	99.50%	EU117277.1
Bacillus clausii KSM-K16 DNA, complete genome	2542	17715	100%	0.0	99.50%	AP006627.1

d

Description	Max Score	Total Score	Query Cover	E value	Per. Ident	Accession
Bacillus coagulans 16S ribosomal RNA gene, partial sequence	2767	2767	99%	0.0	99.79%	KX028863.1
Bacillus coagulans gene for 16S rRNA, partial sequence, strain: NBRC 12714	2759	2759	99%	0.0	99.65%	AB680332.1
Bacillus coagulans strain DSM 2314 chromosome, complete genome	2753	27388	99%	0.0	99.65%	CP033687.1
Bacillus coagulans strain IDCC1201 chromosome, complete genome	2753	27397	99%	0.0	99.65%	CP035305.1
Bacillus coagulans LA204, complete genome	2753	24651	99%	0.0	99.65%	CP025437.1
Bacillus coagulans strain R11 chromosome, complete genome	2753	27359	99%	0.0	99.65%	CP026649.1
Bacillus coagulans strain LBSC chromosome	2753	24715	99%	0.0	99.65%	CP022701.1
Bacillus coagulans strain BC-HY1, complete genome	2753	27330	99%	0.0	99.65%	CP017888.1
Bacillus coagulans strain N83 16S ribosomal RNA gene, partial sequence	2753	2753	99%	0.0	99.72%	KX010086.1
Bacillus coagulans strain S-lac, complete genome	2753	27419	99%	0.0	99.65%	CP011939.1

Supplementary Figure 1. BLAST analysis of *Bacillus* strains used in LiveSpo Preg-Mom and LiveSpo Kids
(**a**) *Bacillus subtilis* ANA3, (**b**) *Bacillus subtilis* ANA46, (**c**) *B. clausii* ANA39, and (**d**) *Bacillus coagulans* ANA40.

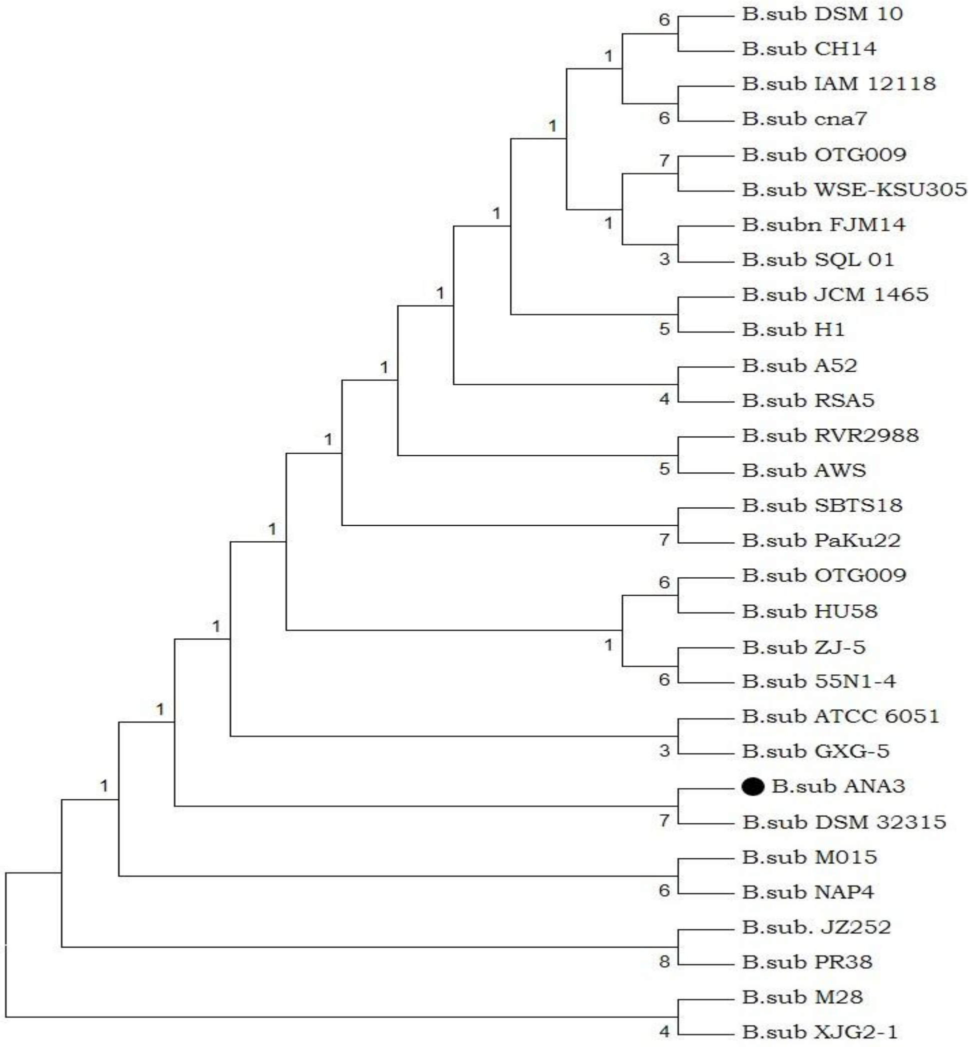

Supplementary Figure 2. Phylogenetic tree of *B. subtilis* ANA3
A maximum-likelihood phylogenetic tree was constructed based on 16S rRNA gene sequences from representative *B. subtilis* strains. *B. subtilis* ANA3 (indicated by a black dot) clusters within the *B. subtilis* clade and shows close relatedness to *B. subtilis* DSM 32315 strain. Numbers at the nodes represent bootstrap support values, indicating strong confidence for the branching patterns.

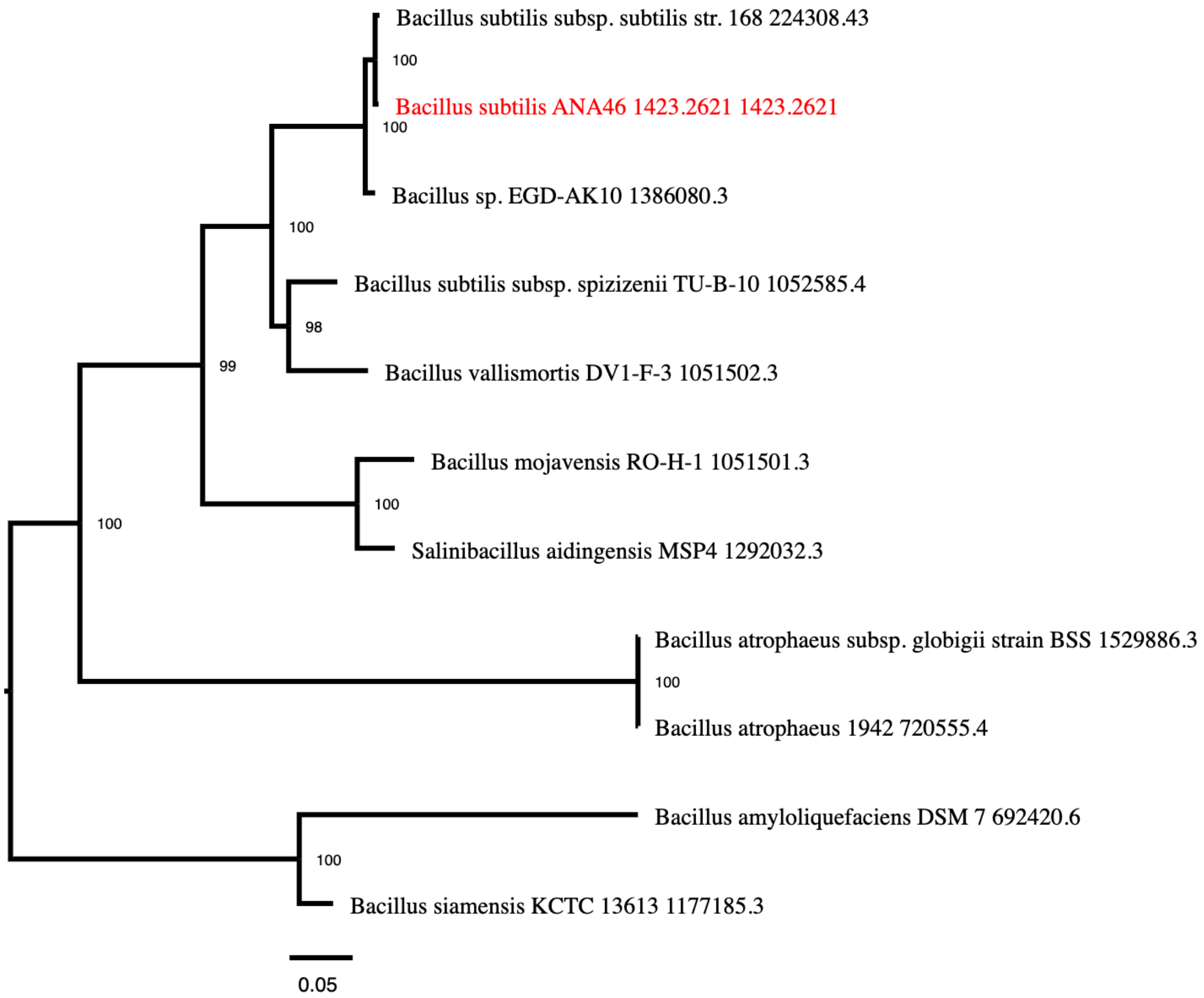

Supplementary Figure 3. Phylogenetic tree of *B. subtilis* ANA46

Phylogenetic tree based on 16S rRNA sequences demonstrating that *B. subtilis* ANA46 (highlighted in red) clusters tightly with *B. subtilis* subsp. subtilis strain 168, supported by 100% bootstrap confidence, indicating high genetic similarity and confirming accurate taxonomic classification. Scale bar represents 0.05 nucleotide substitutions per site.

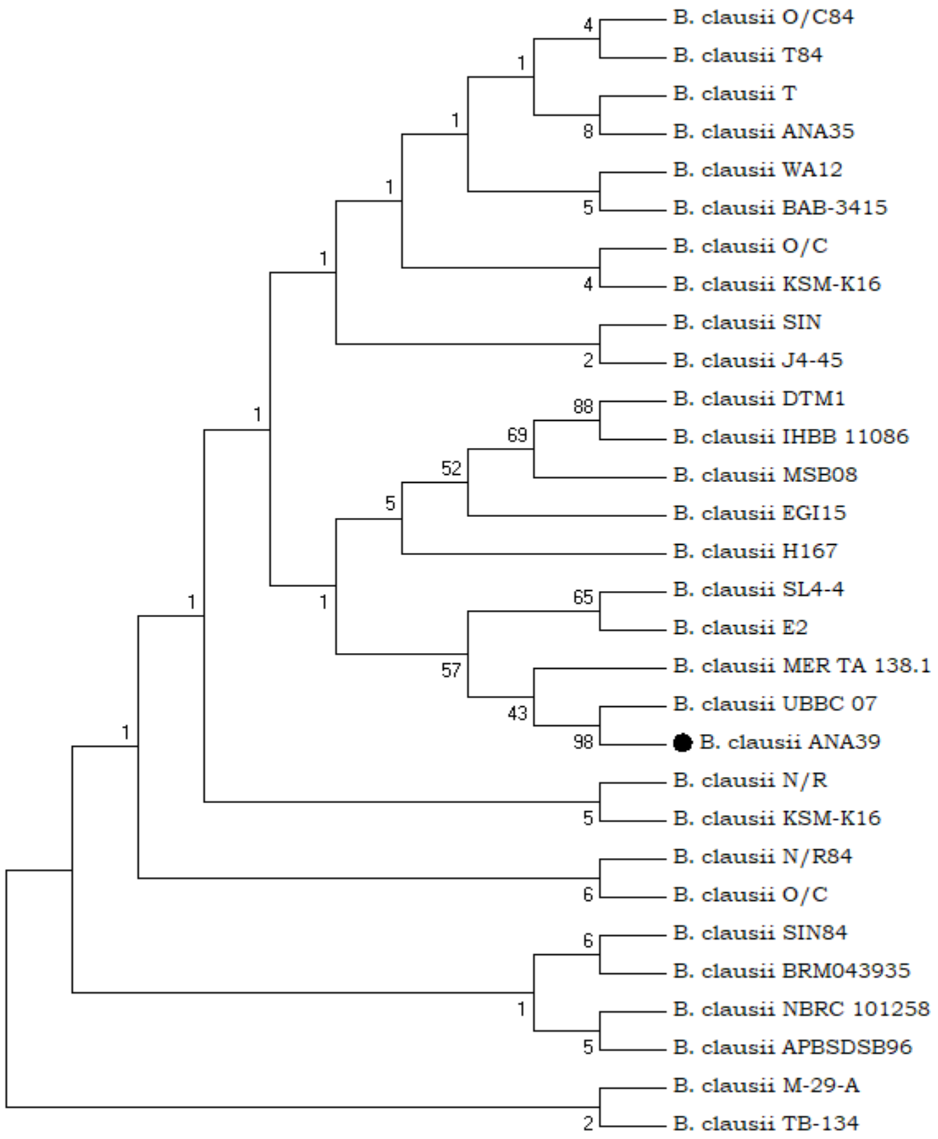

Supplementary Figure 4. Phylogenetic tree of *B. subtilis* ANA39

Phylogenetic tree based on 16S rRNA sequences of representative *B. clausii* strains. The strain ANA39 (●) clustered closely with *B. clausii* UBBC07, supported by high bootstrap values (98%), confirming its taxonomic position within the *B. clausii* species.

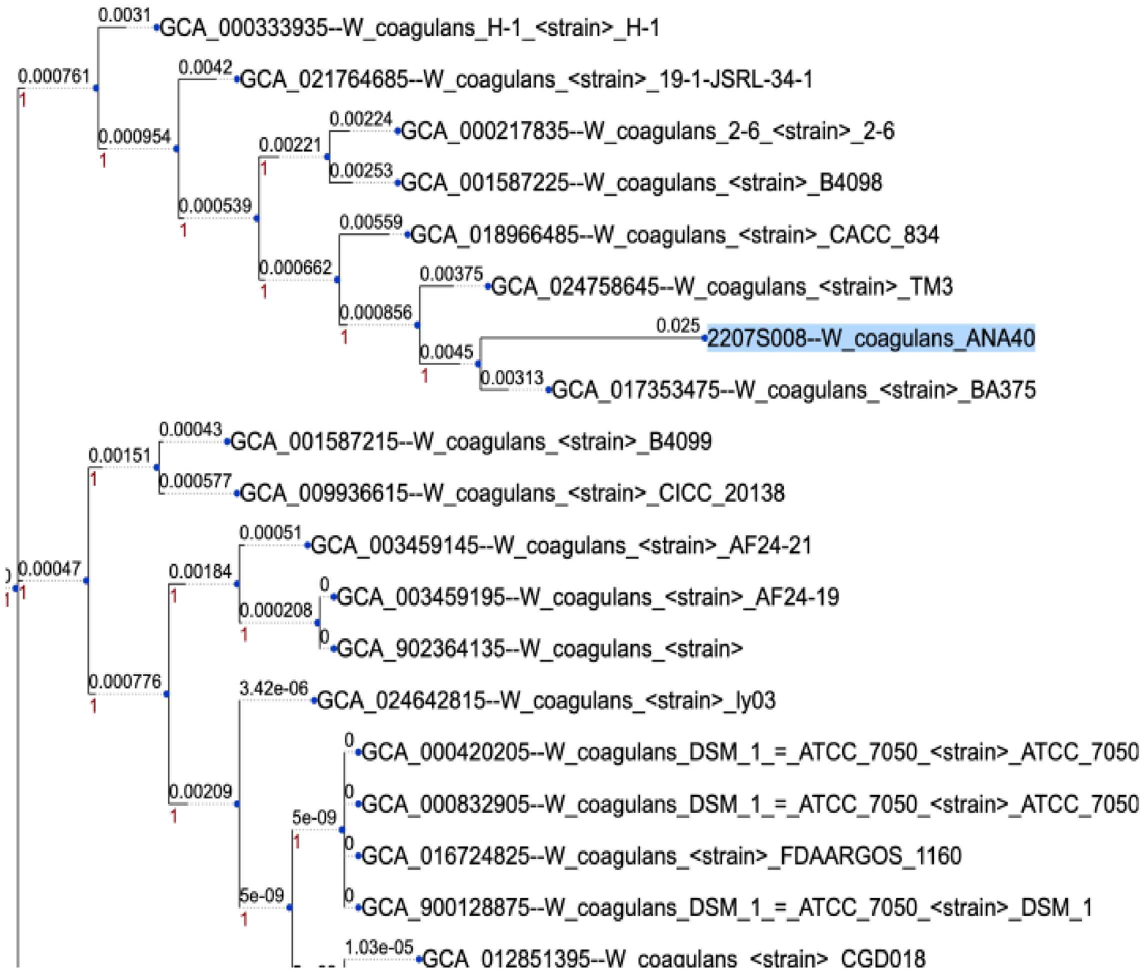

Figure S5. Phylogenetic tree for *Bacillus coagulans* ANA40

Supplementary Figure 5. Phylogenetic tree of *B. coagulans* ANA40

Whole-genome phylogenetic tree of representative *B. coagulans* strains; ANA40 (highlighted in blue) clusters with TM3 and BA375, confirming species assignment. Scale bar denotes nucleotide substitutions per site.

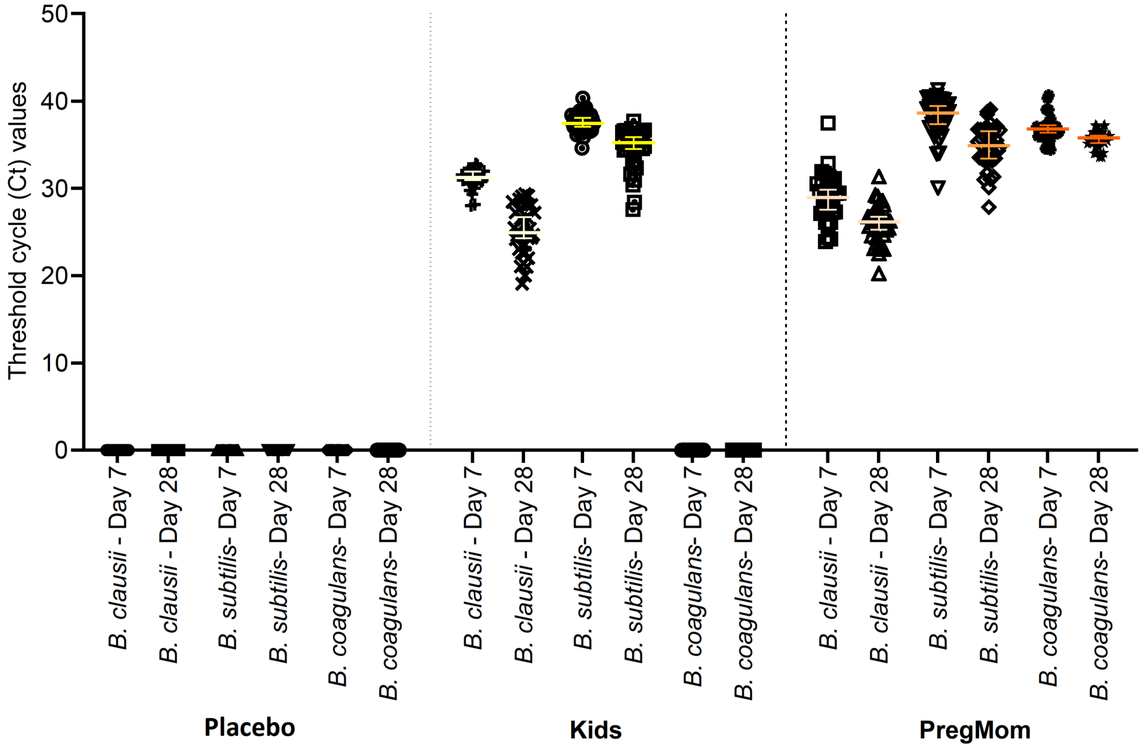

Supplementary Figure 6. Threshold cycle (C_t) of fluorescent signals for *B. subtilis*, *B. clausii*, and *B. coagulans* measured in stool samples of Placebo, Kids, and PregMom groups at day 7 and 28.

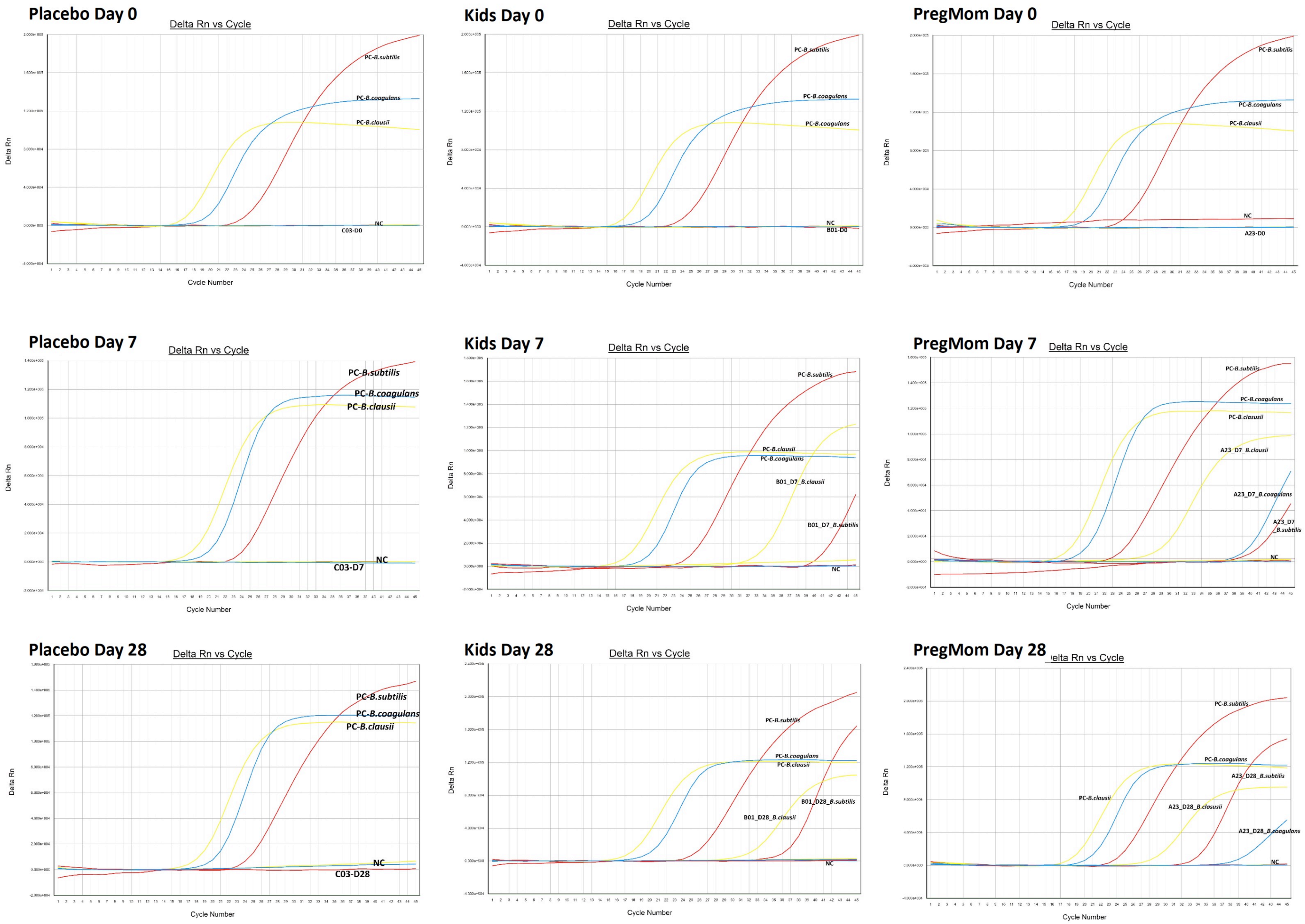

Supplementary Figure 7. Real-time PCR SYBR Green amplification curves specifically for *B. subtilis*, *B. clausii*, and *B. coagulans* measured in stool samples of Placebo, Kids, and PregMom groups at day 7 and 28.

Two representative stool samples of Placebo (Placebo 1, 2), Kids (Kids 1, 2), and PregMom (PregMom 1, 2) groups were taken for each panel. PC, NC are positive and negative controls of *B. subtilis, B. clausii,* and *B. coagulans.*

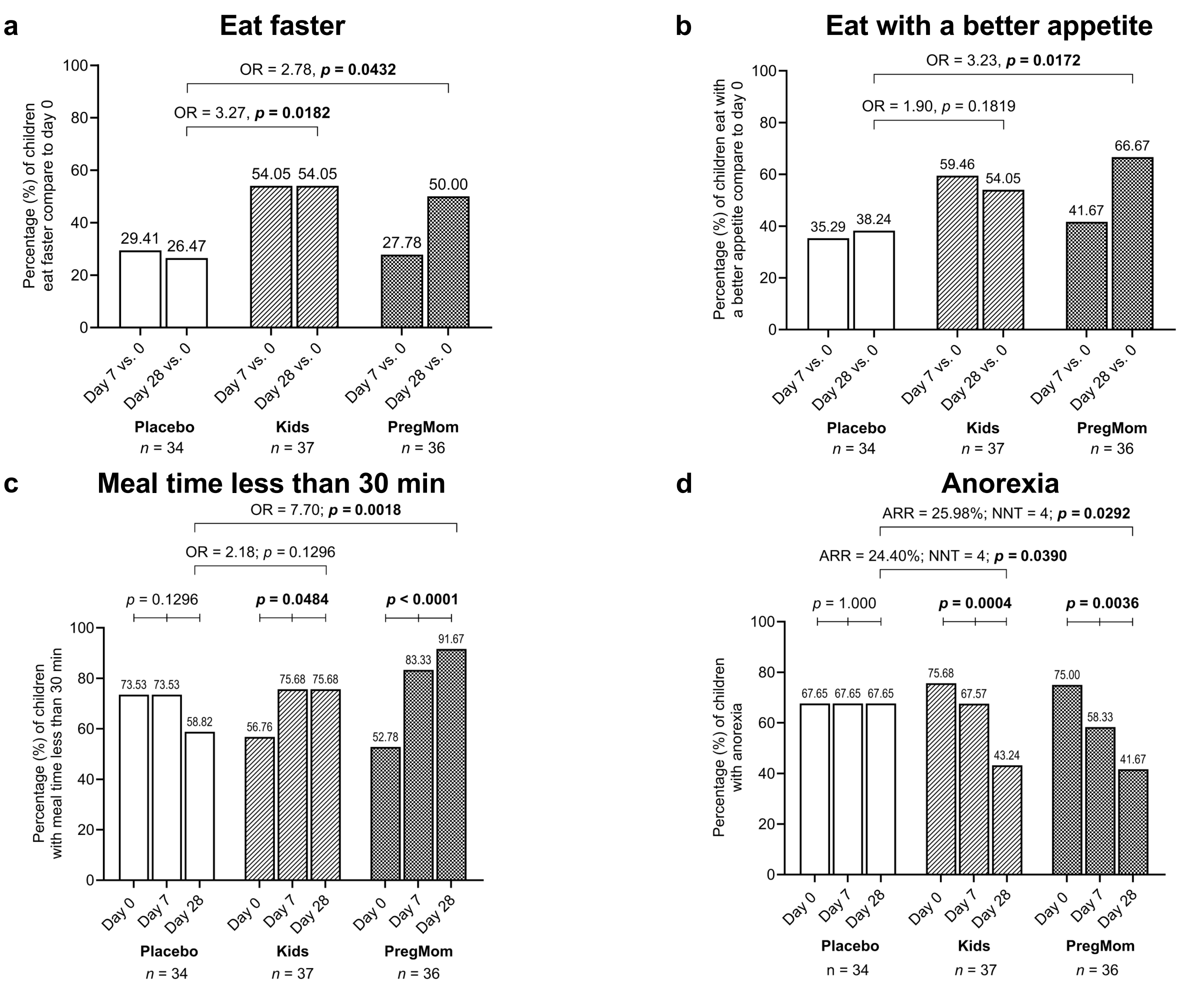

Supplementary Figure 8. Effects of probiotic interventions on children's appetite signs and overall reduction of anorexia.
Percentage of children showing signs associated with improved appetite among those diagnosed with anorexia (n = 34–37), including (a) eat faster, (b) eat with a better appetite, (c) meal time less than 30 min, and overall anorexia (d). Observations were made at days 0, 7, and 28 across the Control, Kids, and PregMom groups. The differences among time points within a group were assessed using the Mc Nemar test and Cochran Test, while the differences between two groups at the same time points were assessed using the chi-square test. The two-sided significance threshold was set at $p < 0.05$ and adjusted to $p < 0.0167$ and $p < 0.0083$ for within-group and between-group comparisons, respectively, to account for multiplicity across the three prespecified primary outcomes. Sample size: n = 34, 37, and 36 biologically independent participants for the Placebo, Kids, and PregMom groups, respectively.

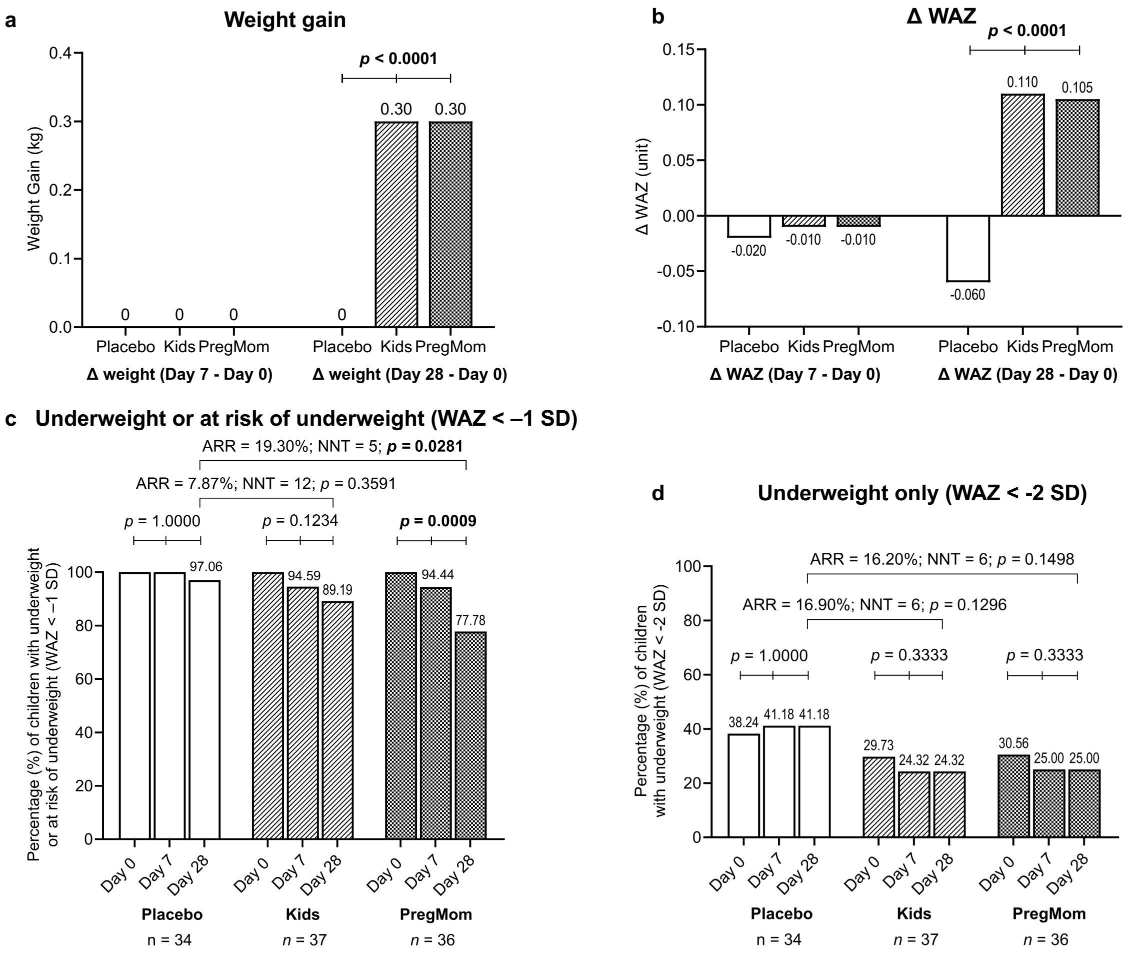

Supplementary Figure 9. Effects of probiotic interventions on weight gain, improved weight-for-age Z scores (WAZ), and reduced risk of underweight in children.

(**a**) Weight Gain, (**b**) Weight-for-Age Z-scores (WAZ), and (**c**) percentage of children with risk of underweight observed at days 0, 7, and 28 in the Control, Kids, and PregMom groups. The Kruskal-Wallis and ANOVA test were used to assess the differences among the three groups (**a, b**), while the Cochran's Q test was used to assess differences across three time points within a group (**c**). The Chi-square test was used to assess differences between two groups at the same time points (**c**). The two-sided significance threshold was set at $p < 0.05$ and adjusted to $p < 0.0167$ and $p < 0.0083$ for within-group and between-group comparisons, respectively, to account for multiplicity across the three prespecified primary outcomes. Sample size: $n = 34$, 37, and 36 biologically independent participants for the Placebo, Kids, and PregMom groups, respectively.

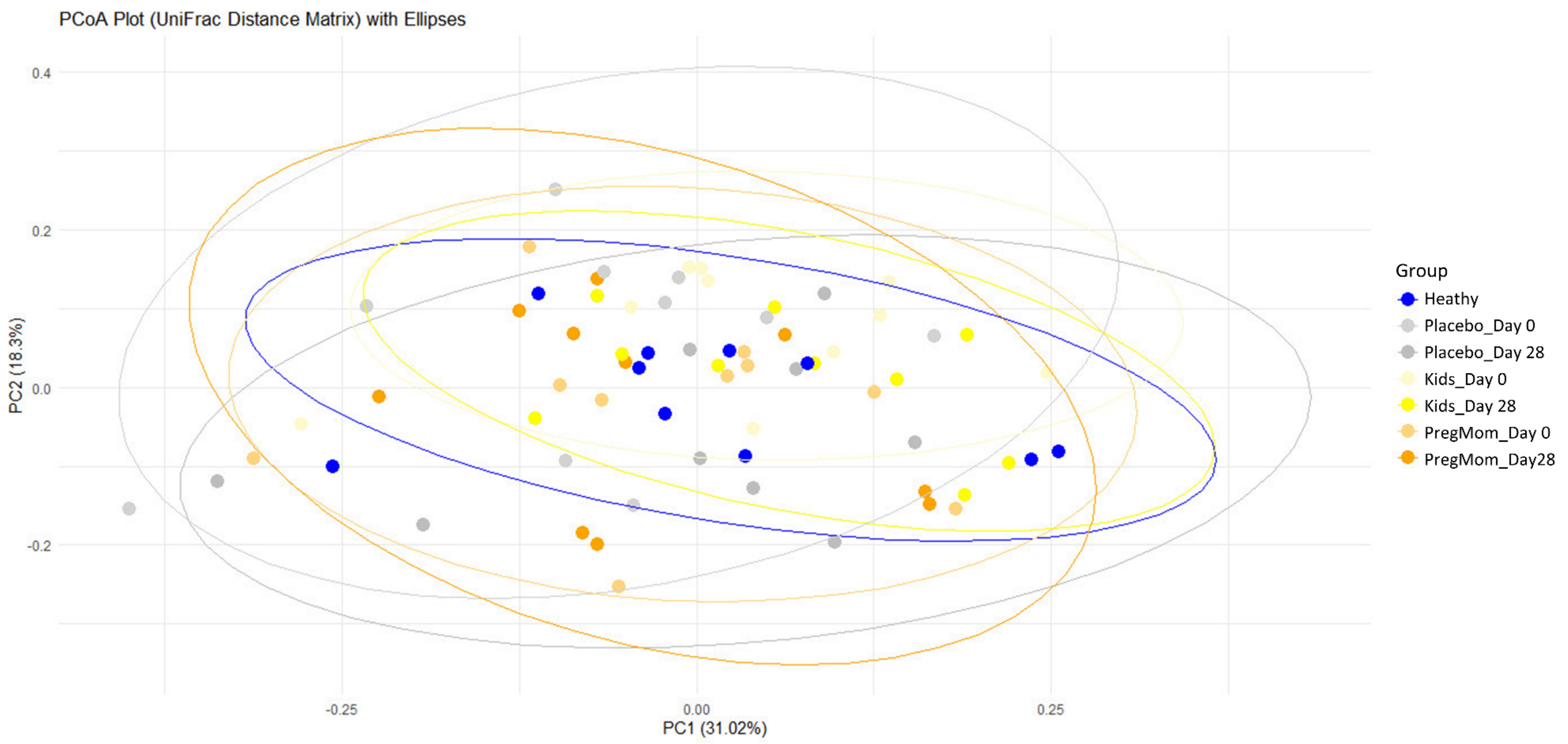

Supplementary Figure 10. PCoA analysis for beta diversity of the 16S rRNA gut microbiota were compared between the Placebo, Kids, and PregMom groups at day 28 vs. day 0, with the Healthy group serving as a reference.

II. Supplementary tables

Supplementary Table 1. Characterization of *Bacillus* strains used in LiveSpo Preg-Mom (*B. subtilis* ANA46, *B. clausii* ANA39, and *B. coagulans* ANA40) and LiveSpo Kids (*B. subtilis* ANA3 and *B. clausii* ANA39) formulations.

**Characteristics**	***B. subtilis* ANA3**	***B. subtilis* ANA46**	***B. clausii* ANA39**	***B. coagulans* ANA40**
Sporulation efficiency (%)	100	97	90	90
Heat stability of spores (°C)	80	80	65	60
Width size of vegetative cell (μm)	< 1 μm	< 1 μm	< 1 μm	< 1 μm
Amylase	+++	++	+++	++++
Caseinase	+++	+++	++++	-
Lipase	+	+	++	-
Catalase	+	+	+	+++
Gelatinase	+++	+++	+	++
Optimal temperature (°C)	37	37	35	44
Optimal pH	7.5	7.5	8.0	6.5
6.5% NaCl, 50°C	+	+	-	-
Aerobic/Anaerobic	++++	++++	+++	+++
Anerobic	++++	++++	+++	+++
Hemolysis	γ (No)	γ (No)	γ (No)	γ (No)
VP Test	+	+	-	+
Closest match*	*B. subtilis* (100%)	*B. subtilis* (100%)	*B. clausii* (99.5%)	*B. coagulans* (99.4%)

-, negative; +, weak or positive; ++, average; +++, good/high; ++++, very good/very high.

**Using 16S rDNA sequence analysis in this work. The similarity score is shown in brackets.*

Supplementary Table 2. Antibiotic susceptibility of *Bacillus Bacillus* strains used in LiveSpo Preg-Mom (*B. subtilis* ANA46, *B. clausii* ANA39, and *B. coagulans* ANA40) and LiveSpo Kids (*B. subtilis* ANA3 and *B. clausii* ANA39)

Antibiotic discs (µg)*	*B. subtilis* ANA3	*B. subtilis* ANA46	*B. clausii* ANA39	*B. coagulans* ANA40
Ampicillin (10)	26.34 ± 0.51 (S)	22.01 ± 0.4 (S)	27.04 ± 1.3 (S)	28.30 ± 0.21 (S)
Chloramphenicol (30)	25.41 ± 0.57 (S)	26.01 ± 0.5 (S)	18.47 ± 0.20 (S)	30.11 ± 0.39 (S)
Ciprofloxacin (5)	27.41 ± 0.28 (S)	26,11 ± 0.3 (S)	30.89 ± 0.61 (S)	25.10 ± 0.31 (S)
Clindamycin (2)	14.05 ± 0.24 (S)	20.15 ± 0.3 (S)	0 (R)	25.12 ± 1.08 (S)
Cotrimoxazole (25)	25.33 ± 0.45 (S)	28.60 ± 0.2 (S)	34.50 ± 0.95 (S)	25.71 ± 0.50 (S)
Erythromycin (15)	22.33 ± 0.11 (S)	20.56 ± 0.5 (S)	0 (R)	22.60 ± 0.51 (S)
Gentamicin (10)	21.72 ± 0.10 (S)	21.21 ± 0.39 (S)	28.22 ± 0.39 (S)	24.41 ± 0.31 (S)
Kanamycin (30)	24.18 ± 0.04 (S)	21.30 ± 0.1 (S)	24.84 ± 0.04 (S)	26.4 ± 0.03 (S)
Neomycin (30)	16.99 ± 0.24 (S)	17.44 ± 0.1 (S)	24.91 ± 0.13 (S)	21.40 ± 0.30 (S)
Rifampicin (30)	19.28 ± 0.57 (S)	25.10 ± 0.2 (S)	39.06 ± 0.68 (S)	39.87 ± 0.31 (S)
Streptomycin (10)	15.37 ± 0.33 (I)	13.45 ± 0.2 (I)	6.51 ± 0.46 (R)	16.20 ± 0.40 (S)
Tetracycline (30)	26.13 ± 0.25 (S)	15.10 ± 0.1 (I)	27.99 ± 0.14 (S)	35.50 ± 1.10 (S)
Trimethoprim (5)	0 (R)	30.0 6± 0.4 (S)	39.83 ± 0.72 (S)	22.81 ± 0.40 (S)
Vancomycin (30)	17.67 ± 0.00 (S)	16.13 ± 0.2 (S)	22.41 ± 0.22 (S)	20.41 ± 0.22 (S)
Azithromycin (15)	21.08 ± 0.23 (S)	19.69 ± 0.31 (S)	0 (R)	22.07 ± 0.48 (S)
Clarithromycin (15)	26.07 ± 0.25 (S)	24.88 0.02 (S)	0 (R)	24.49 ± 0.53 (S)

Notes:

**Antibiotic-impregnated discs (6 mm) with amount in µg shown in brackets.*

+*Diameter of inhibition zones from three individual experiments. S, sensitive; I, intermediate resistant; R, resistant.*

Supplementary Table 3. Sequence analysis of antibiotic resistance genes in *B. subtilis* ANA3 genome used in LiveSpo Kids

Resistance gene	% Identity	Query / Template length	Contig	Position in contig	Predicted phenotype	Accession number
addK	99.83	855/855	000002F |arrow	365509..366363	Aminoglycoside resistance	M26879
mph(K)	99.35	921/921	000001F |arrow	664415..665335	Macrolide resistance	NC_000964

Notes: The two genes, including *aadK* classified in the Aminoglycoside antibiotic group, and *mph(K)* classified in the Macrolide antibiotic group, may belong to acquired antibiotic resistance genes. The presence of the gene *aadK* is consistent with the streptomycin-resistant phenotype of *B. subtilis* ANA3, as indicated by the diffusion disc assay. Although *mph(K)* is present in the genome, its expression level may be low, which could explain why the strain remains sensitive to erythromycin. Interestingly, no resistance genes were found in the genome of *B. subtilis* ANA3, suggesting that the strain has intrinsic resistance to trimethoprim.

Supplementary Table 4. Sequence analysis of antibiotic resistance genes in *B. subtilis* ANA46 genome used in LiveSpo Preg-Mom

Resistance gene	% Identity	Query / Template length	Contig	Position in contig	Predicted phenotype	Accession number
addK	99.76	853/855	000000F |arrow	452124..452976	Aminoglycoside resistance	M26879
mph(K)	100	921/921	000000F |arrow	87052..97982	Macrolide resistance	NC_000964
tet(L)	96.79	1377/1377	000000F |arrow	37988..39364	Tetracycline resistance	D12567

Notes: The three genes including *addK* classified in Aminoglycoside antibiotic group, *mph(K)* classified in Macrolide antibiotic group, and *tet(L)* classified in Tetracycline antibiotic group were found. They may belong to acquired antibiotic resistant genes. The presence of the two genes *addK* and *tet(L)* are consistent with the streptomycin, and tetracycline resistant phenotype of *B. subtilis* ANA46 indicated by the diffusion disc assay. Although *mph(K)* is present in the genome, its expression level may be low, which could explain why the strain remains sensitive to erythromycin.

Supplementary Table 5. Sequence analysis of antibiotic resistance genes in *B. clausii* ANA39 genome used in LiveSpo Preg-Mom and LiveSpo Kids

Resistance gene	% Identity	Query / Template length	Contig	Position in contig	Predicted phenotype	Accession number
ant (4')- Ib	98.83	771 / 771	000000F |arrow	81510..82280	Aminoglycoside resistance	AJ506108
erm (34)	96.04	833 / 846	000000F |arrow	1033445..1034277	Macrolide resistance	AY234334
Cat	96.79	685 / 687	000000F |arrow	2589949..2590625	Phenicol resistance	AY238971

Notes: The three genes including ant(4')-Ib classified in Aminoglycoside antibiotic group, erm(34) classified in Macrolide antibiotic group, and cat classified in Phenicol antibiotic group were found. They may belong to acquired antibiotic resistant genes. The presence of the two genes ant (4')-Ib and erm (34) are consistent with the streptomycin and erythromycin resistant phenotype of *B. clausii* ANA39 indicated by the diffusion dics assay. Although cat is available in the genome, its expression level may be low so that the strain is still sensitive to chloramphenicol. Interestingly, clindamycin resistance gene was not found in the genome of *B. clausii* ANA39, suggesting that the strain is intrinsic resistance to clindamycin.

Supplementary Table 6. Sequence analysis of antibiotic resistance genes in *B. coagulans* ANA40 genome used in LiveSpo Preg-Mom

Resistance gene	% Identity	Query / Template length	Contig	Position in contig	Predicted phenotype	Accession number
None	-	-	-	-	-	-

Notes: Based on the antibiotic diffusion disc assay, and the results of analysis of the whole genome sequence of the *B. coagulans* ANA40, it can be concluded that the strain *B. coagulans* ANA40 does not exhibit antibiotic resistance activity, does not contain antibiotic resistance genes in both the DNA genome and plasmid, as well as does not contain any genes for intestinal toxins or food allergies.

Supplementary Table 7. Sequence analysis of toxin genes in *Bacillus* strains used in LiveSpo Preg-Mom (*B. subtilis* ANA46, *B. clausii* ANA39, and *B. coagulans* ANA40) and LiveSpo Kids (*B. subtilis* ANA3 and *B. clausii* ANA39)

No	Gene name	Detection method							
		Specific sequence amplified by PCR				***Number of gene detected by WGS***			
		B. subtilis ANA3	*B. subtilis* ANA46	*B. clausii* ANA39	*B. coagulans* ANA40	*B. subtilis* ANA3	*B. subtilis* ANA46	*B. clausii* ANA39	*B. coagulan* ANA40
1	Hemolysin B (*hbl*B)	ND	ND	ND	ND	0	0	0	0
2	Non-hemolytic enterotoxin A (*nhe*A)	ND	ND	ND	ND	0	0	0	0
3	*nhe*B	ND	ND	ND	ND	0	0	0	0
4	*nhe*C	ND	ND	ND	ND	0	0	0	0
5	Cytotoxin K (*cyt*K)	ND	ND	ND	ND	0	0	0	0

Notes: ND: Not detectable; PCR: Polymerase Chain Reaction; WGS: Whole Genome Sequencing

Supplementary Table 8. Nucleotide sequences of primers and probes for real time PCR TaqMan detection of *B. clausii; B. subtilis* and *B. coagulans*

***Bacillus* sp**	**Primer/Probe name**	**Sequence (5'-3')**	**Target gene**	**Length**	**Gene Bank**	**Concentration in reaction**
B. clausii	Fw-clausii	TTACCGCTCCTCAAGCAAA	erm	108	CP154609.1	500 nM
	Rw-clausii	CCATCGCGTCACAAACAATC				500 nM
	Probe - clausii	/5HEX/HEX-CCATCGCGT/ZEN/CACAAACAATC/3IABkFQ/				50 nM
B. subtilis	Fw-subtilis	GTATCCATCCAAAGCACACTTC	aprE	100	AB734701.1	500 nM
	Rv-subtilis	AAAGAATTAACGCTGCTGCTC				500 nM
	P-subtilis	56-FAM-CACTTACGG/ZEN/TGCTTACAACGGAACA/3IABkFQ/				50 nM
B. coagulans	Fw – coagulans	CCAATCAGCTACGGTTCGTTTA	AB434_2292	118	CP011939.1	500 nM
	Rv-coagulans	CCCTGTATCCCTGTTGTTTCAAA				500 nM
	P-coagulans	5Cy5-TATCCGACG/TAO/CTCCCGCATGCAATT/3IAbRQSp/				50 nM

Supplementary Table 9. Odds ratios, 95% confidence intervals, and *p* values for clinical constipation symptoms and eating behaviors at day 28 comparing LiveSpo Kids and LiveSpo PregMom with placebo.

**Outcome**	**OR**	**95% CI**	***p* value**
Number of bowel movements ≤ 2 times/week D28 Kids vs Placebo	3.491	1.116 to 10.05	0.0317
Number of bowel movements ≤ 2 times/week D28 PregMom vs Placebo	4.364	1.265 to 13.37	0.0226
Withholding defecation D28 Kids vs Placebo	3.111	1.141 to 8.544	0.0249
Withholding defecation D28 PregMom vs Placebo	2.6	0.9729 to 6.743	0.0563
Hard stools/anal pain D28 Kids vs Placebo	15.63	4.225 to 46.43	<0.0001
Hard stools/anal pain D28 PregMom vs Placebo	17.05	5.124 to 50.94	<0.0001
Large diameter stools D28 Kids vs Placebo	2.338	0.9295 to 6.401	0.0792
Large diameter stools D28 PregMom vs Placebo	3.293	1.238 to 8.655	0.017
Straining during defecation D28 Kids vs Placebo	2.344	0.9195 to 5.855	0.0805
Straining during defecation D28 PregMom vs Placebo	4.661	1.584 to 12.54	0.0035
Posture for stool retention D28 Kids vs Placebo	6.646	2.323 to 17.23	0.0002
Posture for stool retention D28 PregMom vs Placebo	11.37	3.342 to 32.88	<0.0001
Eat faster D28 Kids vs Placebo	3.268	1.228 to 8.855	0.0182
Eat faster D28 PregMom vs Placebo	2.778	1.028 to 7.561	0.0432
Eat with a better appetite D28 Kids vs Placebo	1.9	0.7643 to 5.055	0.1819
Eat with a better appetite D28 PregMom vs Placebo	3.231	1.247 to 8.102	0.0172
Meal time less than 30 min D28 Kids vs Placebo	2.178	0.7700 to 6.001	0.1296
Meal time less than 30 min D28 PregMom vs Placebo	7.7	2.021 to 26.88	0.0018

Notes: D28: day 28

Supplementary Table 10. Absolute Risk Reduction, Number Needed to Treat, 95% confidence intervals, and *p* values for clinical constipation symptoms and eating behaviors at day 28 comparing LiveSpo Kids and LiveSpo PregMom with placebo.

**Outcome**	**ARR (95% CI)**	**NNT (95% CI)**	***p* value**
Functional Constipation D28 Kids vs Placebo	0.5238 (0.3545 to 0.7726)	1.909 (1.294 to 2.821)	<0.0001
Functional Constipation D28 PregMom vs Placebo	0.5997 (0.4448 to 0.8468)	1.668 (1.181 to 2.248)	<0.0001
Anorexia D28 Kids vs Placebo	0.244 (0.03086 to 0.4944)	4.098 (2.023 to 32.40)	0.039
Anorexia D28 PregMom vs Placebo	0.2598 (0.04685 to 0.5125)	3.849 (1.951 to 21.35)	0.0292
Underweight or at risk of underweight (WAZ < –1 SD) D28 Kids vs Placebo	0.0787 (-0.0793 to 0.238)	12.7	0.3591
Underweight or at risk of underweight (WAZ < –1 SD) D28 PregMom vs Placebo	0.193 (0.0170 to 0.375)	5.19 (2.67 to 59.0)	0.0281
Underweight only (WAZ < -2 SD) D28 Kids vs Placebo	0.169 (-0.0475 to 0.404)	5.93	0.1296
Underweight only (WAZ < -2 SD) D28 PregMom vs Placebo	0.162 (-0.0560 to 0.400)	6.18	0.1498

Notes: D28: day 28
