# Supplementary material for: Bacillus spore probiotics for alleviating functional constipation in children: a randomized, double-blind, placebo-controlled trial
Source: Commun Med (Lond). 2026 Mar 18;6:148. doi: 10.1038/s43856-026-01517-6 (PMC13000159; doi:10.1038/s43856-026-01517-6)
Supplement: Supplementary file 2 — Description of Additional Supplementary files [file 43856_2026_1517_MOESM2_ESM.docx]

**Description of Additional Supplementary Files**

Supplementary Data 1 (PDF): Acute toxicity study in mice and sub-acute toxicity study in rabbits for LiveSpo Kids

Supplementary Data 2 (PDF): Acute toxicity study in mice and sub-acute toxicity study in rabbits for LiveSpo Preg-Mom.

Supplementary Data 3 – Source Data (XLSX): Numerical values underlying all main figures and tables.
