# Supplementary material for: Bacillus spore probiotics for alleviating functional constipation in children: a randomized, double-blind, placebo-controlled trial
Source: Commun Med (Lond). 2026 Mar 18;6:148. doi: 10.1038/s43856-026-01517-6 (PMC13000159; doi:10.1038/s43856-026-01517-6)
Supplement: Supplementary file 3 — Supplementary Data 1 [file 43856_2026_1517_MOESM3_ESM.pdf]

BẢN DỊCH - TRANSLATION

NATIONAL INSTITUTE OF DRUG QUALITY CONTROL

LABORATORY OF PHARMACOLOGY

Because LiveSpo KIDS and LiveSpo COLON share the identical composition—both containing *Bacillus subtilis* ANA3 and *Bacillus clausii* ANA39—this Toxicity Report applies equally to both products.

TEST RESULTS

ACUTE AND SUB-ACUTE TOXICITY

(Test results are valid for the test samples only)

GENERAL INFORMATION

Sample:	**LiveSpo® COLON/COLYTIS**
Producer:	LiveSpo Pharma Co. Ltd.,.
LOT:	8622C
Date of manufacture:	08/06/2022
Expiration Date:	07/06/2024
Sample sender:	LiveSpo Pharma Co. Ltd.,
Test criteria:	Acute and sub-acute toxicity
Refferences:	1. Methods for determining the toxicity of the drug - Medical Publisher, 2014<br>2. OECD guidelines for testing of chemicals. Repeated dose 28 - days Oral Toxicity study in Rodents OECD 407. 2008.
Formulations on the label:	Spores of *B. subtilis*, *B. clausii* 3 × 10^9 CFU in RO water just sufficient 5 mL.
Sample status when removed the seals for experiment:	The sample was packaged in a white ampoule, 5 mL and labeled. On the label, the name of the sample, place of manufacture, production batch, manufacturing date, and expiration date are included.
Place of testing:	Laboratory of Pharmacology – National Institute of Drug Quality Control
Address:	Tam Hiep, Thanh Tri, Hanoi, Vietnam
Headquarter:	48 Hai BaTrung, Hanoi, Vietnam

TEST RESULTS

1. Acute toxicity test

Implementation period: From 19/7/2022 to 02/8/2022

1.1. Experimental animals

- Species: Swiss white mice.
- Weight: 18 - 22 g.
- Quantity: 50 mice.
- Source of supply: National Institute of Hygiene and Epidemiology.
- Care conditions: Mice were caged in small groups of 6-8 individuals and placed in a temperature and humidity controlled room. Food and drinking water were supplied on demand. All manipulations on experimental animals were conducted in accordance with the procedures for care and use of experimental animals by the Laboratory of Pharmacology – National Institute of Drug Quality Control.

1.2. Experiment

Mice were fasted for 3-4 hours before entering the experiment; drinking water was supplied on demand. Mouse's weight was checked before the test. Mice that met the weight requirements were included in the test.

- Preparation of the test samples: Use the samples as provided
- Proportion of the sample: 0.9885 g/mL
- How to deliver the sample into the mice: Take the desired volume of sample and administer it into the mice's stomach with a soft curved feeding needle.

1.2.1. Preliminary test

- *Exploration of maximal tolerated dose:*

10 mice were administered 0.4 mL of test sample/mice for 3 times (2 hours apart) equivalent to the dose of 60 mL of test sample/kg mice. The test was qualified if, after 24 h of monitoring, no test mice were died and, after 7 days of monitoring, no test mice were died.

1.2.2. Real test

- *The dose levels of the test sample:*

Dose level 1: 20.0 mL test sample / kg mice (1 administration);

Dose level 2: 40.0 mL test sample / kg mice (2 administrations, 2 hours apart);

Dose level 3: 60.0 mL test sample / kg mice (3 administrations, 2 hours apart);

- *Control sample:* Water (3 administrations, 2 hours apart).

Procedures

Real tests were conducted on 40 mice, divided into 4 groups, including 1 control group and 3 test groups. The dose levels of test samples and the control sample were shown in Table 1.

Table 1. Acute toxicity test design

Group	Dose *(mL/kg mice)*	Dose *(mL/kg mice)*	No. of test mice
**Control** (C)	0.4 mL water x 3 times	----	10
**Test 1** (T1)	0.4 mL test sample x 1 time	20.0 mL/kg mice	10
**Test 2** (T2)	0.4 mL test sample x 2 times	40.0 mL/kg mice	10
**Test 3** (T3)	0.4 mL test sample x 3 times	60.0 mL/kg mice	10

Monitoring schedule

- Monitor the signs of poisoning: After suspension administered, the abnormal signs (in terms of physical condition, behavior, movement, eating, drinking, stool, urine, etc) were monitored every 15 min within the first hour and gradually reduce frequency within the first 24 h. Mice's activity was continued to be monitored once a day for a a total period of 7 test days.

- Monitor the number of dead mice among the test and control groups.

- Monitor the level of food and water consumption during the test.

- Monitor the weight at the following times: Just before administration; day 1, day 4, and day 7 after test sample administered in test groups compared to those of the control group (for the test groups with no dead mice were found).

1.3. Statistical analysis and data presentation

The data are presented as mean ± standard deviation (mean ± SD) and statistically analyzed using One-Way ANOVA combined with post-analysis Newman-Keuls test or statistically analyzed using Student test with Prism software version 8.0 (Graph Pad Software). P value < 0.05 was considered to be statistically significant.

1.4. Results

1.4.1. Consumption of food and water in mice

- The control group: Normal eating and drinking.

- The test groups: After administration with the test sample and during the 7 test days, no abnormalities were noticed. The level of food and water consumption was similar to that of the control group.

1.4.2. Observation of poisoning signs

- No sign of poisoning was observed in the test groups during the test period. Mice are healthy, agile, with smooth hair, and eat and move normally.

- No mice died during the test.

1.4.3. Mice's weight

- The results of mice weight monitoring in the control and test groups were shown in Tables 2.

Table 2. Results of mice weight monitoring

**Group (n = 10)**	**Weight of mice** *(g)*			
	**Before**	**After 1 day**	**After 4 days**	**After 7 days**
**Control**	19.32 ± 0.66	20.82 ± 0.75	25.64 ± 0.68	30.17 ± 1.16
**Test 1**	19.42 ± 0.93	20.93 ± 0.93	25.65 ± 1.14	30.36 ± 1.04
**Test 2**	19.44 ± 0.97	21.03 ± 0.91	25.76 ± 0.86	30.65 ± 1.10
**Test 3**	19.52 ± 0.84	21.06 ± 0.83	25.90 ± 0.81	30.57 ± 1.39

- The data on the weight comparison of mice between the test group and the control group were shown in Table 3.

Table 3. Comparison of the weight between the control and test groups

**Group**	**Before test**		**After test**		**Gained weight** *(%)*	$P_{before-after}$
	**Weight of mice** *(g)*	P_{before}	**Weight of mice** *(g)*	P_{after}		
**Control** (C)	19.32 ± 0.66	$P_{ANOVA} > 0.05$	30.17 ± 1.16	$P_{ANOVA} > 0.05$	156.2	$P < 0.001$
**Test 1** (T1)	19.42 ± 0.93	$P_{T1-C} > 0.05$	30.36 ± 1.04	$P_{T1-C} > 0.05$	156.5	$P < 0.001$
**Test 2** (T2)	19.44 ± 0.97	$P_{T2-C} > 0.05$	30.65 ± 1.10	$P_{T2-C} > 0.05$	157.8	$P < 0.001$

**Test 3** (T3)	19.52 ± 0.84	$P_{T3-C} > 0.05$	30.57 ± 1.39	$P_{T3-C} > 0.05$	156.7	$P < 0.001$

Comments:

The results of monitoring the average weight of mice during the period of the 7-day test showed that:

- Before an oral administration: The average weight of mice in the test groups before entering the test was not statistically significant difference in comparison with each other and comparison with the control group ($P_{ANOVA\ before} > 0.05$; $P_{(T\text{-}C)\ before} > 0.05$; $P_{(T\text{-}T)\ before} > 0.05$).

- After 7 days of oral administration: Mice in both control and test groups gained weight. There was a significant difference in the weight of mice at day 7 in compared to mice at day 0 in each group ($P_{before\text{-}after} < 0.001$). There was no statistically significant difference in average weight after test among the groups and compared to the control group ($P_{ANOVA\ after} > 0.05$; $P_{(T\text{-}C)\ after} > 0.05$; $P_{(T\text{-}T)\ after} > 0.05$).

1.4.4. Macroscopic observation results

At the end of the experiment, mice were dissected for macroscopic observation. The macroscopic observations showed that there was no difference in internal organs appearance (heart, liver, spleen, kidney, lung, etc) of the test group compared to the control group (Table 4).

Table 4. Results of macroscopic observation on mice

No.	Group	Macroscopic observations
1	Control	No abnormalities were observed
2	Test 1	There were no abnormalities compared to the control group
3	Test 2	There were no abnormalities compared to the control group
4	Test 3	There were no abnormalities compared to the control group

1.5. Conclusions

The results on the determination of acute toxicity on white mice of the sample **LiveSpo® COLON/COLYTIS** performed by National Institute of Drug Quality Control were as follows:

The test samples were administrated into mice at a dose level from 20.0 mL (12×10^{10} CFU *B. subtilis* and *B. clausii* spores)/ kg mice to 60.0 mL (36×10^{10} CFU *B. subtilis* and *B. clausii* spores)/ kg mice, no abnormalities were found in all test groups compared to that of

control group. Normal eating, drinking, and activities was observed and no dead mice were found.

The lethal dose for 50% of experimental animals (LD_{50}) was defined as greater than 60.0 mL test sample (equivalent to 59310 mg test sample/kg mice). The defined non-lethal dose in experimental animals (LD_0) was 60.0 mL test sample /kg mice. The dose that did not cause abnormal expression in the test animals was determined as 60.0 mL test sample/ kg mice. In the end of experiment, macroscopic observation of the internal organs of mice did not notice any abnormality among test groups compared to that of control group.

According to the toxicity classification of GHS (Globally Harmonized System of Classification and Labelling of Chemical,. 2019), the substances/ compounds with LD_{50} acute toxicity values greater than 5,000 mg/ kg mice intake orally in mice were considered to be low toxicity and unclassified. Based on the results obtained from this test, it can be concluded that the **LiveSpo®COLON/COLYTIS** sample has acute toxicity below the GHS classification threshold.

2. Assessment of the sub-acute toxicity

Implementation period: From 19/7/2022 to 30/9/2022.

2.1. Experimental animals

- Species: Mature and healthy New Zealand rabbit, both male and female. Female rabbits were not pregnant or lactating. The rabbits have never been subjected to any tests before. The rabbit's weights were in a range of 1.8 - 2.2 kg.

- Quantity: 21 rabbits were divided randomly into 3 groups (1 control group and 2 test groups), with 7 rabbits in each group.

- Source of supply: Department of Livestock – Laboratory of Pharmacology - National Institute of Drug Quality Control.

- Care conditions: Each rabbit was housed individually in a cage, placed in temperature and humidity controlled room, food and water were supplied on demand. All experimental manipulations on animals were conducted according to the procedures for care and use for experimental animals by the Laboratory of Pharmacology - Institute of National Drug Quality Control.

2.2. Procedures

2.2.1. Preparation of test sample

- Select the test dose level: Based on the maximum expected dose in human is 3 ampoules (equivalent to 15 mL test sample)/person/day and conversion ratio of dose between rabbit and human is 3.1, the two dose levels were selected, including:

+ The dose, equivalent to the expected dose for human: 0.93 mL of sample suspension/kg rabbit/day.

+ The dose, equivalent to 5-fold higher than the expected dose for human: 4.65 mL of sample suspension/kg rabbit/day.

- Preparation of samples:

+ *Control*: Water

+ *Suspension A (5-fold higher than the expected dose for human):* Use the original sample.

+ *Suspension B (equivalent to the expected dose for human):* Diluted the 20 mL suspension A in water just sufficient to 100 mL.

2.2.2. Experimental design

The experiment was conducted on 21 rabbits which were divided randomly into 3 groups, each group containing 7 rabbits. The experimental design with different dose levels was presented in Table 5.

Table 5. The test dose levels for sub-acute toxicity experiment

**Groups**	**Number of test rabbits**	**Volume for oral administration** *(mL/kg rabbit)*	**Dosage** *(mL sample/kg rabbit)*
Control	07	4.65 mL distilled water/kg rabbit	---
Test 1	07	4.65 mL suspension B/kg rabbit	0.93 mL/kg rabbit/ day
Test 2	07	4.65 mL suspension A/kg rabbit	4.65 mL/kg rabbit/ day

2.2.3. Monitoring and evaluation

- The rabbits were daily monitored for status of food and water consumption, physical condition, activity, stool, urine and other abnormal manifestations (if any).

- The weight of rabbits at day 0, 7, 14, 21, and 28 of administration and day 14 of follow-up since stopping administration were recorded.

- The hematological parameters related to hematopoietic function (number of red blood cells, white blood cells, platelets, hemoglobin, hematocrit); other parameters related to liver function (AST, ALT, total protein, total bilirubin, cholesterol) and kidney function (creatinine, urea) and glucose index at day 0, 14, 28 of administration and after 14 days since stopping administration were performed and recorded. The results of the test groups and the control group were statistically analyzed.

- At the end of experiment, the rabbits were dissected for macroscopic observation of internal organs appearance, such as: heart, liver, kidney, lung, stomach, and intestine of all rabbits.

- 03 rabbits in each group were randomly selected to perform histopathological specimens of liver, kidney have the microscopic evaluation of the organs immediately after stopping administration.

2.2.4. Statistical analysis and data presentation

The data were presented as mean ± standard deviation (mean ± SD) and statistically analyzed using Student test to compare the difference of the same indicator between the control and the test groups. P value < 0.05 was considered to be statistically significant.

2.3. Results

2.3.1. Rabbit status

During the test, all the rabbits moved normally, ate well, had bright eyes, dry feces, and smooth fur. Neither signs of abnormality in eating nor movement were found.

The rabbit weight monitoring during the test showed that:

- Before the experiment (before oral administration): The average weights of rabbits in the test groups were not different when compared to the control group *($P_{(T1\text{-}C)\ before} > 0.05$; $P_{(T2\text{-}C)\ before} > 0.05$).*

- After 28 days of oral administration: Rabbits gained weight steadily in both control and the two test groups. There was statistically difference in average weight at day 28 of experiment in comparison to that of the day before experiment in each group *($P_{before\text{-}after} < 0.01$)*. No statistically difference in the average weights between the test groups and the control group was found *($P_{(T1\text{-}C)\ after} > 0.05$; $P_{(T2\text{-}C)\ after} > 0.05$).*

- After 14 follow-up days since stopping administration: Rabbits were still healthy and gained weight well. No statistically difference in average weight between the test groups and the control group was found *($P_{(T1\text{-}C)\ after} > 0.05$; $P_{(T2\text{-}C)\ after} > 0.05$).*

Table 6. Rabbit's weight during sub-acute toxicity experiment

Group (n=7)	Weight (kg)					P
	Before the test (m_0)	*After 7 days (m_1)*	*After 14 days (m_2)*	*After 21 days (m_3)*	*After 28 days (m_4)*	
**Control (C)**	1.99 ± 0.13	2.13 ± 0.17	2.22 ± 0.05	2.31 ± 0.09	2.38 ± 0.10	$P_{before\text{-}after} < 0.01$

% Compared to before test		106.8 %	111.5 %	116.5 %	119.9 %	
**Test1 (T1)**	1.96 ± 0.13	2.10 ± 0.15	2.20 ± 0.15	2.28 ± 0.18	2.37 ± 0.21	$P_{before\text{-}after} < 0.001$<br>$P_{(T1\text{-}C)\ before} > 0.05$<br>$P_{(T1\text{-}C)\ after} > 0.05$
% Compared to before test		107.2 %	112.4 %	116.6 %	121.0 %	
**Test 2 (T2)**	1.99 ± 0.17	2.11 ± 0.17	2.21 ± 0.14	2.30 ± 0.15	2.39 ± 0.17	$P_{before\text{-}after} < 0.001$<br>$P_{(T2\text{-}C)\ before} > 0.05$<br>$P_{(T2\text{-}C)\ after} > 0.05$
% Compared to before test		105.9 %	111.2 %	116.1 %	120.2 %	

Table 7. The rabbit's weight after 14 follow-up days since stopped administration

**Group (n = 4)**	**Weight (kg)**	$P_{(T\text{-}C)}$ *after 14 follow-up days*
Control (C)	2.46 ± 0.09	
Test 1 (T1)	2.35 ± 0.06	*> 0.05*
Test 2 (T2)	2.41 ± 0.13	*> 0.05*

2.3.2. Hematological parameters related to hematopoietic function

a. Before the experiment (before oral administration):

Table 8. Hematological parameters before the experiment

**Index**	**Control (n = 7)**	**Group T1 (n = 7)**	$P_{(T1\text{-}C)}$	**Group T2 (n = 7)**	$P_{(T2\text{-}C)}$
**Red blood cell** *($x\ 10^{12}/l$)*	5.4 ± 0.5	5.2 ± 0.5	> 0.05	5.2 ± 0.4	> 0.05
**White blood cell** *($x\ 10^{9}/l$)*	6.9 ± 2.1	7.1 ± 1.4	> 0.05	6.7 ± 2.0	> 0.05

**Platelet** *(x 10^9/l)*	361.1 ± 130.6	332.6 ± 65.6	> 0.05	365.4 ± 116.5	> 0.05
**Hematocrit** *(%)*	36.7 ± 2.9	34.8 ± 2.9	> 0.05	35.6 ± 1.6	> 0.05
**Hemoglobin** *(g/dl)*	11.4 ± 1.0	10.9 ± 1.0	> 0.05	11.1 ± 0.6	> 0.05

b. In the middle (Day 14) of oral administration

Table 9. Hematological parameters at day 14 of oral administration

Index	Control (n = 7)	Group T1 (n = 7)	$P_{(T1-C)}$	Group T2 (n = 7)	$P_{(T2-C)}$
**Red blood cell** *(x 10^{12}/l)*	5.7 ± 0.5	5.3 ± 0,5	> 0.05	5.2 ± 0.4	> 0.05
**White blood cell** *(x 10^9/l)*	6.3 ± 2.0	7.6 ± 1,8	> 0.05	7.1 ± 2.1	> 0.05
**Platelet** *(x 10^9/l)*	325.7 ± 47.5	369.7 ± 83,0	> 0.05	369.9 ± 51.4	> 0.05
**Hematocrit** *(%)*	38.2 ± 2.6	36.7 ± 2,9	> 0.05	36.2 ± 2.2	> 0.05
**Hemoglobin** *(g/dl)*	12.0 ± 0.8	11.3 ± 0,9	> 0.05	11.2 ± 0.6	> 0.05

c. After (day 28) of oral administration

Table 10. Hematological parameters at day 28 of oral administration

Index	Control (n = 7)	Group T1 (n = 7)	$P_{(T1-C)}$	Group T2 (n = 7)	$P_{(T2-C)}$
**Red blood cell** *(x 10^{12}/l)*	5.6 ± 0.5	5.4 ± 0.4	> 0.05	5.4 ± 0.2	> 0.05
**White blood cell** *(x 10^9/l)*	9.0 ± 1.9	10.1 ± 1.6	> 0.05	8.6 ± 1.5	> 0.05
**Platelet** *(x 10^9/l)*	348.0 ± 123.2	364.7 ± 104.4	> 0.05	408.6 ± 138.6	> 0.05
**Hematocrit** *(%)*	37.8 ± 3.2	36.6 ± 2.7	> 0.05	37.1 ± 1.9	> 0.05
**Hemoglobin** *(g/dl)*	12.2 ± 0.8	11.6 ± 0.8	> 0.05	11.8 ± 0.6	> 0.05

d. After 14 follow-up days since stopped administration

Table 11. Hematological parameters after 14 follow-up days since stopped administration

Index	Group (n = 4)	Group T1 (n = 4)	*P* $_{(T1-C)}$	Group T2 (n = 4)	*P* $_{(T2-C)}$
**Red blood cell** *(x 10^{12}/l)*	5.7 ± 0.6	5.7 ± 0.3	> 0.05	5.7 ± 0.5	> 0.05
**White blood cell** *(x 10^9/l)*	9.3 ± 1.0	11.0 ± 2.2	> 0.05	11.5 ± 2.7	> 0.05
**Platelet** *(x 10^9/l)*	293.0 ± 65.5	370.8 ± 42.8	> 0.05	449.3 ± 157.8	> 0.05
**Hematocrit** *(%)*	38.1 ± 3.1	37.9 ± 1.7	> 0.05	38.0 ± 1.4	> 0.05
**Hemoglobin** *(g/dl)*	12.4 ± 0.8	12.3 ± 0.7	> 0.05	12.4 ± 0.6	> 0.05

Comments:

The results on hematological tests showed that:

- Before the experiment: There was no statically significant difference in hematological indicators between the control and the test groups (***P*** $_{(T-C)\ before}$ **> 0.05**).

- After 14 days, 28 days of oral administration and after 14 follow-up days since stopped administration, the hematological parameters were not statistically significant different between the test groups and the control group (***P*** $_{(T-C)\ day\ 14}$ > 0.05; ***P*** $_{(T-C)day\ 28}$ > 0.05). ***P*** $_{(T-C)\ after\ 14\ follow-up\ days}$ **> 0.05).**

2.3.3. Indicators related to liver function

a. Before the experiment (before oral administration):

Table 12. Parameters related to liver function before the experiment

Index	Control (n = 7)	Group T1 (n = 7)	*P* $_{(T1-C)}$	Group T2 (n = 7)	*P* $_{(T2-C)}$
**AST** *(U/l)*	40.2 ± 10.6	54.3 ± 22.9	> 0.05	50.8 ± 18.9	> 0.05
**ALT** *(U/l)*	66.8 ± 15.3	60.7 ± 20.0	> 0.05	66.6 ± 14.6	> 0.05
**Total Bilirubin** *(µmol/l)*	2.0 ± 0.9	2.0 ± 1.0	> 0.05	3.0 ± 1.5	> 0.05
**Total Protein** *(g/l)*	51.5 ± 4.6	49.3 ± 2.6	> 0.05	51.1 ± 4.3	> 0.05

**Cholesterol** *(mmol/l)*	34.7 ± 2.1	34.2 ± 1.1	> 0.05	33.8 ± 1.7	> 0.05

b.In the middle (day 14) of oral administration

Table 13. Parameters related to liver function at day 14 of oral administration

Index	Control (n = 7)	Group T1 (n = 7)	*P* $_{(T1-C)}$	Group T2 (n = 7)	*P* $_{(T2-C)}$
**AST** *(U/l)*	46.9 ± 16.3	60.4 ± 21.9	> 0.05	46.3 ± 15.1	> 0.05
**ALT** *(U/l)*	73.0± 24.4	68.4 ± 26.3	> 0.05	68.0 ± 20.8	> 0.05
**Total Bilirubin** *(μmol/l)*	1.9 ± 0.5	2.5 ± 0.8	> 0.05	1.6 ± 0.6	> 0.05
**Total Protein** *(g/l)*	53.5 ± 4.0	52.9 ± 3.9	> 0.05	52.8 ± 3.6	> 0.05
**Cholesterol** *(mmol/l)*	35.1 ± 1.8	35.5 ± 2.1	> 0.05	33.8 ± 1.2	> 0.05

c. After (day 28) of oral administration

Table 14. Parameters related to liver function at day 28 of oral administration

Index	Control (n = 7)	Group T1 (n = 7)	*P* $_{(T1-C)}$	Group T2 (n = 7)	*P* $_{(T2-C)}$
**AST** *(U/l)*	50.2 ± 20.8	41.7 ± 20.1	> 0.05	40.1 ± 8.1	> 0.05
**ALT** *(U/l)*	65.6 ± 14.4	59.4 ± 20.9	> 0.05	55.7 ± 23.2	> 0.05
**Total Bilirubin** *(μmol/l)*	2.0 ± 1,1	1.2 ± 0,6	> 0.05	1.5 ± 0.7	> 0.05
**Total Protein** *(g/l)*	55.2 ± 3.0	54.2 ± 2.9	> 0.05	56.2 ± 4.2	> 0.05
**Cholesterol** *(mmol/l)*	37.7 ± 2.3	37.1 ± 1.5	> 0.05	36.0 ± 1.3	> 0.05

d. After 14 follow-up days since stopped administration

Table 15. Parameters related to liver function after 14 follow-up days since stopped administration

Index	Control (n = 4)	Group T1 (n = 4)	$P_{(T1-C)}$	Group T2 (n = 4)	$P_{(T2-C)}$
**AST** *(U/l)*	31.3 ± 3.3	55.9 ± 22.3	> 0.05	42.0 ± 17.0	> 0.05
**ALT** *(U/l)*	76.7 ± 9.7	96.2 ± 20.7	> 0.05	59.3 ± 19.2	> 0.05
**Total Bilirubin** *(µmol/l)*	1.8 ± 0.4	2.7 ± 1.0	> 0.05	1.3 ± 0.4	> 0.05
**Total Protein** *(g/l)*	57.4 ± 2.8	58.2 ± 1.2	> 0.05	60.3 ± 4.6	> 0.05
**Cholesterol** *(mmol/l)*	35.7 ± 1.2	37.2 ± 1.1	> 0.05	35.7 ± 0.6	> 0.05

Comments:

The results on liver function indicators showed that:

- Before the experiment: There was no statistically significant difference in the indicators related to liver function between the control group and the test groups ($P_{(T-C)\ before} > 0.05$).

- After 14 days, 28 days of oral administration and after 14 follow-up days since stopping administration: There were no statistically significant difference in liver function related indices between the control and the test groups. ($P_{(T-C)\ day\ 14} > 0.05$; $P_{(T-C)\ day\ 28} > 0.05$).$P_{(T-C)\ after\ 14\ follow-up\ days} > 0.05$).

2.3.4. Parameters related to kidney function

a. Before the experiment (before oral administration):

Table 16. Parameters related to kidney function before the experiment

Index	Control (n = 7)	Group T1 (n = 7)	$P_{(T1-C)}$	Group T2 (n = 7)	$P_{(T2-C)}$
**Urea** *(mmol/l)*	4.9 ± 1.4	4.3 ± 0.8	> 0.05	4.0 ± 1.4	> 0.05
**Creatinin** *(µmol/l)*	105.3 ± 23.0	96.0 ± 14.5	> 0.05	85.2 ± 12.0	> 0.05

b. In the middle (day 14) of oral administration

Table 17. Parameters related to kidney function at day 14 of oral administration

Index	Control (n = 7)	Group T1 (n = 7)	$P_{(T1-C)}$	Group T2 (n = 7)	$P_{(T2-C)}$
**Urea** *(mmol/l)*	5.2 ± 0.9	4.6 ± 1.2	> 0.05	4.5 ± 0.5	> 0.05
**Creatinin** *(µmol/l)*	111.9 ± 18.4	114.4 ± 19.8	> 0.05	96.9 ± 8.7	> 0.05

c. After (day 28) of oral administration

Table 18. Parameters related to kidney function at day 28 of oral administrations

Index	Control (n = 7)	Group T1 (n = 7)	$P_{(T1-C)}$	Group T2 (n = 7)	$P_{(T2-C)}$
**Urea** *(mmol/l)*	5.3 ± 0.9	5.2 ± 1.2	> 0.05	4.6 ± 0.8	> 0.05
**Creatinin** *(µmol/l)*	110.9 ± 8.5	105.6 ± 11.7	> 0.05	104.6 ± 4.3	> 0.05

d. After 14 follow-up days since stopped administration

Table 19. Parameters related to kidney function after 14 follow-up days since stopped administration

Index	Control (n = 4)	Group T1 (n = 4)	$P_{(T1-C)}$	Group T2 (n = 4)	$P_{(T2-C)}$
**Urea** *(mmol/l)*	5.8 ± 1.0	6.0 ± 0.8	> 0.05	5.1 ± 0.7	> 0.05
**Creatinin** *(µmol/l)*	129.2 ± 13.8	131.4 ± 15.2	> 0.05	112.7 ± 14.8	> 0.05

Comments:

Results on urea and creatinine tests showed that:

- Before the experiment: There was no statistically significant difference in the parameters related to kidney function between the control and the 2 test groups ($\boldsymbol{P_{(T-C)\ before} > 0.05}$).

- After 14 days, 28 days of oral administration and after 14 follow-up days since stopping administration: There was no statistically significant difference in the parameters related to kidney function between the control and the test groups ($P_{(T-C)\ day\ 14} > 0.05$; $P_{(T-C)\ day\ 28} > 0.05$); $P_{(T-C)\ after\ 14\ follow\text{-}up\ days} > 0.05$).

2.3.5. Blood glucose index monitoring results

a. Before the experiment (before oral administration):

Table 20. Glucose index before the experiment

Index	Control (n = 4)	Group T1 (n = 4)	$P_{(T1-C)}$	Group T2 (n = 4)	$P_{(T2-C)}$
Glucose *(mmol/l)*	6.4 ± 1.6	7.1 ± 1.0	> 0.05	7.2 ± 0.8	> 0.05

b. In the middle (day 14) of oral administrations

Table 21. Glucose index at day 14 of oral administration

Index	Control (n = 4)	Group T1 (n = 4)	$P_{(T1-C)}$	Group T2 (n = 4)	$P_{(T2-C)}$
Glucose *(mmol/l)*	6.7 ± 0.9	6.3 ± 0.4	> 0.05	6.4 ± 0.8	> 0.05

c. After (day 28) of oral administrations

Table 22. Glucose index at day 28 of oral administrations

Index	Control (n = 7)	Group T1 (n = 7)	$P_{(T1-C)}$	Group T2 (n = 7)	$P_{(T2-C)}$
Glucose *(mmol/l)*	6.6 ± 0.8	6.8 ± 0.7	> 0.05	6.6 ± 0.5	> 0.05

d. After 14 follow-up days since stopped administration

Table 23. Glucose index after 14 follow-up days since stopped administration

Index	Control (n = 4)	Group T1 (n = 4)	$P_{(T1-C)}$	Group T2 (n = 4)	$P_{(T2-C)}$
Glucose *(mmol/l)*	6.3 ± 1.0	5.8 ± 0.6	> 0.05	5.4 ± 0.3	> 0.05

Comments

The data on the blood glucose test showed that:

- Before the experiment: There was no statistically significant difference in glucose index between the control group and the 2 test groups ($P_{(T-C)\ before} > 0.05$).

- After 14 days, 28 days of oral administration and after 14 follow-up days since stopping administration: There was no statistically significant difference in glucose index between the control and the 2 test groups ($P_{(T-C)\ day\ 14} > 0.05$; $P_{(T-C)\ day\ 28} > 0.05$); $P_{(T-C)\ after\ 14\ follow\text{-}up\ days} > 0.05$).

2.3.6. Macroscopic observation

The results of macroscopic observation for internal organs appearance showed that, after the experiment, there were no abnormal appearances in color of heart, lung, liver, spleen, kidney, stomach, intestines of rabbits in test groups compared to control group.

Table 24. Macroscopic images of internal organs

**Macroscopic images**		
Control group	***Test 1 (Low dose)***	***Test 2 (high dose)***
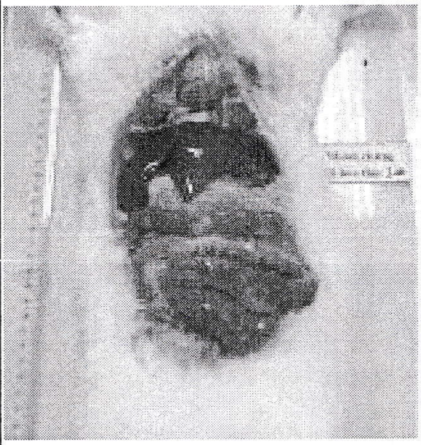	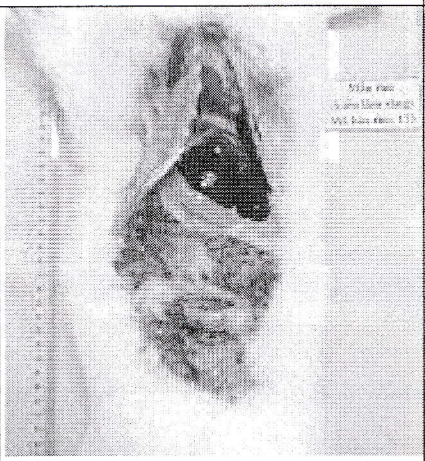	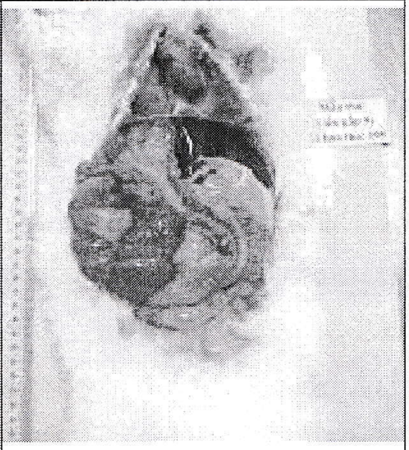

2.3.7. Microscopic observation

Liver, kidney specimens were fixed with 10% formalin, stained with Hematoxylin Eosin (HE) and Perioric Acid Shiff (PAS) staining solution and observed under optical microscope.

The microscopic observation was performed at the Department of Pathophysiology Anatomy - Hanoi Medical University. The results showed that the entire test rabbits showed unscathed liver, kidney. Morphological anatomy was within normal limits. There were no abnormal symptoms related to the test groups using the two different dose levels compared with the control group.

The detailed microscopic analysis was as follows:

Liver:

- Hepatocytes: No injury.
- Liver capillaries: Normal.
- Central vein: Normal.
- Portal hepatic: Normal.
- Interstitial tissue: Normal.

Conclusion: Liver tissue morphology was normal, there was no apparent injury

Kidney:

- Glomerulus (capillary, mesangium, Baomann capsule): Normal.
- Hepatic duct/ Renal columns: Normal.
- Renal pelvis: Normal
- Interstitial tissue: Normal.

Conclusion: Kidney tissue is normal and there was no apparent injury

Small intestine:

- Intestinal mucosa (villi, absorptive cells, goblet cells, stroma, mucosal muscle): Normal.
- Submucosa: Normal.
- Muscle layer: Normal
- Intestinal serosa: Normal.

Conclusion: Small intestinal tissue is normal and there was no apparent injury

Large intestine:

- Intestinal mucosa (single columnar epithelium, goblet cells, Lieberkuhn glands, stroma, mucosal muscle): Normal.
- Submucosa: Normal.
- Muscle layer: Normal
- Intestinal serosa: Normal.

Conclusion: Large intestinal tissue is normal and there was no apparent injury

Table 25. The histopathological anatomy images under microscope of liver, kidney, small and large intestines

(HE stain x400)

No	*Group*	*Liver tissue*	*Kidney tissue*	*Small intestine*	*Large intestine*
1	***Control***	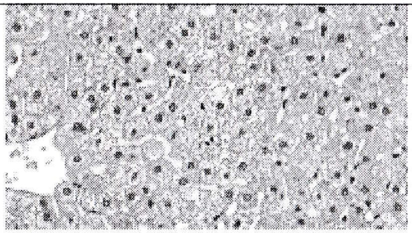 Normal and unscathed liver tissue morphology.	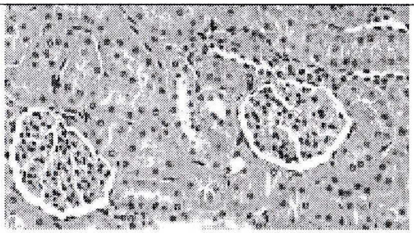 Normal and unscathed kidney tissue morphology.	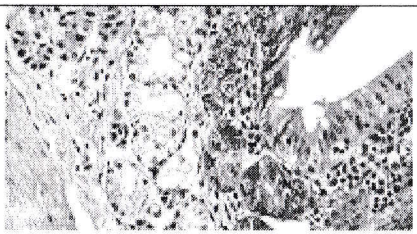 Normal and unscathed small intestine tissue morphology.	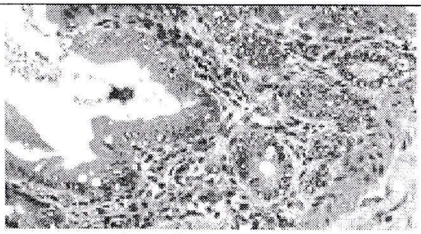 Normal and unscathed large intestine tissue morphology.
2	***Test 1 (low dose)***	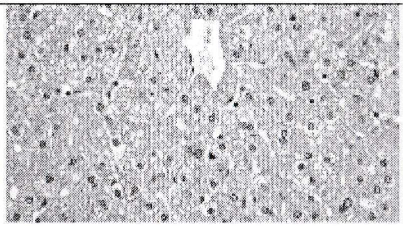 Normal and unscathed liver tissue morphology.	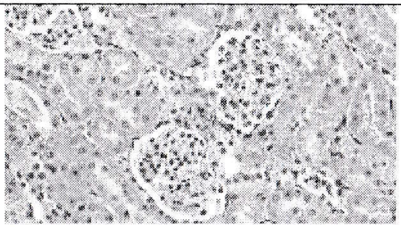 Normal and unscathed kidney tissue morphology.	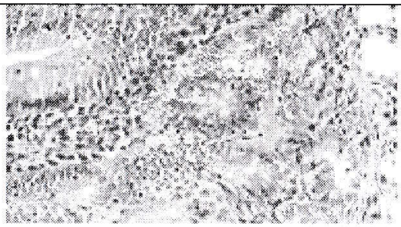 Normal and unscathed small intestine tissue morphology.	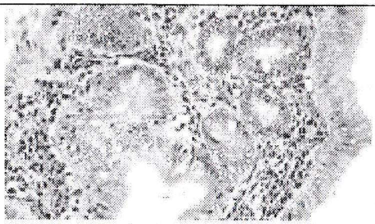 Normal and unscathed large intestine tissue morphology.
3	***Test 2 (high dose)***	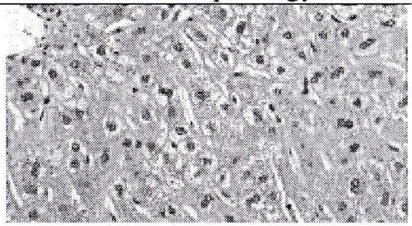 Normal and unscathed liver tissue morphology.	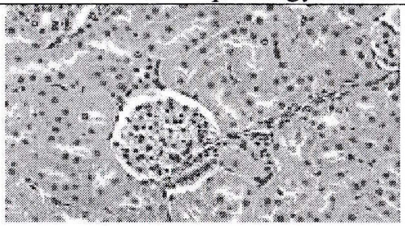 Normal and unscathed kidney tissue morphology	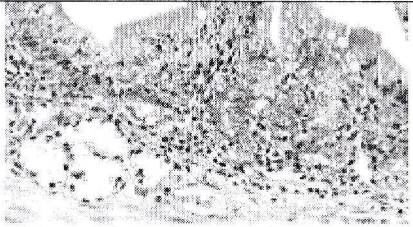 Normal and unscathed small intestine tissue morphology.	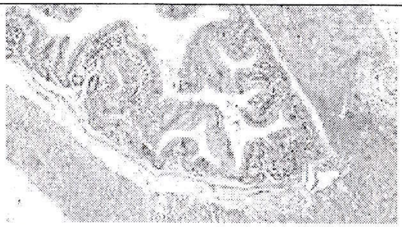 Normal and unscathed large intestine tissue morphology.

Table 26. The histopathological anatomy images under microscope of liver, kidney, small and large intestine

(PAS stain x400)

No	*Group*	*Liver tissue*	*Kidney tissue*	*Small intestine*	*Large intestine*
1	***Control***	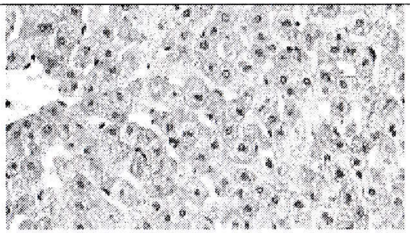 Normal and unscathed liver tissue morphology.	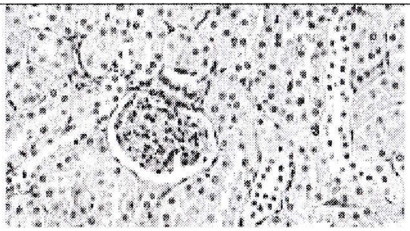 Normal and unscathed kidney tissue morphology.	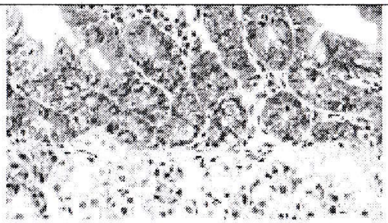 Normal and unscathed small intestine tissue morphology	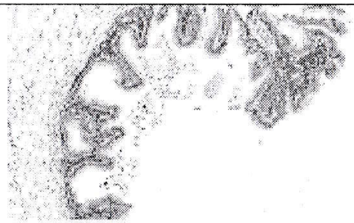 Normal and unscathed large intestine tissue morphology.
2	***Test 1 (low dose)***	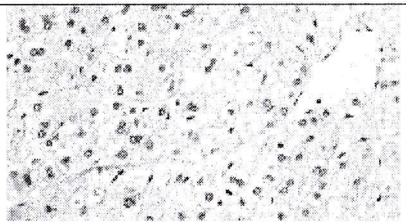 Normal and unscathed liver tissue morphology	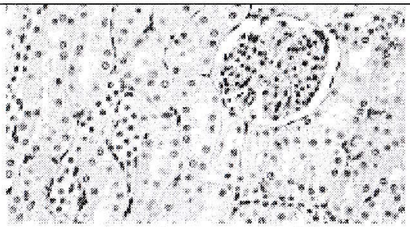 Normal and unscathed kidney tissue morphology	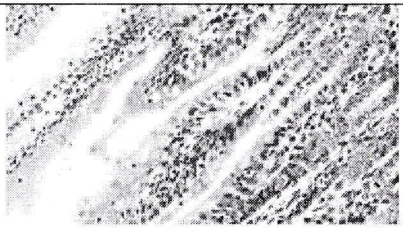 Normal and unscathed small intestine tissue morphology	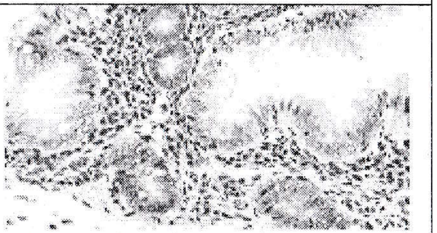 Normal and unscathed large intestine tissue morphology.
3	***Test 2 (high dose)***	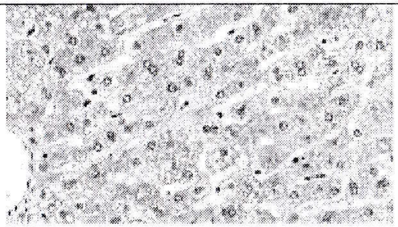 Normal and unscathed liver tissue morphology	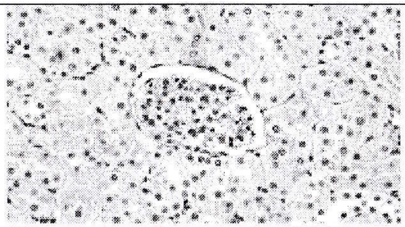 Normal and unscathed kidney tissue morphology	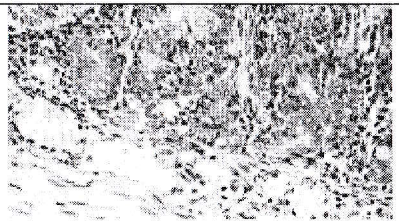 Normal and unscathed small intestine tissue morphology	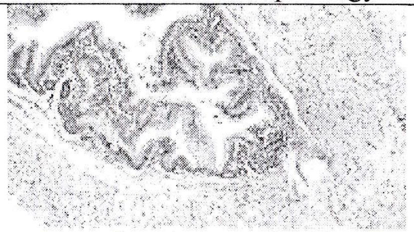 Normal and unscathed large intestine tissue morphology.

2.4. Conclusions

The results on sub-acute toxicity on rabbits of the sample **LiveSpo COLON/COLYTIS** that performed by National Institute of Drugs Quality Control are as follows:

After 28 days of continuous oral administration of test suspension at the two different doses of 0.93 mL/kg rabbit/day (equivalent to the maximum expected human dose of 3 ampoules/person/day or 9×10^9 CFU *B. subtilis* and *B. clausii* spores/person/day) and 4.65 mL/kg rabbit/day (5-fold higher than the maximum human dose, equivalent to 4.5×10^{10} CFU *B. subtilis* and *B. clausii* spores/person/day), the weight, physical condition, and movement of the test rabbits were not affected. The rabbits were healthy and gained weight.

The biochemical indicators for liver and kidney functions (activities of enzymes AST, ALT, total protein, total bilirubin, cholesterol, glucose, urea, creatinine) and hematological parameters (red blood cells, hemoglobin, hematocrit, leukocytes, platelets) were not statistically significant different at the points of before the experimental, day 14, day 28 of the experiment and after 14 follow-up days since stopping administration in comparison between the two test groups and the control group.

Under macroscopic observation, abnormalities were not found in the heart, lung, liver, spleen, kidney, stomach, and intestines of the test rabbits. Microscopic observation did not show histopathological lesions of liver or kidney between the two test groups and the control group.

Ha Noi, Sep 26th 2022

Certified by the Head of Organization	LABORATORY OF PHARMACOLOGY
(signed and stamped)	(Signed)
Doan Cao Son	Dr. Nguyen Thi Lien

LỜI CHỨNG CỦA CÔNG CHỨNG VIÊN
TESTIMONY OF NOTARY PUBLIC

Hôm nay, ngày 05 tháng 10 năm 2022 (Ngày mồng năm tháng mười, năm hai nghìn không trăm hai mươi hai)

Today, October 05, 2022

Tại Văn phòng Công chứng Trương Thị Nga địa chỉ tại A4 - TT19 Khu đô thị Văn Quán, Yên Phúc, phường Phúc La, quận Hà Đông, thành phố Hà Nội

At Truong Thi Nga Notary Public Office, A4 – TT19 Van Quan Urban Area, Yen Phuc, Phuc La Ward, Ha Dong District, Hanoi City,

Tôi, công chứng viên, trong phạm vi trách nhiệm của mình theo quy định của pháp luật,

I, the undersigned Notary Public, within the scope of my authority as stipulated by law,

CHỨNG NHẬN:

DOES HEREBY CERTIFY THAT:

- Bản dịch này do bà Phạm Thị Tuyết Mai cộng tác viên phiên dịch của Văn phòng Công chứng Trương Thị Nga A4 - TT19 Khu đô thị Văn Quán, Yên Phúc, phường Phúc La, quận Hà Đông, thành phố Hà Nội, dịch từ **tiếng Việt sang tiếng Anh;**

*This translation is translated from **Vietnamese** into **English** by Ms Pham Thi Tuyet Mai, the freelance translator of Truong Thi Nga Notary Public Office, A4 – TT19 Van Quan Urban Area, Yen Phuc, Phuc La Ward, Ha Dong District, Hanoi City;*

- Chữ ký trong bản dịch đúng là chữ ký của bà Phạm Thị Tuyết Mai;

The signature in the translation is exactly Ms Pham Thi Tuyet Mai's;

- Nội dung bản dịch chính xác, không vi phạm pháp luật, không trái đạo đức xã hội;

The content of the translation is correct and is not contrary to the legal provisions and social morality;

- Văn bản công chứng này được lập thành 02(hai) bản chính, mỗi bản gồm .... tờ, ... trang , lưu 01 bản tại Văn phòng Công chứng Trương Thị Nga, A4 - TT19 Khu đô thị Văn Quán, Yên Phúc, phường Phúc La, quận Hà Đông, thành phố Hà Nội

The notarized translation is made into 02 (two).originals, each of which includes …….. sheets, …….. pages; one of them is recorded at Truong Thi Nga Notary Public Office, A4 – TT19 Van Quan Urban Area, Yen Phuc, Phuc La Ward, Ha Dong District, Hanoi City.

Số công chứng 4801, quyển số .01/2022TP/CC-SCC/BD
Certification No.: …….. Book No 01/2022TP/CC-SCC/BD

Người dịch	**CÔNG CHỨNG VIÊN**
**Translator**	***NOTARY PUBLIC***
Mai	S.Đ.K.H.Đ : 113 - C.T.H.D ★ VĂN PHÒNG CÔNG CHỨNG TRƯƠNG THỊ NGA ★ Q. HÀ ĐÔNG - T.P HÀ NỘI
**Phạm Thị Tuyết Mai**	CÔNG CHỨNG VIÊN<br>Lại Hồng Khánh

VIỆN KIỂM NGHIỆM THUỐC TRUNG ƯƠNG

KHOA DƯỢC LÝ

KẾT QUẢ THỬ NGHIỆM
ĐỘC TÍNH CẤP VÀ BÁN TRƯỜNG DIỄN

(Kết quả thử nghiệm chỉ có giá trị với mẫu đem thử)

THÔNG TIN CHUNG

Tên mẫu:	**LiveSpo COLON/COLYTIS**
Nhà sản xuất:	Công ty TNHH LiveSpo Pharma
Lô sản xuất:	8622C
Ngày sản xuất:	08/06/2022
Hạn dùng:	07/06/2024
Nơi gửi mẫu:	Công ty TNHH LiveSpo Pharma
Chỉ tiêu thử:	Độc tính cấp và Bán trường diễn
Tài liệu thử:	1. Phương pháp xác định độc tính của thuốc – Nhà xuất bản Y học 2014<br>2. OECD guidelines for testing of chemicals. Repeated dose 28 - days Oral Toxicity study in Rodents OECD 407, 2008.
Công thức bào chế trên nhãn:	*B. subtilis và B. clausii* nồng độ 3 tỷ CFU, nước cất vừa đủ 5 ml
Tình trạng mẫu khi mở niêm phong để thử nghiệm:	Mẫu thử được đóng trong ống nhựa màu trắng, mỗi ống 5 ml, bên ngoài ống có nhãn. Trên nhãn ghi tên mẫu, nơi sản xuất, lô sản xuất, ngày sản xuất, hạn dùng.
Nơi thực hiện:	Khoa Dược lý - Viện Kiểm nghiệm Thuốc Trung Ương
Địa chỉ	Tam Hiệp, Thanh Trì, Hà Nội
Trụ sở	48 Hai Bà Trưng, Hà Nội

KẾT QUẢ THỬ NGHIỆM

1. Thử nghiệm độc tính cấp

Thời gian thực hiện: 19/07/2022 đến ngày 02/08/2022

1.1. Động vật thí nghiệm

- Loài: chuột nhắt trắng giống Swiss.

- Cân nặng: 18 - 22 g.

- Số lượng: 50 con.

- Nguồn gốc cung cấp: Viện Vệ sinh Dịch tễ Trung ương.

- Điều kiện chăm sóc: Chuột được nuôi 6 - 8 con một chuồng trong phòng nuôi có kiểm soát nhiệt độ và độ ẩm thích hợp với thức ăn và nước uống theo nhu cầu. Tất cả các thao tác trên động vật thí nghiệm đều được tuân theo các quy trình về chăm sóc và sử dụng động vật thí nghiệm của Khoa Dược lý - Viện Kiểm nghiệm thuốc TW.

1.2. Thực nghiệm

- Chuột được nhịn ăn 3 - 4 giờ trước khi thử nghiệm, nước uống theo nhu cầu. Kiểm tra cân nặng trước khi thử nghiệm. Chuột đạt các yêu cầu về cân nặng được đưa vào thử nghiệm.

- Cách xử lý và chuẩn bị mẫu thử: Dùng nguyên mẫu.

- Tỷ trọng của mẫu thử là 0,9885 g/ml

- Cách cho chuột uống: Lấy thể tích mẫu thử theo quy định đưa thẳng vào dạ dày chuột bằng kim cong đầu tù.

1.2.1. Thử sơ bộ

- Thăm dò ở mức liều dung nạp tối đa:

Dùng 10 chuột, cho mỗi chuột uống 0,4 ml mẫu thử x 3 lần (mỗi lần cách nhau 2 giờ) tương đương mức liều 60 ml mẫu thử/kg chuột. Sau 24 giờ theo dõi, không có chuột thí nghiệm bị chết. Theo dõi 7 ngày, không có chuột thí nghiệm bị chết.

1.2.2. Thử nghiệm chính thức

- Các mức liều của mẫu thử:

Mức liều 1: 20,0 ml mẫu thử/kg chuột (1 lần uống);

Mức liều 2: 40,0 ml mẫu thử/kg chuột (2 lần uống, mỗi lần uống cách nhau 2 giờ);

Mức liều 3: 60,0 ml mẫu thử/kg chuột (3 lần uống, mỗi lần uống cách nhau 2 giờ);

- Mẫu chứng: Nước (3 lần uống, mỗi lần uống cách nhau 2 giờ).

Tiến hành

Tiến hành thử nghiệm chính thức trên 40 chuột, chia thành 4 nhóm gồm 1 nhóm chứng và 3 nhóm thử. Các nhóm chuột được dùng mẫu thử và mẫu chứng theo mức liều được trình bày ở Bảng 1.

Bảng 1. Bố trí thử nghiệm thử độc tính cấp

Nhóm	Liều dùng *(ml/chuột)*	Liều dùng *(ml mẫu thử/kg chuột)*	Số chuột thí nghiệm
**Chứng** (C)	0,4 ml nước x 3 lần	----	10
**Thử 1** (T1)	0,4 ml mẫu thử x 1 lần	20,0 ml/kg chuột	10
**Thử 2** (T2)	0,4 ml mẫu thử x 2 lần	40,0 ml/kg chuột	10
**Thử 3** (T3)	0,4 ml mẫu thử x 3 lần	60,0 ml/kg chuột	10

Lịch theo dõi

- Theo dõi biểu hiện ngộ độc: Sau khi uống hỗn dịch thử theo dõi các dấu hiệu bất thường (về thể trạng, hành vi, vận động, tình trạng ăn, uống, phân, nước tiểu...) với tần suất khoảng 15 phút 1 lần trong vòng 1 giờ đầu và giãn dần tần suất trong vòng 24 giờ đầu. Tiếp tục theo dõi hoạt động của động vật thí nghiệm mỗi ngày 1 lần trong thời gian 7 ngày sau khi uống.

- Theo dõi số chuột chết trong các nhóm thử và nhóm chứng.

- Theo dõi mức độ tiêu thụ thức ăn, nước uống trong thời gian thử nghiệm.

- Theo dõi khối lượng chuột tại các thời điểm ngay trước khi uống, 1 ngày, 4 ngày và 7 ngày sau khi uống mẫu thử so với nhóm chứng (với các nhóm thử không có chuột thí nghiệm bị chết).

1.3. Trình bày và xử lý số liệu

Số liệu được trình bày dưới dạng giá trị trung bình cộng trừ độ lệch chuẩn (mean ± SD) và được xử lý thống kê bằng phép phân tích biến 1 chiều (one-way ANOVA) với hậu kiểm (post-hoc) Newman-Keuls test hoặc được xử lý thống kê bằng trắc nghiệm Student sử dụng phần mềm Prism phiên bản 8.0 (Graph Pad Software). Giá trị $P < 0,05$ được coi là có ý nghĩa thống kê.

1.4. Kết quả

1.4.1. Tiêu thụ thức ăn và nước uống của chuột

- Nhóm chứng: Ăn uống bình thường.

- Các nhóm thử: Sau khi uống thuốc và trong 7 ngày theo dõi nhóm thử, không nhận thấy có biểu hiện gì khác thường. Mức độ tiêu thụ thức ăn nước uống tương đương với nhóm chứng.

1.4.2. Quan sát dấu hiệu ngộ độc

- Không nhận thấy có biểu hiện ngộ độc ở các nhóm thử trong thời gian theo dõi. Chuột khỏe mạnh, nhanh nhẹn, lông mượt, ăn uống, vận động bình thường.

- Không có chuột chết trong quá trình thử nghiệm.

1.4.3. Khối lượng cơ thể chuột

- Kết quả theo dõi khối lượng của chuột ở nhóm chứng và các nhóm thử được thể hiện trong các Bảng 2.

Bảng 2. Kết quả theo dõi khối lượng chuột

**Nhóm (n = 10)**	**Khối lượng chuột** *(g)*			
	**Trước thử nghiệm**	**Sau 1 ngày**	**Sau 4 ngày**	**Sau 7 ngày**
**Nhóm chứng**	19,32 ± 0,66	20,82 ± 0,75	25,64 ± 0,68	30,17 ± 1,16
**Nhóm thử 1**	19,42 ± 0,93	20,93 ± 0,93	25,65 ± 1,14	30,36 ± 1,04
**Nhóm thử 2**	19,44 ± 0,97	21,03 ± 0,91	25,76 ± 0,86	30,65 ± 1,10
**Nhóm thử 3**	19,52 ± 0,84	21,06 ± 0,83	25,90 ± 0,81	30,57 ± 1,39

- Kết quả so sánh cân nặng của chuột thí nghiệm giữa các nhóm thử và nhóm chứng được thể hiện ở Bảng 3.

Bảng 3. Bảng so sánh cân nặng giữa nhóm chứng và các nhóm thử

**Nhóm**	**Trước thử nghiệm**		**Sau thử nghiệm**		**Tăng khối lượng** *(%)*	$P_{trước-sau}$
	**Khối lượng chuột** *(g)*	$P_{trước}$	**Khối lượng chuột** *(g)*	P_{sau}		
**Chứng** (C)	19,32 ± 0,66	$P_{ANOVA} > 0,05$	30,17 ± 1,16	$P_{ANOVA} > 0,05$	156,2	P < 0,001

**Thử 1** (T1)	19,42 ± 0,93	$P_{T1-C} > 0,05$	30,36 ± 1,04	$P_{T1-C} > 0,05$	156,5	$P < 0,001$
**Thử 2** (T2)	19,44 ± 0,97	$P_{T2-C} > 0,05$	30,65 ± 1,10	$P_{T2-C} > 0,05$	157,8	$P < 0,001$
**Thử 3** (T3)	19,52 ± 0,84	$P_{T3-C} > 0,05$	30,57 ± 1,39	$P_{T3-C} > 0,05$	156,7	$P < 0,001$

Nhận xét:

Kết quả theo dõi khối lượng trung bình của chuột trong quá trình thử nghiệm 7 ngày cho thấy:

- Trước khi uống mẫu thử: Khối lượng trung bình của chuột ở các nhóm thử trước khi đưa vào thử nghiệm không có sự khác biệt có ý nghĩa thống kê so với nhau và so với nhóm chứng ($P_{ANOVA\ trước} > 0,05$; $P_{(T-C)\ trước} > 0,05$; $P_{(T-T)\ trước} > 0,05$).

- Sau uống mẫu thử 7 ngày: Chuột thí nghiệm ở nhóm chứng và nhóm thử đều tăng cân. Có sự khác biệt đáng kể về khối lượng của chuột khi so sánh với trước thử nghiệm trong mỗi nhóm ($P_{trước-sau} < 0,001$). Không có sự khác biệt có ý nghĩa thống kê về cân nặng trung bình sau thử nghiệm giữa các nhóm thử với nhau và so với nhóm chứng ($P_{ANOVA\ sau} > 0,05$; $P_{(T-C)\ sau} > 0,05$; $P_{(T-T)\ sau} > 0,05$).

1.4.4. Kết quả quan sát đại thể

Sau khi kết thúc thử nghiệm, chuột được mổ để quan sát đại thể. Kết quả quan sát đại thể cho thấy không có sự khác biệt ở các cơ quan nội tạng (tim, gan, lách, thận, phổi,...) so với nhóm chứng (Bảng 4).

Bảng 4. Kết quả mổ quan sát đại thể

**STT**	**Nhóm**	**Các phát hiện đại thể**
1	Chứng	Không có biểu hiện bất thường quan sát được
2	Thử 1	Không có sự bất thường so với nhóm chứng
3	Thử 2	Không có sự bất thường so với nhóm chứng
4	Thử 3	Không có sự bất thường so với nhóm chứng

1.5. Kết luận

Mẫu thử men tiêu hóa **LiveSpo COLON/COLYTIS** gửi tới yêu cầu thử độc tính cấp trên chuột nhắt trắng có kết quả như sau:

Cho chuột uống mẫu thử với mức liều từ 20,0 ml mẫu thử (chứa 12 x 10^9 CFU bào tử *Bacillus subtilis và Bacillus clausii*)/kg chuột đến 60,0 ml mẫu thử (chứa 36 x 10^9 CFU bào tử *Bacillus subtilis và Bacillus clausii*)/kg chuột, không nhận thấy có biểu hiện bất thường so với nhóm chứng. Chuột ăn uống, hoạt động bình thường, không có chuột chết.

Xác định được liều gây chết 50 % động vật thí nghiệm (LD_{50}) lớn hơn 60,0 ml (tương đương với 59310 mg) mẫu thử/kg chuột. Xác định được liều không gây chết động vật thí nghiệm (LD_0) là 60,0 ml mẫu thử/kg chuột. Xác định được liều không gây biểu hiện bất thường trên động vật thí nghiệm là 60,0 ml mẫu thử/kg chuột. Quan sát đại thể các cơ quan nội tạng của chuột sau khi kết thúc thí nghiệm không nhận thấy bất thường so với nhóm chứng.

Theo phân loại độc tính của GHS (Globally Harmonized System of Classification and Labelling of Chemicals, 2021), những chất/hợp chất có giá trị độc tính cấp LD_{50} lớn hơn 5000 mg/kg chuột theo đường uống được coi là độc tính thấp và không phân loại (unclassified). Dựa trên kết quả thu được của thử nghiệm này có thể kết luận mẫu thử men tiêu hóa **LiveSpo COLON/COLYTIS** có độc tính thấp dưới ngưỡng phân loại của GHS.

2. Thử nghiệm độc tính bán trường diễn

Thời gian thực hiện: Từ 19/07/2022 đến 30/08/2022

2.1. Động vật thí nghiệm

- Loài, giống: Thỏ Newzealand trưởng thành cả hai giống đực và cái, khỏe mạnh, thỏ cái không mang thai hoặc cho con bú, chưa trải qua bất kỳ thử nghiệm nào trước đó, cân nặng khoảng 1,8 – 2,2 kg.

- Số lượng: 21 con được chia ngẫu nhiên thành 3 nhóm thử nghiệm (1 nhóm chứng và 2 nhóm uống mẫu thử), mỗi nhóm 07 con.

- Nguồn gốc: Bộ phận chăn nuôi - Khoa Dược lý - Viện Kiểm nghiệm thuốc Trung Ương.

- Điều kiện chăm sóc: Thỏ được nuôi mỗi con một lồng trong phòng nuôi có kiểm soát nhiệt độ và độ ẩm thích hợp với thức ăn và nước uống theo nhu cầu. Tất cả các thao

tác trên động vật thí nghiệm đều được tuân theo các quy trình về chăm sóc và sử dụng động vật thí nghiệm của Khoa Dược lý – Viện Kiểm nghiệm thuốc Trung Ương.

2.2. Tiến hành

2.2.1. Chuẩn bị mẫu thử

- Lựa chọn mức liều thử nghiệm: Dựa trên liều tối đa dự kiến dùng trên người là 3 ống (15 ml hỗn dịch mẫu thử)/người/ngày và sử dụng hệ số chuyển đổi liều giữa thỏ và người là 3,1 để lựa chọn 2 mức liều thử nghiệm là:

+ Liều tương ứng với mức liều dự kiến cho người: 0,93 ml hỗn dịch mẫu thử/kg thỏ/ngày.

+ Liều cao gấp 5 lần liều dự kiến cho người: 4,65 ml hỗn dịch mẫu thử/kg thỏ/ngày.

- Cách xử lý và chuẩn bị mẫu thử:

+ *Mẫu đối chứng*: Nước

+ *Hỗn dịch A (liều cao gấp 5 liều dự kiến cho người):* Dùng nguyên mẫu

+ *Hỗn dịch B (liều tương ứng với liều dự kiến cho người):* Pha loãng 20 ml hỗn dịch A với nước vừa đủ 100 ml.

2.2.2. Bố trí thử nghiệm

Thử nghiệm được tiến hành trên 21 thỏ, chia thành 3 nhóm: mỗi nhóm 07 con. Bố trí thử nghiệm và thử với các mức liều theo Bảng 5.

Bảng 5. Các mức liều thử nghiệm bán trường diễn trên thỏ

Nhóm	Số thỏ thí nghiệm	Thể tích cho uống *(ml/kg thỏ)*	Liều dùng *(ml mẫu thử/kg thỏ)*
Chứng	07	4,65 ml nước/kg thỏ	---
Thử 1	07	4,65 ml hỗn dịch B/kg thỏ	0,93 ml/kg thỏ/ngày
Thử 2	07	4,65 ml hỗn dịch A/kg thỏ	4,65 ml/kg thỏ/ngày

2.2.3. Theo dõi và đánh giá

- Theo dõi thỏ hàng ngày về mức độ tiêu thụ thức ăn, nước uống, thể trạng và vận động, tình trạng phân, nước tiểu, các biểu hiện bất thường (nếu có) của thỏ.

- Xác định cân nặng của thỏ tại các thời điểm 0, 7, 14, 21, 28 ngày uống mẫu thử và 14 ngày sau khi ngừng uống mẫu thử.

- Xét nghiệm các chỉ số huyết học liên quan tới chức năng tạo máu (số lượng hồng cầu, bạch cầu, tiểu cầu, hemoglobin, hematocrit), các chỉ số liên quan tới chức năng gan (AST, ALT, protein toàn phần, bilirubin toàn phần, cholesterol, albumin), các chỉ số liên quan tới chức năng thận (creatinin, urea), chỉ số glucose tại các thời điểm 0, 14, 28 ngày uống mẫu thử và 14 ngày sau khi ngừng uống mẫu thử. So sánh kết quả của nhóm thử và nhóm chứng theo phương pháp thống kê.

- Sau thử nghiệm động vật được mổ để quan sát đại thể các tổ chức tim, gan, thận, phổi, dạ dày, ruột của tất cả các thỏ.

- Lấy ngẫu nhiên 03 thỏ/nhóm, tiến hành làm tiêu bản giải phẫu mô bệnh học gan, thận, ruột non, ruột già để đánh giá vi thể các tổ chức trên ngay sau khi ngừng uống mẫu thử.

2.2.4. Trình bày và xử lý số liệu

Số liệu thực nghiệm được trình bày dưới dạng giá trị trung bình cộng trừ độ lệch chuẩn (mean ± SD) và được xử lý thống kê bằng trắc nghiệm Student để so sánh sự khác nhau của cùng một chỉ số giữa nhóm chứng và nhóm thử.

2.3. Kết quả

2.3.1. Tình trạng thỏ

Trong thời gian thử nghiệm, tất cả các thỏ đều hoạt động bình thường, ăn uống tốt, mắt sáng, lông mượt, phân khô. Không có biểu hiện bất thường về thể trạng, ăn uống cũng như vận động.

Theo dõi cân nặng thỏ trong quá trình thử nghiệm cho thấy:

- Trước thử nghiệm (trước khi uống mẫu thử): Cân nặng trung bình của thỏ ở các nhóm thử trước khi đưa vào thử nghiệm không có sự khác biệt so với nhóm chứng ($P_{trước(T1-C)} > 0,05$; $P_{trước(T2-C)} > 0,05$).

- Sau 28 ngày uống mẫu thử: Thỏ ở nhóm chứng và hai nhóm thử đều tăng cân ở mỗi thời điểm đánh giá. Có sự khác biệt có ý nghĩa về cân nặng của thỏ khi so sánh sau 28 ngày thử nghiệm với trước thử nghiệm trong mỗi nhóm ($P_{trước-sau} < 0,01$). Không có sự khác biệt có ý nghĩa về cân nặng trung bình giữa nhóm thử so với nhóm chứng ($P_{sau(T1-C)} > 0,05$; $P_{sau(T2-C)} > 0,05$).

- Sau 14 ngày ngừng uống mẫu thử: Thỏ khỏe mạnh tăng cân tốt, không có sự khác biệt có ý nghĩa về cân nặng trung bình giữa hai nhóm thử so với nhóm chứng ($P_{sau(T1-C)} > 0,05$; $P_{sau(T2-C)} > 0,05$).

Bảng 6. Kết quả theo dõi cân nặng của thỏ trong thời gian uống mẫu thử

Nhóm (n = 7)	Khối lượng cơ thể (kg)					P
	TrướcTN (m_0)	*Sau 7 ngày (m_1)*	*Sau 14 ngày (m_2)*	*Sau 21 ngày(m_3)*	*Sau 28 ngày (m_4)*	
**Chứng (C)**	1,99 ± 0,13	2,13 ± 0,17	2,22 ± 0,05	2,31 ± 0,09	2,38 ± 0,10	$P_{trước-sau} < 0,01$
% so với trước thử nghiệm		106,8 %	111,5 %	116,5 %	119,9 %	
**Thử 1 (T1)**	1,96 ± 0,13	2,10 ± 0,15	2,20 ± 0,15	2,28 ± 0,18	2,37 ± 0,21	$P_{trước-sau} < 0,001$<br>$P_{trước(T1-C)} > 0,05$
% so với trước thử nghiệm		107,2 %	112,4 %	116,6 %	121,0 %	$P_{sau(T1-C)} > 0,05$
**Thử 2 (T2)**	1,99 ± 0,17	2,11 ± 0,17	2,21 ± 0,14	2,30 ± 0,15	2,39 ± 0,17	$P_{trước-sau} < 0,001$<br>$P_{trước(T2-C)} > 0,05$
% so với trước thử nghiệm		105,9 %	111,2 %	116,1 %	120,2 %	$P_{sau(T2-C)} > 0,05$

Bảng 7. Theo dõi cân nặng thỏ sau 14 ngày ngừng uống mẫu thử

**Nhóm (n = 4)**	**Khối lượng cơ thể (kg)**	$P_{sau\ 14\ ngày\ (T-C)}$
Chứng (C)	2,46 ± 0,09	
Thử 1 (T1)	2,35 ± 0,06	> 0,05
Thử 2 (T2)	2,41 ± 0,13	> 0,05

2.3.2. Kết quả theo dõi các chỉ số huyết học liên quan tới chức năng tạo máu

a. Trước thử nghiệm (trước khi uống mẫu thử)

Bảng 8. Các chỉ số huyết học trước khi dùng mẫu thử

**Chỉ tiêu**	**Nhóm chứng (n = 7)**	**Nhóm T1 (n = 7)**	***P** (T1-C)*	**Nhóm T2 (n = 7)**	***P** (T2-C)*
**Hồng cầu** *(x 10^{12}/l)*	5,4 ± 0,5	5,2 ± 0,5	> 0,05	5,2 ± 0,4	> 0,05
**Bạch cầu** *(x 10^9/l)*	6,9 ± 2,1	7,1 ± 1,4	> 0,05	6,7 ± 2,0	> 0,05

**Tiểu cầu** *(x 10^9/l)*	361,1 ± 130,6	332,6 ± 65,6	> 0,05	365,4 ± 116,5	> 0,05
**Hematocrit** *(%)*	36,7 ± 2,9	34,8 ± 2,9	> 0,05	35,6 ± 1,6	> 0,05
**Hemoglobin** *(g/dl)*	11,4 ± 1,0	10,9 ± 1,0	> 0,05	11,1 ± 0,6	> 0,05

b. Thời điểm sau 14 ngày uống mẫu thử

Bảng 9. Các chỉ số huyết học sau 14 ngày dùng mẫu thử

Chỉ tiêu	Nhóm chứng (n = 7)	Nhóm T1 (n = 7)	$P_{(T1-C)}$	Nhóm T2 (n = 7)	$P_{(T2-C)}$
**Hồng cầu** *(x 10^{12}/l)*	5,7 ± 0,5	5,3 ± 0,5	> 0,05	5,2 ± 0,4	> 0,05
**Bạch cầu** *(x 10^9/l)*	6,3 ± 2,0	7,6 ± 1,8	> 0,05	7,1 ± 2,1	> 0,05
**Tiểu cầu** *(x 10^9/l)*	325,7 ± 47,5	369,7 ± 83,0	> 0,05	369,9 ± 51,4	> 0,05
**Hematocrit** *(%)*	38,2 ± 2,6	36,7 ± 2,9	> 0,05	36,2 ± 2,2	> 0,05
**Hemoglobin** *(g/dl)*	12,0 ± 0,8	11,3 ± 0,9	> 0,05	11,2 ± 0,6	> 0,05

c. Thời điểm sau 28 ngày uống mẫu thử

Bảng 10. Các chỉ số huyết học sau 28 ngày dùng mẫu thử

Chỉ tiêu	Nhóm chứng (n = 7)	Nhóm T1 (n = 7)	$P_{(T1-C)}$	Nhóm T2 (n = 7)	$P_{(T2-C)}$
**Hồng cầu** *(x 10^{12}/l)*	5,6 ± 0,5	5,4 ± 0,4	> 0,05	5,4 ± 0,2	> 0,05
**Bạch cầu** *(x 10^9/l)*	9,0 ± 1,9	10,1 ± 1,6	> 0,05	8,6 ± 1,5	> 0,05
**Tiểu cầu** *(x 10^9/l)*	348,0 ± 123,2	364,7 ± 104,4	> 0,05	408,6 ± 138,6	> 0,05
**Hematocrit** *(%)*	37,8 ± 3,2	36,6 ± 2,7	> 0,05	37,1 ± 1,9	> 0,05

**Hemoglobin** *(g/dl)*	12,2 ± 0,8	11,6 ± 0,8	> 0,05	11,8 ± 0,6	> 0,05

d. Thời điểm sau 14 ngày ngừng uống mẫu thử

Bảng 11. Các chỉ số huyết học sau 14 ngày ngừng uống mẫu thử

Chỉ tiêu	Nhóm chứng (n = 4)	Nhóm T1 (n = 4)	$P_{(T1-C)}$	Nhóm T2 (n = 4)	$P_{(T2-C)}$
**Hồng cầu** *($x\ 10^{12}/l$)*	5,7 ± 0,6	5,7 ± 0,3	> 0,05	5,7 ± 0,5	> 0,05
**Bạch cầu** *($x\ 10^{9}/l$)*	9,3 ± 1,0	11,0 ± 2,2	> 0,05	11,5 ± 2,7	> 0,05
**Tiểu cầu** *($x\ 10^{9}/l$)*	293,0 ± 65,5	370,8 ± 42,8	> 0,05	449,3 ± 157,8	> 0,05
**Hematocrit** *(%)*	38,1 ± 3,1	37,9 ± 1,7	> 0,05	38,0 ± 1,4	> 0,05
**Hemoglobin** *(g/dl)*	12,4 ± 0,8	12,3 ± 0,7	> 0,05	12,4 ± 0,6	> 0,05

Nhận xét

Kết quả xét nghiệm một số chỉ số huyết học cho thấy:

- Trước uống mẫu thử: Không có sự khác biệt có ý nghĩa về chỉ số huyết học giữa nhóm chứng và 2 nhóm thử ($P_{trước\ TN\ (T-C)} > 0,05$).

- Sau 14 ngày, 28 ngày uống mẫu thử và sau 14 ngày ngừng uống mẫu thử: Không có sự khác biệt có ý nghĩa về chỉ số huyết học giữa nhóm chứng và 2 nhóm thử ($P_{sau\ 14ngày\ (T-C)} > 0,05$; $P_{sau\ 28\ ngày\ (T-C)} > 0,05$), $P_{sau\ 14\ ngày\ ngừng\ uống\ mẫu\ thử (T-C)} > 0,05$).

2.3.3. Kết quả theo dõi các chỉ số liên quan tới chức năng gan

a. Trước thử nghiệm (trước khi uống mẫu thử)

Bảng 12. Các chỉ số liên quan chức năng gan trước khi dùng mẫu thử

Chỉ tiêu	Nhóm chứng (n = 7)	Nhóm T1 (n = 7)	$P_{(T1-C)}$	Nhóm T2 (n = 7)	$P_{(T2-C)}$
**AST** *(U/l)*	40,2 ± 10,6	54,3 ± 22,9	> 0,05	50,8 ± 18,9	> 0,05
**ALT** *(U/l)*	66,8 ± 15,3	60,7 ± 20,0	> 0,05	66,6 ± 14,6	> 0,05
**Bilirubin toàn phần** *(µmol/l)*	2,0 ± 0,9	2,0 ± 1,0	> 0,05	3,0 ± 1,5	> 0,05

**Protein toàn phần** *(g/l)*	51,5 ± 4,6	49,3 ± 2,6	> 0,05	51,1 ± 4,3	> 0,05
**Albumin** *(g/dl)*	34,7 ± 2,1	34,2 ± 1,1	> 0,05	33,8 ± 1,7	> 0,05
**Cholesterol** *(mmol/l)*	2,9 ± 1,0	3,0 ± 0,9	> 0,05	2,4 ± 0,7	> 0,05

b. Thời điểm sau 14 ngày uống mẫu thử

Bảng 13. Các chỉ số liên quan chức năng gan sau 14 ngày dùng mẫu thử

Chỉ tiêu	Nhóm chứng (n = 7)	Nhóm T1 (n = 7)	$P_{(T1-C)}$	Nhóm T2 (n = 7)	$P_{(T2-C)}$
**AST** *(U/l)*	46,9 ± 16,3	60,4 ± 21,9	> 0,05	46,3 ± 15,1	> 0,05
**ALT** *(U/l)*	73,0± 24,4	68,4 ± 26,3	> 0,05	68,0 ± 20,8	> 0,05
**Bilirubin toàn phần** *(μmol/l)*	1,9 ± 0,5	2,5 ± 0,8	> 0,05	1,6 ± 0,6	> 0,05
**Protein toàn phần** *(g/l)*	53,5 ± 4,0	52,9 ± 3,9	> 0,05	52,8 ± 3,6	> 0,05
**Albumin** *(g/dl)*	35,1 ± 1,8	35,5 ± 2,1	> 0,05	33,8 ± 1,2	> 0,05
**Cholesterol** *(mmol/l)*	3,7 ± 1,1	3,5 ± 1,2	> 0,05	3,4 ± 0,8	> 0,05

c. Thời điểm sau 28 ngày uống mẫu thử

Bảng 14. Các chỉ số liên quan chức năng gan sau 28 ngày dùng mẫu thử

Chỉ tiêu	Nhóm chứng (n = 7)	Nhóm T1 (n = 7)	$P_{(T1-C)}$	Nhóm T2 (n = 7)	$P_{(T2-C)}$
**AST** *(U/l)*	50,2 ± 20,8	41,7 ± 20,1	> 0,05	40,1 ± 8,1	> 0,05
**ALT** *(U/l)*	65,6 ± 14,4	59,4 ± 20,9	> 0,05	55,7 ± 23,2	> 0,05

**Bilirubin toàn phần** *(µmol/l)*	2,0 ± 1,1	1,2 ± 0,6	> 0,05	1,5 ± 0,7	> 0,05
**Protein toàn phần** *(g/l)*	55,2 ± 3,0	54,2 ± 2,9	> 0,05	56,2 ± 4,2	> 0,05
**Albumin** *(g/dl)*	37,7 ± 2,3	37,1 ± 1,5	> 0,05	36,0 ± 1,3	> 0,05
**Cholesterol** *(mmol/l)*	2,9 ± 1,0	2,7 ± 1,1	> 0,05	2,4 ± 1,0	> 0,05

d. Thời điểm sau 14 ngày ngừng uống mẫu thử

Bảng 15. Các chỉ số liên quan chức năng gan sau 14 ngày ngừng uống mẫu thử

Chỉ tiêu	Nhóm chứng (n = 4)	Nhóm T1 (n = 4)	$P_{(T1-C)}$	Nhóm T2 (n = 4)	$P_{(T2-C)}$
**AST** *(U/l)*	31,3 ± 3,3	55,9 ± 22,3	> 0,05	42,0 ± 17,0	> 0,05
**ALT** *(U/l)*	76,7 ± 9,7	96,2 ± 20,7	> 0,05	59,3 ± 19,2	> 0,05
**Bilirubin toàn phần** *(µmol/l)*	1,8 ± 0,4	2,7 ± 1,0	> 0,05	1,3 ± 0,4	> 0,05
**Protein toàn phần** *(g/l)*	57,4 ± 2,8	58,2 ± 1,2	> 0,05	60,3 ± 4,6	> 0,05
**Albumin** *(g/dl)*	35,7 ± 1,2	37,2 ± 1,1	> 0,05	35,7 ± 0,6	> 0,05
**Cholesterol** *(mmol/l)*	3,2 ± 1,7	1,9 ± 0,4	> 0,05	1,9 ± 0,4	> 0,05

Nhận xét

Kết quả xét nghiệm một số chỉ số chức năng gan cho thấy:

- Trước uống mẫu thử: Không có sự khác biệt có ý nghĩa về các chỉ số liên quan đến chức năng gan giữa nhóm chứng và 2 nhóm thử ($P_{trướcTN\ (T-C)} > 0,05$).

- Sau 14 ngày uống mẫu thử, sau 28 ngày uống mẫu thử và sau 14 ngày ngừng uống mẫu thử: Không có sự khác biệt có ý nghĩa về các chỉ số liên quan đến chức năng gan giữa nhóm chứng và 2 nhóm thử ($P_{sau\ 14\ ngày(T-C)} > 0,05$; $P_{sau\ 28\ ngày(T-C)} > 0,05$), $P_{sau\ 14\ ngày\ ngừng\ uống\ mẫu\ thử(T-C)} > 0,05$).

2.3.4. Kết quả theo dõi các chỉ số liên quan tới chức năng thận

a. Trước thử nghiệm (trước khi uống mẫu thử)

Bảng 16. Các chỉ số liên quan chức năng thận trước khi dùng mẫu thử

Chỉ tiêu	Nhóm chứng (n = 7)	Nhóm T1 (n = 7)	$P_{(T1-C)}$	Nhóm T2 (n = 7)	$P_{(T2-C)}$
**Urea** *(mmol/l)*	4,9 ± 1,4	4,3 ± 0,8	> 0,05	4,0 ± 1,4	> 0,05
**Creatinin** *(µmol/l)*	105,3 ± 23,0	96,0 ± 14,5	> 0,05	85,2 ± 12,0	> 0,05

b. Thời điểm sau 14 ngày uống mẫu thử

Bảng 17. Các chỉ số liên quan chức năng thận sau 14 ngày dùng mẫu thử

Chỉ tiêu	Nhóm chứng (n = 7)	Nhóm T1 (n = 7)	$P_{(T1-C)}$	Nhóm T2 (n = 7)	$P_{(T2-C)}$
**Urea** *(mmol/l)*	5,2 ± 0,9	4,6 ± 1,2	> 0,05	4,5 ± 0,5	> 0,05
**Creatinin** *(µmol/l)*	111,9 ± 18,4	114,4 ± 19,8	> 0,05	96,9 ± 8,7	> 0,05

c. Thời điểm sau 28 ngày uống mẫu thử

Bảng 18. Các chỉ số liên quan chức năng thận sau 28 ngày dùng mẫu thử

Chỉ tiêu	Nhóm chứng (n = 7)	Nhóm T1 (n = 7)	$P_{(T1-C)}$	Nhóm T2 (n = 7)	$P_{(T2-C)}$
**Urea** *(mmol/l)*	5,3 ± 0,9	5,2 ± 1,2	> 0,05	4,6 ± 0,8	> 0,05
**Creatinin** *(µmol/l)*	110,9 ± 8,5	105,6 ± 11,7	> 0,05	104,6 ± 4,3	> 0,05

d. Thời điểm sau 14 ngày ngừng uống mẫu thử

Bảng 19. Các chỉ số liên quan chức năng thận sau 14 ngày ngừng uống mẫu thử

Chỉ tiêu	Nhóm chứng (n = 4)	Nhóm T1 (n = 4)	$P_{(T1-C)}$	Nhóm T2 (n = 4)	$P_{(T2-C)}$
**Urea** *(mmol/l)*	5,8 ± 1,0	6,0 ± 0,8	> 0,05	5,1 ± 0,7	> 0,05
**Creatinin** *(µmol/l)*	129,2 ± 13,8	131,4 ± 15,2	> 0,05	112,7 ± 14,8	> 0,05

Nhận xét

Kết quả xét nghiệm urea và creatinin cho thấy:

- Trước uống mẫu thử: Không có sự khác biệt có ý nghĩa về các chỉ số liên quan đến chức năng thận giữa nhóm chứng và 2 nhóm thử ($P_{trướcTN\ (T-C)}$ > **0,05**).

- Sau 14 ngày uống mẫu thử, sau 28 ngày uống mẫu thử và sau 14 ngày ngừng uống mẫu thử: Không có sự khác biệt có ý nghĩa về các chỉ số liên quan đến chức năng thận giữa nhóm chứng và 2 nhóm thử ($P_{sau\ 14\ ngày(T-C)}$ > 0,05; $P_{sau\ 28\ ngày(T-C)}$ > 0,05), $P_{sau\ 14\ ngày\ ngừng\ uống\ mẫu\ thử(T-C)}$ > 0,05).

2.3.5. Kết quả theo dõi chỉ số glucose trong huyết tương

a. Trước thử nghiệm (trước khi uống mẫu thử)

Bảng 20. Chỉ số glucose trước khi dùng mẫu thử

Chỉ tiêu	Nhóm chứng (n = 7)	Nhóm T1 (n = 7)	$P_{(T1-C)}$	Nhóm T2 (n = 7)	$P_{(T2-C)}$
**Glucose** *(mmol/l)*	6,4 ± 1,6	7,1 ± 1,0	> 0,05	7,2 ± 0,8	> 0,05

b. Thời điểm sau 14 ngày uống mẫu thử

Bảng 21. Chỉ số glucose sau 14 ngày dùng mẫu thử

Chỉ tiêu	Nhóm chứng (n = 7)	Nhóm T1 (n = 7)	$P_{(T1-C)}$	Nhóm T2 (n = 7)	$P_{(T2-C)}$
**Glucose** *(mmol/l)*	6,7 ± 0,9	6,3 ± 0,4	> 0,05	6,4 ± 0,8	> 0,05

c. Thời điểm sau 28 ngày uống mẫu thử

Bảng 22. Chỉ số glucose sau 28 ngày dùng mẫu thử

Chỉ tiêu	Nhóm chứng (n = 7)	Nhóm T1 (n = 7)	$P_{(T1-C)}$	Nhóm T2 (n = 7)	$P_{(T2-C)}$
Glucose *(mmol/l)*	6,6 ± 0,8	6,8 ± 0,7	> 0,05	6,6 ± 0,5	> 0,05

d. Thời điểm sau 14 ngày ngừng uống mẫu thử

Bảng 23. Chỉ số glucose sau 14 ngày ngừng uống mẫu thử

Chỉ tiêu	Nhóm chứng (n = 4)	Nhóm T1 (n = 4)	$P_{(T1-C)}$	Nhóm T2 (n = 4)	$P_{(T2-C)}$
Glucose *(mmol/l)*	6,3 ± 1,0	5,8 ± 0,6	> 0,05	5,4 ± 0,3	> 0,05

Nhận xét

Kết quả xét nghiệm glucose cho thấy:

- Trước uống mẫu thử: Không có sự khác biệt có ý nghĩa về chỉ số glucose giữa nhóm chứng và 2 nhóm thử ($P_{trướcTN\ (T-C)} > 0,05$).

- Sau 14 ngày uống mẫu thử, sau 28 ngày uống mẫu thử và sau 14 ngày ngừng uống mẫu thử: Không có sự khác biệt có ý nghĩa về các chỉ số glucose giữa nhóm chứng và 2 nhóm thử ($P_{sau\ 14\ ngày(T-C)} > 0,05$; $P_{sau\ 28\ ngày(T-C)} > 0,05$), $P_{sau\ 14\ ngày\ ngừng\ uống\ mẫu\ thử(T-C)} > 0,05$).

2.3.6. Quan sát đại thể

Kết quả quan sát đại thể các cơ quan nội tạng của tất cả các thỏ thử nghiệm cho thấy: Không có biểu hiện khác thường về hình dạng bên ngoài, màu sắc của các tổ chức tim, phổi, gan, lách, thận, dạ dày, ruột của các thỏ nhóm thử so với nhóm chứng sau thử nghiệm.

Bảng 24. Hình ảnh đại thể các cơ quan nội tạng

Hình ảnh đại thể		
Nhóm Chứng	*Nhóm Thử 1 (liều thấp)*	*Nhóm Thử 2 (liều cao)*
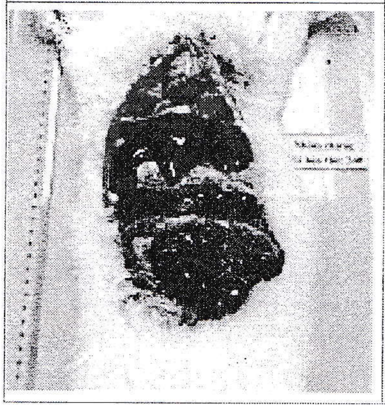	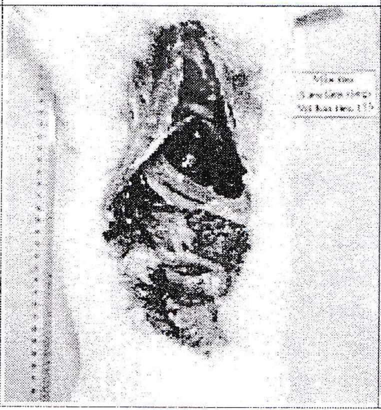	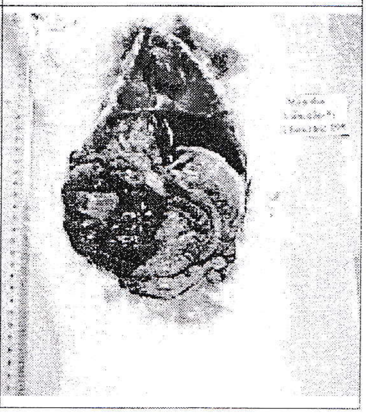

2.3.7. Quan sát vi thể

Tiêu bản gan, thận, ruột non, đại tràng được cố định bằng Formalin 10 %, nhuộm bằng dung dịch nhuộm Hematoxylin Eosin (HE) và Perioric Acid Shiff (PAS) và quan sát dưới kính hiển vi quang học.

Kết quả quan sát vi thể do Bộ môn Giải phẫu sinh lý bệnh - Trường Đại học Y Hà Nội thực hiện cho thấy: Các thỏ thử nghiệm đều có gan, thận, ruột non, đại tràng không bị tổn thương, hình ảnh cấu trúc trong giới hạn bình thường. Không có các triệu chứng bất thường liên quan đến mẫu thử với 2 mức liều khác nhau so với nhóm chứng.

Cụ thể như sau:

Gan:

- Tế bào gan: Không thấy tổn thương.
- Mao mạch nan hoa: Bình thường.
- Tĩnh mạch trung tâm: Bình thường.
- Khoảng cửa: Bình thường.
- Mô kẽ: Bình thường.

Kết luận: Mô gan bình thường, không thấy tổn thương.

Thận:

- Tiểu cầu thận (mao mạch, gian mạch, bao Baomann): Bình thường.
- Ống thận: Bình thường.
- Đài bể thận: Bình thường.

- Mô kẽ: Bình thường.

Kết luận: Mô thận bình thường, không thấy tổn thương.

Ruột non:

- Niêm mạc (nhung mao, tế bào hấp thu, tế bào hình đài, mô đệm, cơ niêm): Bình thường.
- Dưới niêm mạc: Bình thường.
- Lớp cơ: Bình thường.
- Thanh mạc: Bình thường.

Kết luận: Mô ruột non bình thường, không thấy tổn thương.

Đại tràng:

- Niêm mạc (biểu mô trụ đơn, tế bào hình đài tiết nhầy, tuyến Lieberkuhn, mô đệm, cơ niêm): Bình thường.
- Dưới niêm mạc: Bình thường.
- Lớp cơ: Bình thường.
- Thanh mạc: Bình thường.

Kết luận: Mô đại tràng bình thường, không thấy tổn thương.

Bảng 25. Hình ảnh giải phẫu mô bệnh học gan, thận, ruột non, đại tràng (Hình ảnh nhuộm HE, Độ phóng đại 400 lần)

STT	*Nhóm*	*Hình ảnh mô gan*	*Hình ảnh mô thận*	*Hình ảnh mô ruột non*	*Hình ảnh mô đại tràng*
1	***Chứng***	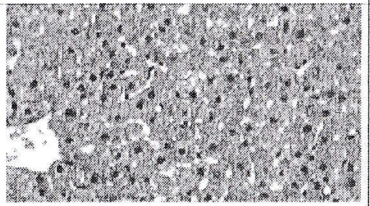<br>Mô gan bình thường, không thấy tổn thương.	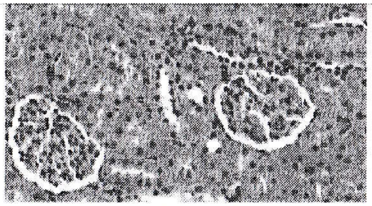<br>Mô thận bình thường, không thấy tổn thương.	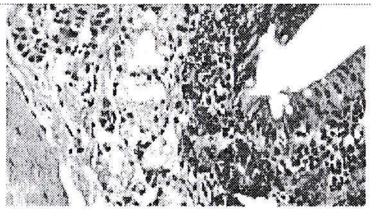<br>Mô ruột non bình thường, không thấy tổn thương.	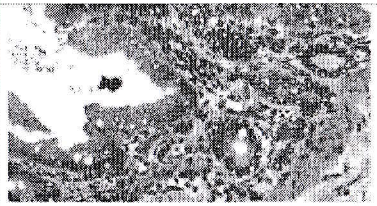<br>Mô đại tràng bình thường, không thấy tổn thương.
2	***Thử 1 (liều thấp)***	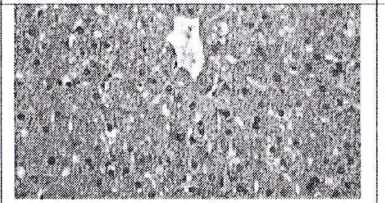<br>Mô gan bình thường, không thấy tổn thương.	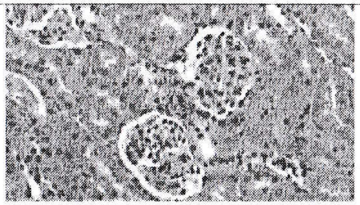<br>Mô thận bình thường, không thấy tổn thương.	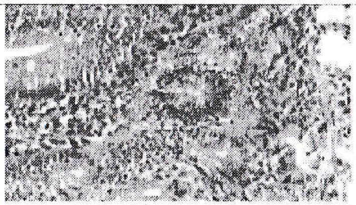<br>Mô ruột non bình thường, không thấy tổn thương.	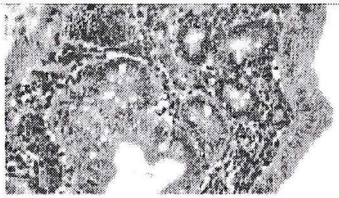<br>Mô đại tràng bình thường, không thấy tổn thương.
3	***Thử 2 (liều cao)***	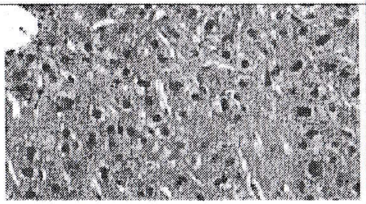<br>Mô gan bình thường, không thấy tổn thương.	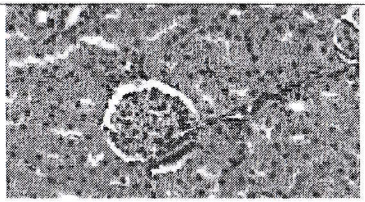<br>Mô thận bình thường, không thấy tổn thương.	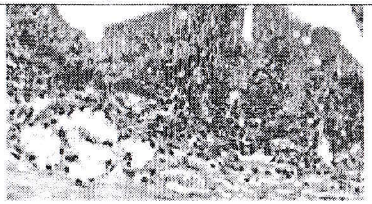<br>Mô ruột non bình thường, không thấy tổn thương.	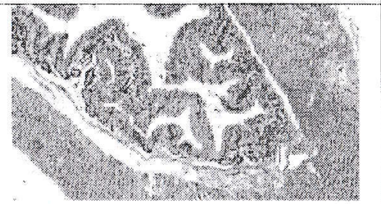<br>Mô đại tràng bình thường, không thấy tổn thương.

Bảng 26. Hình ảnh giải phẫu mô bệnh học gan, thận, ruột non, đại tràng (Hình ảnh nhuộm PAS, Độ phóng đại 400 lần)

STT	Nhóm	Hình ảnh mô gan	Hình ảnh mô thận	Hình ảnh mô ruột non	Hình ảnh mô đại tràng
1	Chứng	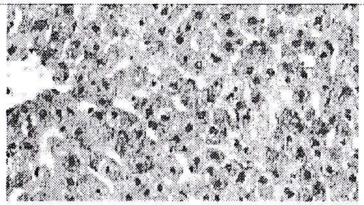 Mô gan bình thường, không thấy tổn thương.	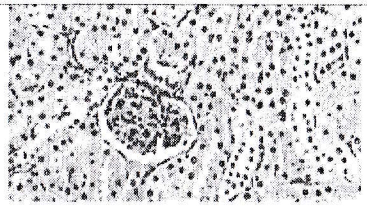 Mô thận bình thường, không thấy tổn thương.	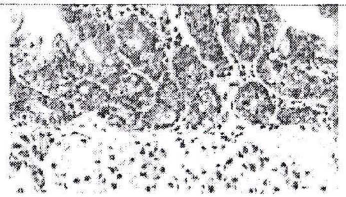 Mô ruột non bình thường, không thấy tổn thương.	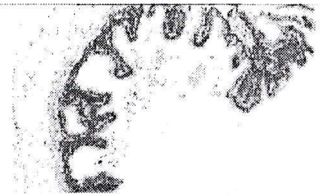 Mô đại tràng bình thường, không thấy tổn thương.
2	Thử 1 (liều thấp)	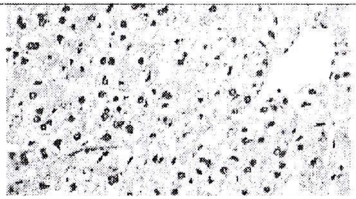 Mô gan bình thường, không thấy tổn thương.	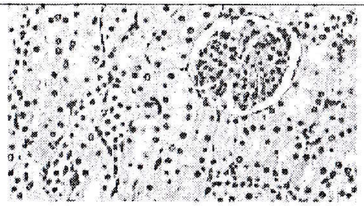 Mô thận bình thường, không thấy tổn thương.	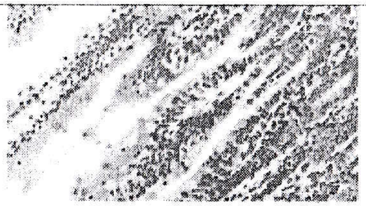 Mô ruột non bình thường, không thấy tổn thương.	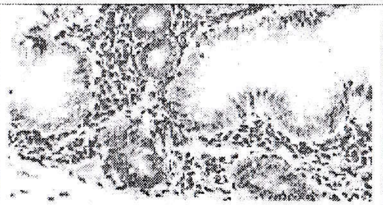 Mô đại tràng bình thường, không thấy tổn thương.
3	Thử 2 (liều cao)	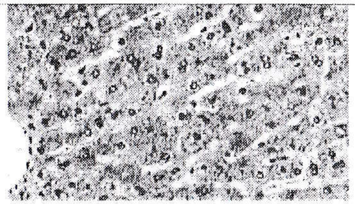 Mô gan bình thường, không thấy tổn thương.	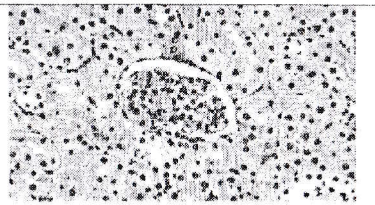 Mô thận bình thường, không thấy tổn thương.	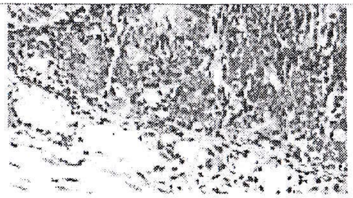 Mô ruột non bình thường, không thấy tổn thương.	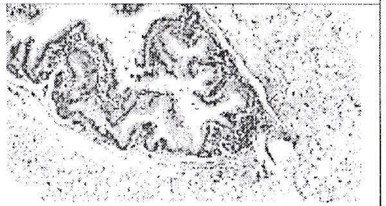 Mô đại tràng bình thường, không thấy tổn thương.

2.4. Kết luận

Mẫu thử men tiêu hóa **LiveSpo COLON/COLYTIS** gửi tới yêu cầu thử độc tính bán trường diễn trên thỏ có kết quả như sau:

Sau khi cho thỏ uống hỗn dịch mẫu thử liên tục trong 28 ngày với 2 mức liều khác nhau là 0,93 ml/kg thỏ/ngày (tương ứng với mức liều tối đa dùng cho người là 3 ống/người/ngày hay tương đương với 9 tỷ CFU *Bacillus subtilis* và *Bacillus clausii* /người/ngày) và 4,65 ml/kg thỏ/ngày (cao gấp 5 lần so với liều dùng tối đa ngoại suy từ liều dùng của người, tương đương với tương đương với 45 tỷ CFU *Bacillus subtilis* và *Bacillus clausii* /người/ngày), mẫu thử không gây ảnh hưởng đến cân nặng, thể trạng, vận động của thỏ thí nghiệm. Thỏ khỏe mạnh, tăng cân.

Về các chỉ số sinh hóa đánh giá chức năng gan, thận (hoạt độ các enzyme AST, ALT, protein toàn phần, bilirubin toàn phần, cholesterol, albumin, glucose, urea, creatinin) và các chỉ số huyết học (hồng cầu, hemoglobin, hematocrit, bạch cầu, tiểu cầu) không có sự khác biệt có ý nghĩa ở trước thử nghiệm, sau 14 ngày uống mẫu thử, sau 28 ngày uống mẫu thử, và sau 14 ngày ngừng uống mẫu thử giữa hai nhóm thử nghiệm so với nhóm chứng.

Không nhận thấy bất thường ở các tổ chức tim, phổi, gan, lách, thận, dạ dày, ruột của thỏ thí nghiệm khi quan sát đại thể cũng như không nhận thấy tổn thương mô bệnh học của gan, thận, ruột non, đại tràng khi quan sát vi thể giữa hai nhóm thử và nhóm chứng.

Hà Nội, ngày 26 tháng 9 năm 2022

Xác nhận của cơ quan	KHOA DƯỢC LÝ
VIỆN TRƯỞNG	
Đoàn Cao Sơn	TS. Nguyễn Thị Liên
