# Supplementary material for: Bacillus spore probiotics for alleviating functional constipation in children: a randomized, double-blind, placebo-controlled trial
Source: Commun Med (Lond). 2026 Mar 18;6:148. doi: 10.1038/s43856-026-01517-6 (PMC13000159; doi:10.1038/s43856-026-01517-6)
Supplement: Supplementary file 4 — Supplementary Data 2 [file 43856_2026_1517_MOESM4_ESM.pdf]

NATIONAL INSTITUTE OF DRUG QUALITY CONTROL
LABORATORY OF PHARMACOLOGY

TEST RESULTS

ACUTE TOXICITY AND SUB-ACUTE TOXICITY

(Analytical results are valid for the test samples only)

GENERAL INFORMATION

Sample:	**3-*Bacillus* mixture ingredient for the probiotic product LiveSpo® Preg-mom and LiveSpo® Dia30**
Producer:	LiveSpo Pharma Company Limited
LOT:	1223A
Date of manufacture:	01/02/2023
Expiration Date:	31/01/2025
Sample sender:	LiveSpo Pharma Company Limited
Test criteria:	Acute toxicity and sub-acute toxicity test
Test documents:	1. Methods for determining the toxicity of the drug - Medical Publisher, 2014<br>2. OECD guidelines for testing of chemicals. Repeated dose 28 - days Oral Toxicity study in Rodents OECD 407. 2008.
Formulations on the label:	*Bacillus subtilis, Bacillus clausii* and *Bacillus coagulans* spores at the concentration of 5 x 10^9 CFU, in the formed of ingredients for the probiotic products LiveSpo® Preg-mom and LiveSpo® Dia30
Sample status when remove the seals for experiment:	The sample is packed in white plastic ampoules, each ampoule contains 5 mL of spores.
Place of testing:	Laboratory of Pharmacology – National Institute of Drug Quality Control
Address:	Tam Hiep, Thanh Tri, Ha Noi
Headquarter:	48 Hai BaTrung, Ha Noi

TEST RESULTS

1. Acute toxicity test

Implementation period: From 24/5/2023 to 31/5/2023

1.1. Experimental animals

- Species: Swiss white mice.
- Weight: 18 - 22 g.
- Quantity: 30 mice.
- Source of supply: National Institute of Hygiene and Epidemiology.
- Care conditions: Mice are caged in small groups of 6-8 individuals, placed in a temperature and humidity-controlled room, food and drinking water was supplied on demand. All manipulations on experimental animals were followed the procedures for care and use of experimental animals by the Laboratory of Pharmacology – National Institute of Drug Quality Control.

1.2. Experiment

Mice were fasted for 3-4 hours before entering the experiment; drinking water was supplied on demand. Mice's weight was checked before the test. Mice that met the weight requirements were included in the experiment.

- Preparation of the test samples: Use the samples as provided
- Control: Cooled boiling water.
- How to deliver the sample into the mice: Take the desired volume of test samples/control sample and administer into the mice's stomach with a curved feeding needle.

1.2.1. Preliminary test

- *Exploration of maximal tolerated dose:*

10 mice were administered with 0.5 mL of test sample/mouse for 04 times (2 hours apart), equivalent to the dose of 100 mL of test sample/kg mice. The test was qualified if after 24 h and 7 days of monitoring, no test mice were died.

1.2.2. Real test

Real tests were conducted in 20 mice, divided into 02 groups including 01 control group and 01 test groups. The dose levels of test samples and the control sample were shown in Table 1.

Table 1. Acute toxicity test design

**Group** (*n* = 10)	**Dose** *(mL/kg mice of 20g),* The number of uses[a]	**Dose**		**Compared to the maximum expected dosage for humans[b]**
		mL/kg mice	*mL/kg mice*	
**Control** (C)	0.5 mL water x 04 times	----	----	----
**Test 1** (T1)	0.5 mL test sample (equipvalent to 2.5 x 10^9 CFU pff *Bacillus* probiotic spores) x 04 times	100 mL test sample/kg mice, equipvalent to 5 x 10^{11} CFU of *Bacillus* probiotic spores/kg	**99.99 g/kg**	**27.1 times**

[a]Each dose taken 2 hours apart

[b]The adult weight 50 kg in average, and the conversion factor from human to mouse dose is 12.3

- *Relevant information about the sample:*
- *The sample proportion: 0.9999 g/mL.*
- *Maximum expected dosage in humans per day: 3 ampoules/person/day (5 mL/ampoule)*

Monitoring schedule:

- Monitor the signs of poisoning: After suspension administered, the abnormal signs (in terms of physical condition, behavior, movement, eating, drinking, stool, urine,...) were monitored every 15 minutes within the first hour and gradually reduce frequency within the first 24 hours. Mouse's activity was continued to be monitored once a day for a period of 7 test days.

- Monitor the number of dead mice among the test and control groups.

- Monitor the level of food and water consumption during the test period.

- Monitor the weight at the following times: Just before administration; day 1, day 4 and day 7 after test sample administered in test groups compared to that of the control group (for the test groups with no dead mice were found).

1.3. Statistical analysis and data presentation

The data is presented as mean ± standard deviation (mean ± SD) and statistically analyzed using Student test with Prism software version 8.0 (Graph Pad Software). *P* value < 0.05 was considered to be statistically significant.

1.4. Results

1.4.1. Consumption of food and water in mice

- The control group: Normal eating and drinking.

- The test groups: After administration with the test sample and during 7 test days, no abnormalities were noticed. The level of food and water consumption was similar to that of the control group.

1.4.2. Observation of poisoning signs

- No sign of poisoning was observed in the test groups during the test period. Mice are healthy, agile, have smooth hair, eating and movement normally.

- No mice were died during the test.

1.4.3. Weight of mice

- The results of mice weight monitoring in the control and test groups are shown in Tables 2.

Table 2. Results of mice weight monitoring

**Group** (n = 10)	**Weight of mice** *(g)*			
	**Before**	**After 1 day**	**After 4 days**	**After 7 days**
**Control**	19.38 ± 0.70	20.70 ± 0.72	25.23 ± 0.58	29.47 ± 1.22
**Test 1**	19.40 ± 0.60	20.63 ± 0.55	25.35 ± 0.71	28.83 ± 1.24

- The data on the weight comparison of mice between the test group and the control group are shown in Table 3.

Table 3. Comparison of the weight between the control and test groups

**Group**	**Before test**		**After test**		**Gained weight** *(%)*	$P_{before-after}$
	**Weight of mouse** *(g)*	P_{before}	**Weight of mouse** *(g)*	P_{after}		
**Control** (C)	19.38 ± 0.70		29.47 ± 1.22		152.1	$P < 0.001$
**Test 1** (T1)	19.40 ± 0.60	$P_{T1-C} > 0.05$	28.83 ± 1.24	$P_{T1-C} > 0.05$	148.6	$P < 0.001$

Comments:

The results of monitoring the average weight of mice during the period of the 7-day test showed that:

- Before an oral administration: Average weight of mice in the test groups before entering the test did not have a statistically significant difference in comparison with the control group ($P_{(T\text{-}C)\ before}$ > 0.05).

- After 7 days of oral administration: The mice in both control and test groups gained weight. There was a significant difference in the weight of mice at day 7 in compared to mice at day 0 in each group ($P_{before\text{-}after} < 0.001$). There was no statistically significant difference in average weight after test in the test group compared to the control group ($P_{(T\text{-}C)\ after}$> 0.05).

1.4.4. Macroscopic observation results

At the end of the experiment, mice were dissected for macroscopic observation. The macroscopic observations showed that there was no difference in internal organs appearance (heart, liver, spleen, kidney, lung....) of the test group compared to the control group (Table 4).

Table 4. Results of macroscopic observation on mice

No.	Group	Macroscopic observations
1	Control	No abnormalities were observed
2	Test 1	No abnormalities compared to the control group

1.5. Conclusions

The samples of **3-*Bacillus* mixture ingredient for the products LiveSpo® Preg-mom and LiveSpo® Dia30** were tested for acute toxicity assay on Swiss white mice yielded the following results:

The test samples were administered into mice at a maximal dose level of 100 mL (equivalent to 5 x 10^{11} CFU of *Bacillus subtilis, Bacillus clausii* and *Bacillus coagulans* probiotic spores)/kg mice, no abnormalities were found in the test group compared to that of control group. Normal eating, drinking and activities were observed, and no dead mice were found. The macroscopic observation on the major internal organs of mice at the end of the experiment did not reveal any abnormalities compated to the control group.

The lethal dose for 50% of experimental animals (LD_{50}) was defined as greater than 100 mL (5 x 10^{11} CFU of spores) test sample (equivalent to 99.99 mg sample/kg mice). The defined non-lethal dose in experimental animals (LD_0) was 100 mL (5 x 10^{11} CFU of spores) test sample/kg mice. The dose that did not cause abnormal expression in the test animals was determined as 100 mL test sample (5 x 10^{11} CFU of spores)/kg mice (as 27.1-fold higher than converted humans' dose).

According to the toxicity classification of GHS (Globally Harmonized System of Classification and Labelling of Chemical, 2019), the substances/compounds with LD_{50} acute toxicity values greater than 5000 mg/kg mice intake orally in mice were considered to be low toxicity and unclassified. Based on the results obtained from this test, it can be concluded that the sample of **3-*Bacillus* mixture ingredient for the products LiveSpo® Preg-mom and LiveSpo® Dia30** has accute toxicity below the GHS classification threshold.

2. Assessment of the sub-acute toxicity

Implementation period: From 09/05/2023 to 21/06/2023.

2.1. Experimental animals

- Species: Mature and healthy New Zealand rabbit, both male and female. Female rabbits were not pregnant or lactating. The rabbits have not been subjected to any tests before. The rabbit's weights were in a range of 1.8 - 2.2 kg.

- Quantity: 21 rabbits were divided randomly into 03 groups (01 control group and 02 test groups), 07 rabbits in each group.

- Source of supply: Department of Livestock – Laboratory of Pharmacology - National Institute of Drug Quality Control.

- Care conditions: Each rabbit was housed individually in a cage, placed in a temperature and humidity-controlled room, food and water were supplied on demand. All experimental manipulations on animals were followed the procedures for care and use for experimental animals by the Laboratory of Pharmacology - Institute of National Drug Quality Control.

2.2. Procedures

2.2.1. Preparation of test sample

- Select the test dose level: The doses were selected based on the expected maximal dose for human is 03 ampoules (15 mL suspensions sample)/person/day,

equipvalent to 7.5 x 10^{10} CFU of *Bacillus* spores/person/day and conversion ratio of dose between rabbit and human is 3.1. The two dose levels were selected, including:

+ The dose, equivalent to the expected dose for humans: 0.93 mL of sample suspension/kg rabbit/day, equipvalent to 4.65 x 10^{9} CFU of *Bacillus* spores/kg rabbit/day.

+ The dose, equivalent to 5-fold higher than the expected dose for humans: 4.65 mL of sample suspension/kg rabbit/day, equipvalent to 2.33 x 10^{10} CFU of *Bacillus* spores/kg rabbit/day.

- Preparation of samples:

+ *Contro*l: Water.

+ *Suspension A* (*5-fold higher than the expected human dose*): Use the original sample suspension as provided.

+ *Suspension B (equivalent to the expected human dose)*: Dilute 20 mL of suspension A with sufficient water to make up to 100 mL suspension.

2.2.2. Experimental design

The experiment was conducted on 21 rabbits which were divided randomly into 03 groups with 07 rabbits in each group. The experimental design with dose levels is presented in Table 5.

Table 5. The test dose levels for sub-acute toxicity experiment.

**Groups**	**Number of test rabbits**	**Volume for oral administration** *(mL/kg rabbit)*	**Dosage** *(mL sample/kg rabbit)*
Control	07	4.65 mL water/kg rabbit	---
Test 1	07	4.65 mL suspension B/kg rabbit	0.93 mL/kg rabbit/ day, equipvalent to 4.65 x 10^{9} CFU of *Bacillus* spores/kg rabbit/day
Test 2	07	4.65 mL suspension A/kg rabbit	4.65 mL/kg rabbit/ day, equipvalent to 2.33 x 10^{10} CFU of *Bacillus* spores/kg rabbit/day

2.2.3. Monitoring and evaluation:

- The rabbits were monitored daily for status of food and water consumption, physical condition, activity, stool, urine and other abnormal manifestations (if any).

- The weight of rabbits at day 0, 7, 14, 21, 28 of administration and after 14 follow-up days since stopped administration were recorded.

- The hematological indicators related to hematopoietic function (number of red blood cells, white blood cells, platelets, hemoglobin, hematocrit); indicators related to liver function (AST, ALT, total protein, total bilirubin, cholesterol, albumin); indicators related to kidney function (creatinine, urea); glucose index at day 0, 14, 28 of administration and after 14 days since stopped administration were tested and collected. The results of the test groups and the control group were statistically analyzed.

- At the end of experiment, the rabbits were dissected for macroscopic observation of internal organs appearance, such as: heart, liver, kidney, lung, stomach, and intestine of all rabbits.

- 03 rabbits in each group were randomly taken to perform histopathological specimens of liver, kidney, small intestine, and large intestine to have the microscopic evaluation of the organs immediately after stopped administration.

2.2.4. Statistical analysis and data presentation

The data were presented as mean ± standard deviation (mean ± SD) and statistically analyzed using Student test to compare the difference of the same indicator between the control and the test groups.

2.3. Results

2.3.1. Rabbit status

During the test, all the rabbits moved normally, ate well, having bright eyes, dry feces, and smooth fur. Neither signs of abnormality in eating nor movement were found.

The rabbit weight monitoring during the test showed that:

- Before the experiment (before oral administration): The average weights of rabbits in the test groups were not different from the control group ($P_{(T1-C)\ before} > 0.05$; $P_{(T2-C)\ before} > 0.05$).

- After 28 days of oral administration: Rabbits gained weight steadily in both control and the two test groups. There was statistically difference in average weight at day 28 of experiment compared to that of the day before experiment in each group

(*$P_{before-after}$ < 0.01*). No significant difference in the average weights between the test groups and the control group was found (*$P_{(T1-C)\ after}$ > 0.05; $P_{(T2-C)\ after}$ > 0.05*).

- After 14 follow-up days since stopped administration: Rabbits were still healthy and gained weight well. No statistically difference in average weight between the test groups and the control group was found (*$P_{(T1-C)\ after}$ > 0.05; $P_{(T2-C)\ after}$ > 0.05*).

Table 6. Rabbit's weight monitoring result during sub-acute toxicity experiment

**Group (*n*=7)**	**Weight (kg)**					***P***
	Before the test (m_0)	***After 7 days (m_1)***	***After 14 days (m_2)***	***After 21 days (m_3)***	***After 28 days (m_4)***	
**Control (C)**	1.89 ± 0.09	2.01 ± 0.12	2.12 ± 0.12	2.24 ± 0.10	2.32 ± 0.11	$P_{before-after}$ < 0.01
% Compared to before test		106.4 %	112.3 %	118.9 %	123.0 %	
**Test1 (T1)**	1.86 ± 0.07	2.06 ± 0.21	2.15 ± 0.17	2.27 ± 0.16	2.34 ± 0.17	$P_{before-after}$ < 0.001<br>$P_{before(T1-C)}$ > 0.05<br>$P_{after(T1-C)}$ > 0.05
% Compared to before test		111.0 %	115.7 %	122.2 %	126.2 %	
**Test 2 (T2)**	1.98 ± 0.15	2.09 ± 0.21	2.23 ± 0.16	2.32 ± 0.17	2.40 ± 0.13	$P_{before-after}$ < 0.001<br>$P_{before(T2-C)}$ > 0.05<br>$P_{after(T2-C)}$ > 0.05
% Compared to before test		105.9 %	112.5 %	117.2 %	121.7 %	

Table 7. The rabbit's weight after 14 follow-up days since stopped administration.

**Group (*n* = 4)**	**Body weight (kg)**	***$P_{(T-C)}$** after 14 follow-up days*
Control (C)	2.45 ± 0.06	
Test 1 (T1)	2.44 ± 0.12	> 0.05
Test 2 (T2)	2.47 ± 0.18	> 0.05

2.3.2. Hematological indicators related to hematopoietic function.

a. Before the experiment (before oral administration):

Table 8. Hematological indicators before the experiment

Index	Control (*n* = 7)	Group T1 (*n* = 7)	*P* $_{(T1-C)}$	Group T2 (*n* = 7)	*P* $_{(T2-C)}$
**Red blood cell** *(x 10^{12}/l)*	5.6 ± 0.3	5.3 ± 0.5	> 0.05	5.8 ± 0.5	> 0.05
**White blood cell** *(x 10^{9}/l)*	5.8 ± 1.2	7.4 ± 2.9	> 0.05	6.8 ± 1.2	> 0.05
**Platelet** *(x 10^{9}/l)*	383.1 ± 121.0	340.1 ± 87.7	> 0.05	415.1 ± 140.5	> 0.05
**Hematocrit** *(%)*	37.4 ± 2.1	35.2 ± 2.8	> 0.05	38.4 ± 2.4	> 0.05
**Hemoglobin** *(g/dl)*	11.2 ± 0.6	10.5 ± 1.1	> 0.05	11.5 ± 1.0	> 0.05

b. Day 14 of oral administration

Table 9. Hematological indicators at day 14 of oral administration

Index	Control (*n* = 7)	Group T1 (*n* = 7)	*P* $_{(T1-C)}$	Group T2 (*n* = 7)	*P* $_{(T2-C)}$
**Red blood cell** *(x 10^{12}/l)*	5.5 ± 0.4	5.2 ± 0.3	> 0.05	5.7 ± 0.2	> 0.05
**White blood cell** *(x 10^{9}/l)*	6.6 ± 1.9	7.6 ± 1.7	> 0.05	7.6 ± 1.1	> 0.05
**Platelet** *(x 10^{9}/l)*	329.4 ± 88.0	393.3 ± 45.7	> 0.05	338.0 ± 37.6	> 0.05
**Hematocrit** *(%)*	36.1 ± 2.5	34.7 ± 1.5	> 0.05	37.6 ± 1.7	> 0.05
**Hemoglobin** *(g/dl)*	11.3 ± 0.7	10.7 ± 0.7	> 0.05	11.6 ± 0.4	> 0.05

c. Day 28 of oral administration

Table 10. Hematological indicators at day 28 of oral administration

**Index**	**Control (n = 7)**	**Group T1 (n = 7)**	***P*** *(T1-C)*	**Group T2 (n = 7)**	***P*** *(T2-C)*
**Red blood cell** *(x 10^{12}/l)*	5.6 ± 0.3	5.4 ± 0.3	> 0.05	5.6 ± 0.2	> 0.05
**White blood cell** *(x 10^{9}/l)*	8.1 ± 1.6	7.3 ± 1.0	> 0.05	8.6 ± 1.3	> 0.05
**Platelet** *(x 10^{9}/l)*	356.3 ± 55.9	384.9 ± 69.9	> 0.05	348.7 ± 99.0	> 0.05
**Hematocrit** *(%)*	36.8 ± 1.7	35.9 ± 1.7	> 0.05	36.9 ± 1.0	> 0.05
**Hemoglobin** *(g/dl)*	11.6 ± 0.6	11.1 ± 0.8	> 0.05	11.5 ± 0.5	> 0.05

d. After 14 follow-up days since stopped administration.

Table 11. Hematological indicators after 14 follow-up days since stopped administration.

**Index**	**Group (*n* = 4)**	**Group T1 (*n* = 4)**	***P*** *(T1-C)*	**Group T2 (*n* = 4)**	***P*** *(T2-C)*
**Red blood cell** *(x 10^{12}/l)*	5.7 ± 0.2	5.5 ± 0.4	> 0.05	5.6 ± 0.1	> 0.05
**White blood cell** *(x 10^{9}/l)*	9.7 ± 2.1	8.6 ± 1.0	> 0.05	9.1 ± 0.7	> 0.05
**Platelet** *(x 10^{9}/l)*	376.8 ± 76.1	406.5 ± 56.4	> 0.05	386.5 ± 58.1	> 0.05
**Hematocrit** *(%)*	37.5 ± 1.3	36.8 ± 2.4	> 0.05	36.4 ± 1.0	> 0.05
**Hemoglobin** *(g/dl)*	12.0 ± 0.4	11.8 ± 1.0	> 0.05	11.6 ± 0.5	> 0.05

Comments:

The results on hematological indicator showed that:

- Before the experiment: There was no statically significant difference in hematological indicators between the control and the test groups ($P_{(T\text{-}C)\ before} > 0.05$).

- After 14 days, 28 days of oral administration and after 14 follow-up days since stopped administration, the hematological indicators were not statically significant different between the test groups and the control group ($P_{(T\text{-}C)\ day\ 14} > 0.05$; $P_{(T\text{-}C) day\ 28} > 0.05$). $P_{(T\text{-}C)\ after\ 14\ follow\text{-}up\ days} > 0.05$).

2.3.3. Indicators related to liver function.

a. Before the experiment (before oral administration):

Table 12. Indicators related to liver function before the experiment.

Index	Control (*n* = 7)	Group T1 (*n* = 7)	*P* $_{(T1\text{-}C)}$	Group T2 (*n* = 7)	*P* $_{(T2\text{-}C)}$
**AST** *(U/l)*	44.0 ± 15.9	41.9 ± 18.8	> 0.05	39.4 ± 18.6	> 0.05
**ALT** *(U/l)*	53.0 ± 14.3	52.2 ± 14.8	> 0.05	49.6 ± 17.6	> 0.05
**Total Bilirubin** *(µmol/l)*	1.9 ± 0.7	2.0 ± 0.4	> 0.05	1.9 ± 0.6	> 0.05
**Total Protein** *(g/l)*	51.1 ± 3.1	53.0 ± 35	> 0.05	54.0 ± 5.8	> 0.05
**Albumin** *(g/dl)*	34.6 ± 1.6	34.6 ± 2.1	> 0.05	35.2 ± 4.8	> 0.05
**Cholesterol** *(mmol/l)*	2.4 ± 0.5	2.1 ± 0.6	> 0.05	2.7 ± 0.5	> 0.05

b. Day 14 of oral administration

Table 13. Indicators related to liver function at day 14 of administration.

Index	Control (*n* = 7)	Group T1 (*n* = 7)	*P* $_{(T1\text{-}C)}$	Group T2 (*n* = 7)	*P* $_{(T2\text{-}C)}$
**AST** *(U/l)*	34.0 ± 16.5	33.6 ± 16.3	> 0.05	38.5 ± 12.6	> 0.05
**ALT** *(U/l)*	54,2± 17,0	55.9 ± 20.7	> 0.05	54.7 ± 19.4	> 0.05

**Total Bilirubin** *(µmol/l)*	2.3 ± 0.3	2.3 ± 0.4	> 0.05	2.6 ± 0.5	> 0.05
**Total Protein** *(g/l)*	49.5 ± 3.3	51.7 ± 4.9	> 0.05	51.6 ± 2.7	> 0.05
**Albumin** *(g/dl)*	34.2 ± 1.9	34.3 ± 1.5	> 0.05	34.6 ± 2.2	> 0.05
**Cholesterol** *(mmol/l)*	2.4 ± 0.6	2.1 ± 0.8	> 0.05	2.3 ± 1.0	> 0.05

c. Day 28 of administration

Table 14. Indicators related to liver function on day 28 of administration.

**Index**	**Control (*n* = 7)**	**Group T1 (*n* = 7)**	***P*** $_{(T1-C)}$	**Group T2 (*n* = 7)**	***P*** $_{(T2-C)}$
**AST** *(U/l)*	35.6 ± 14.6	33.8 ± 10.4	> 0.05	34.4 ± 10.3	> 0.05
**ALT** *(U/l)*	58.6 ± 21.7	52.8 ± 15.3	> 0.05	52.6 ± 20.5	> 0.05
**Total Bilirubin** *(µmol/l)*	2.6 ± 0.3	2.6 ± 0.3	> 0.05	2.7 ± 0.3	> 0.05
**Total Protein** *(g/l)*	51.2 ± 1.7	51.1 ± 4.8	> 0.05	52.1 ± 2.0	> 0.05
**Albumin** *(g/dl)*	35.4 ± 1.9	35.8 ± 1.7	> 0.05	35.8 ± 0.9	> 0.05
**Cholesterol** *(mmol/l)*	2.6 ± 1.0	1.9 ± 0.5	> 0.05	1.8 ± 0.5	> 0.05

d. After 14 follow-up days since stopped administration.

Table 15. Indicators related to liver function after 14 follow-up days since stopped administration.

**Index**	**Control (n = 4)**	**Group T1 (n = 4)**	***P*** $_{(T1-C)}$	**Group T2 (n = 4)**	***P*** $_{(T2-C)}$
**AST** *(U/l)*	36.9 ± 6.2	32.8 ± 9.0	> 0.05	42.0 ± 15.2	> 0.05

**ALT** *(U/l)*	62.5 ± 16.7	58.0 ± 19.0	> 0.05	58.1 ± 16.7	> 0.05
**Total Bilirubin** *(µmol/l)*	2.5 ± 0.2	2.3 ± 0.2	> 0.05	2.5 ± 0.3	> 0.05
**Total Protein** *(g/l)*	54.6 ± 3.7	52.2 ± 3.6	> 0.05	55.2 ± 3.1	> 0.05
**Albumin** *(g/dl)*	35.8 ± 1.7	36.8 ± 2.0	> 0.05	36.5 ± 0.9	> 0.05
**Cholesterol** *(mmol/l)*	2.1 ± 0.7	1.6 ± 0.2	> 0.05	1.6 ± 0.5	> 0.05

Comments:

The results on liver function indicators showed that:

- Before the experiment: There was no statistically significant difference in the indicators related to liver function between the control group and the test groups ($P_{(T\text{-}C)\ before} > 0.05$).

- After 14 days, 28 days of oral administration and 14 follow-up days since stopped administration: There were no statistically significant differences in liver function related indices between the control and the test groups ($P_{(T\text{-}C)\ day\ 14} > 0.05$; $P_{(T\text{-}C)\ day\ 28} > 0.05$), $P_{(T\text{-}C)\ after\ 14\ follow\text{-}up\ days} > 0.05$).

2.3.4. Indicators related to kidney function.

a. Before the experiment (before oral administration):

Table 16. Indicators related to kidney function before the experiment.

Index	Control (*n* = 7)	Group T1 (*n* = 7)	$P_{(T1\text{-}C)}$	Group T2 (*n* = 7)	$P_{(T2\text{-}C)}$
**Urea** *(mmol/l)*	4.0 ± 1.0	4.4 ± 0.5	> 0.05	4.6 ± 0.8	> 0.05
**Creatinin** *(µmol/l)*	76.3 ± 9.1	79.8 ± 10.4	> 0.05	82.1 ± 15.2	> 0.05

b. Day 14 of oral administration

Table 17. Indicators related to kidney function at day 14 of administration.

Index	Control (*n* = 7)	Group T1 (*n* = 7)	$P_{(T1\text{-}C)}$	Group T2 (*n* = 7)	$P_{(T2\text{-}C)}$

**Urea** *(mmol/l)*	4.3 ± 0.5	3.8 ± 1.0	> 0.05	4.7 ± 1.1	> 0.05
**Creatinin** *(µmol/l)*	92.3 ± 17.7	95.9 ± 16.3	> 0.05	100.0 ± 12.0	> 0.05

c. Day 28 of oral administration

Table 18. Indicators related to kidney function at day 28 of administration.

Index	Control (n = 7)	Group T1 (n = 7)	$P_{(T1-C)}$	Group T2 (n = 7)	$P_{(T2-C)}$
**Urea** *(mmol/l)*	5.3 ± 0.9	4.6 ± 0.7	> 0.05	4.7 ± 0.5	> 0.05
**Creatinin** *(µmol/l)*	94.8 ± 14.8	93.2 ± 13.4	> 0.05	94.1 ± 14.7	> 0.05

d. After 14 follow-up days since stopped administration.

Table 19. Indicators related to kidney function after 14 follow-up days since stopped administration.

Index	Control (n = 4)	Group T1 (n = 4)	$P_{(T1-C)}$	Group T2 (n = 4)	$P_{(T2-C)}$
**Urea** *(mmol/l)*	5.5 ± 0.4	4.9 ± 0.4	> 0.05	5.1 ± 1.1	> 0.05
**Creatinin** *(µmol/l)*	99.3 ± 6.7	93.0 ± 7.6	> 0.05	98.5 ± 17.8	> 0.05

Comments:

Results on urea and creatinine tests showed that:

- Before the experiment: There was no statistically significant difference in the indicators related to kidney function between the control and the 2 test groups ($P_{(T-C)\ before} > 0.05$).

- After 14 days, 28 days of oral administration and after 14 follow-up days since stopped administration: There was no statistically significant difference in the indicators related to kidney function between the control and the test groups ($P_{(T-C)\ day\ 14} > 0.05$; $P_{(T-C)\ day\ 28} > 0.05$); $P_{(T-C)\ after\ 14\ follow\text{-}up\ days} > 0.05$).

2.3.5. Blood glucose index

a. Before the experiment (before oral administration):

Table 20. Glucose index before the experiment

Index	Control (*n* = 7)	Group T1 (*n* = 7)	$P_{(T1-C)}$	Group T2 (*n* = 7)	$P_{(T2-C)}$
**Glucose** *(mmol/l)*	6.7 ± 1.2	6.6 ± 0.6	> 0.05	6.1 ± 1.1	> 0.05

b. Day 14 of oral administrations

Table 21. Glucose index at day 14 of oral administration

Index	Control (*n* = 7)	Group T1 (*n* = 7)	$P_{(T1-C)}$	Group T2 (*n* = 7)	$P_{(T2-C)}$
**Glucose** *(mmol/l)*	5.9 ± 1.0	5.3 ± 0.9	> 0.05	5.4 ± 1.4	> 0.05

c. Day 28 of oral administrations

Table 22. Glucose index at day 28 of oral administrations

Index	Control (*n* = 7)	Group T1 (*n* = 7)	$P_{(T1-C)}$	Group T2 (*n* = 7)	$P_{(T2-C)}$
**Glucose** *(mmol/l)*	4.4 ± 1.1	4.1 ± 0.7	> 0.05	4.5 ± 0.8	> 0.05

d. After 14 follow-up days since stopped administration.

Table 23. Glucose index after 14 follow-up days since stopped administration

Index	Control (*n* = 4)	Group T1 (*n* = 4)	$P_{(T1-C)}$	Group T2 (*n* = 4)	$P_{(T2-C)}$
**Glucose** *(mmol/l)*	4.9 ± 0.3	5.5 ± 0.7	> 0.05	4.9 ± 0.9	> 0.05

Comments

The data on the blood glucose test showed that:

- Before the experiment: There was no statistically significant difference in glucose index between the control group and the 2 test groups ($P_{(T-C)\ before} > 0.05$).

- After 14 days, 28 days of oral administration and after 14 follow-up days since stopped administration: There was no statistically significant difference in glucose index between the control and the 2 test groups ($P_{(T-C)\ day\ 14} > 0.05$; $P_{(T-C)\ day\ 28} > 0.05$); $P_{(T-C)\ after\ 14\ follow\text{-}up\ days} > 0.05$).

2.3.6. Macroscopic observation

The results of macroscopic observation for internal organs appearance showed that, after the experiment, there were no abnormal appearances in color of heart, lung, liver, spleen, kidney, stomach, intestines of rabbits in test groups compared to control group.

Table 24. Macroscopic images of internal organs

**Macroscopic images**		
Control	***Test 1 (low dose)***	***Test 2 (high dose)***
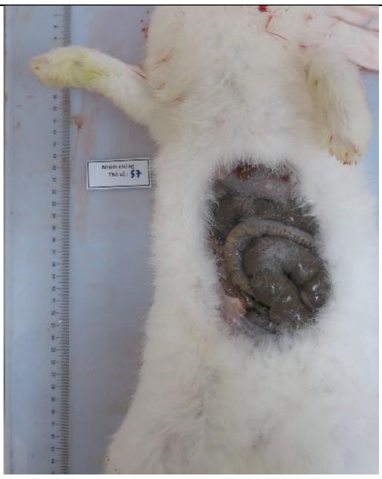	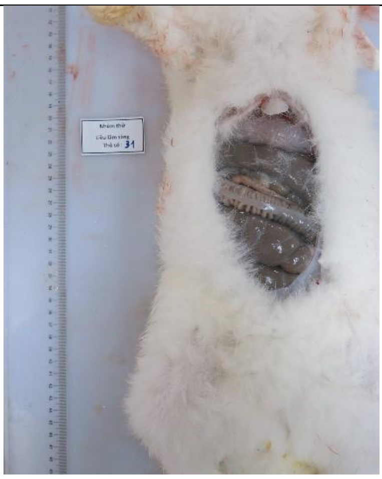	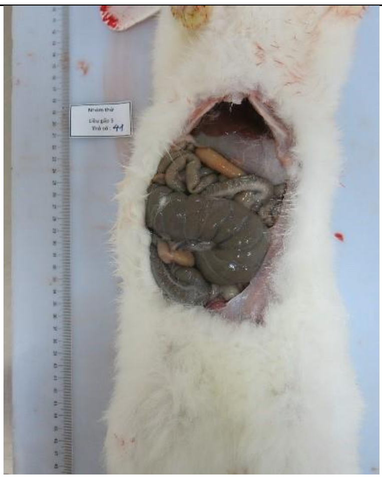

2.3.7. Microscopic observation

Liver, kidney, small intestine, and colon specimens were fixed with 10% formalin, stained with Hematoxylin Eosin (HE) and Perioric Acid Shiff (PAS) staining solution and observed under optical microscope.

The microscopic observation was performed at the Department of Pathophysiology Anatomy - Hanoi Medical University. The results showed that the entire test rabbits showed unscathed liver, kidney, small intestine, and colon. Morphological anatomy was within normal limits. There were no abnormal symptoms related to the test groups using the two different dose levels compared with the control group.

The detailed microscopic analysis was as follows:

Liver:

- Hepatocytes: No injury.
- Liver capillaries: Normal.
- Central vein: Normal.

- Portal hepatic: Normal.
- Interstitial tissue: Normal.

Conclusion: Liver tissue morphology was normal, there was no apparent injury

Kidney:

- Glomerulus (capillary, mesangium, Baomann capsule): Normal.
- Hepatic duct/ Renal columns: Normal.
- Renal pelvis: Normal
- Interstitial tissue: Normal.

Conclusion: Kidney tissue is normal and there was no apparent injury

Small intestine:

- Mucous membrane (villi, covering epithelium, striated cells, goblet cells, Lieberkühn glands, stromal tissue, mucosal muscles): Normal.
- Submucosa: Normal.
- Muscle layer: Normal.
- Serosa: Normal.

Conclusion: The morphology and histopathology of the small intestine were normal.

Colon:

- Mucous membrane (covering epithelium, goblet cells, Lieberkuhn glands, stromal tissue, mucosal muscles): Normal.
- Submucosa: Normal.
- Muscle layer: Normal.
- Serosa: Normal.

Conclusion: The morphology and histopathology of the colon were normal.

Table 25. The histopathological anatomy images under microscope of liver, kidney, small intestine, colon (HE stain x400)

No	*Group*	*Liver tissue*	*Kidney tissue*	*Small intestine tissue*	*Colon tissue*
1	*Control*	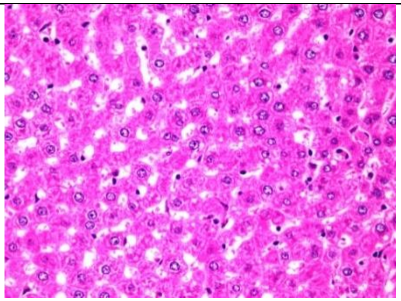 Normal and unscathed liver tissue morphology.	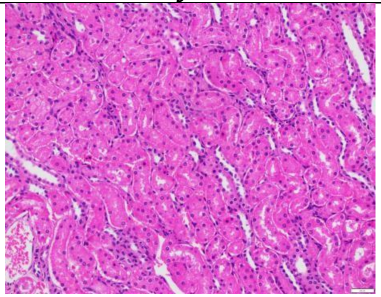 Normal and unscathed kidney tissue morphology	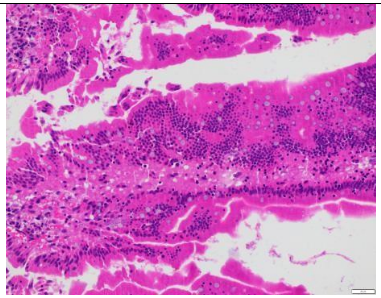 Normal and unscathed small intestine tissue morphology	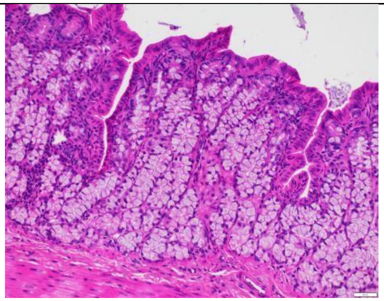 Normal and unscathed colon tissue morphology
2	*Test 1 (low dose)*	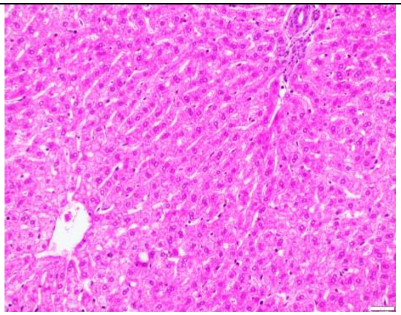 Normal and unscathed liver tissue morphology.	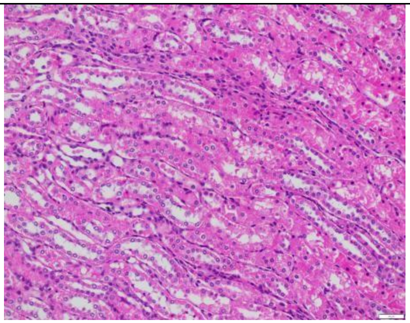 Normal and unscathed kidney tissue morphology	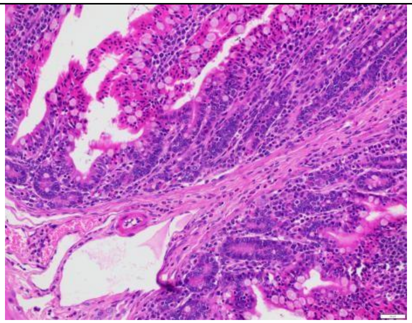 Normal and unscathed small intestine tissue morphology	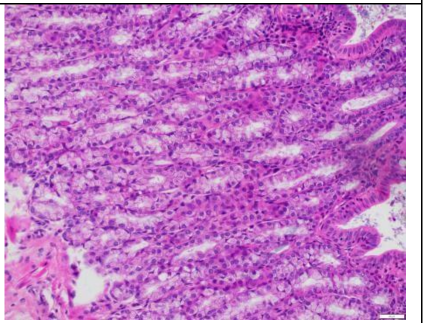 Normal and unscathed colon tissue morphology
3	*Test 2 (high dose)*	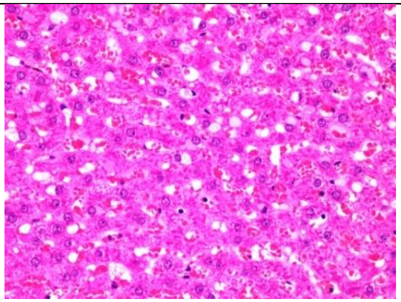 Normal and unscathed liver tissue morphology	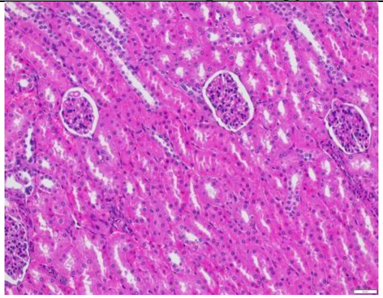 Normal and unscathed kidney tissue morphology	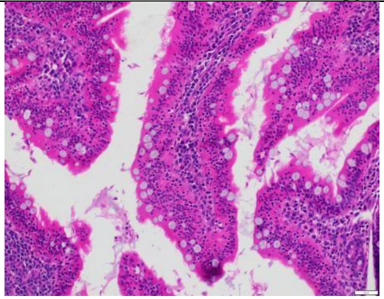 Normal and unscathed small intestine tissue morphology	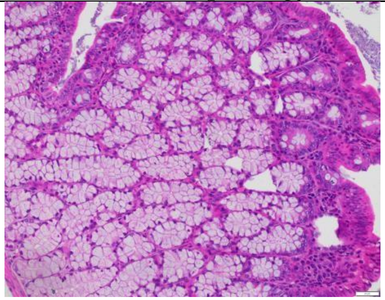 Normal and unscathed colon tissue morphology

Table 26. The histopathological anatomy images under microscope of liver, kidney, small intestine, colon (PAS stain x400)

No	*Group*	*Liver tissue*	*Kidney tissue*	*Small intestine tissue*	*Colon tissue*
1	*Control*	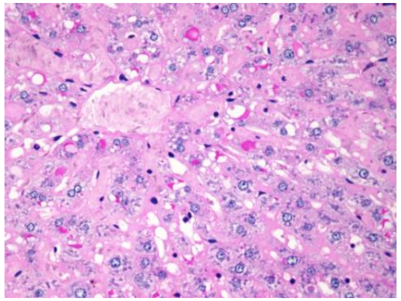 Normal and unscathed liver tissue morphology	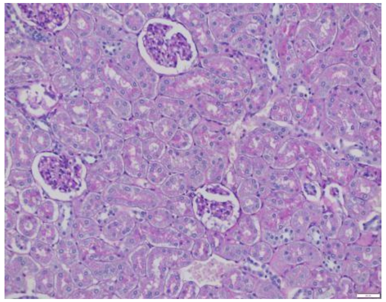 Normal and unscathed kidney tissue morphology	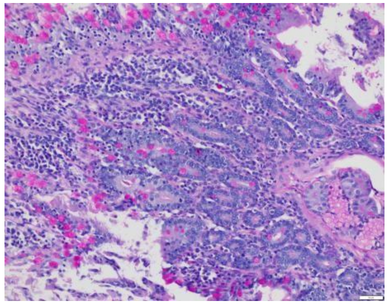 Normal and unscathed small intestine tissue morphology	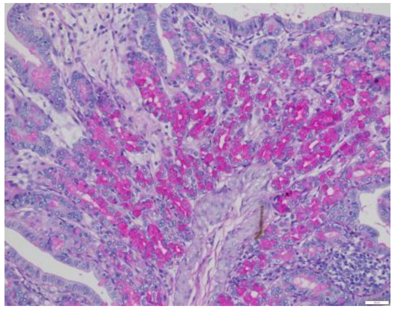 Normal and unscathed colon tissue morphology
2	*Test 1 (low dose)*	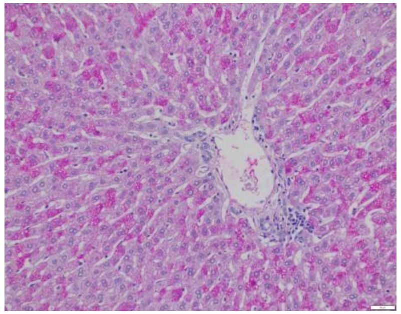 Normal and unscathed liver tissue morphology	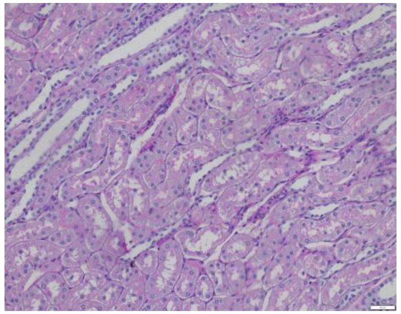 Normal and unscathed kidney tissue morphology	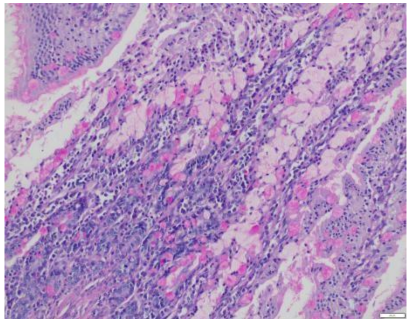 Normal and unscathed small intestine tissue morphology	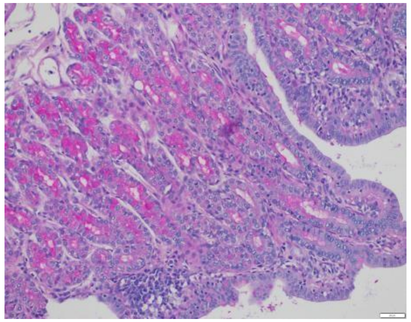 Normal and unscathed colon tissue morphology

3	***Test 2*** ***(high dose)***	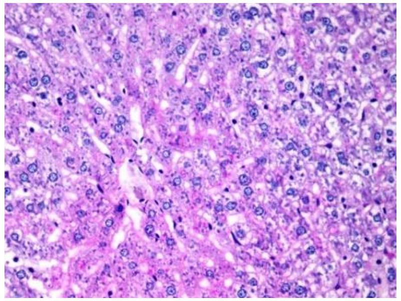 Normal and unscathed liver tissue morphology	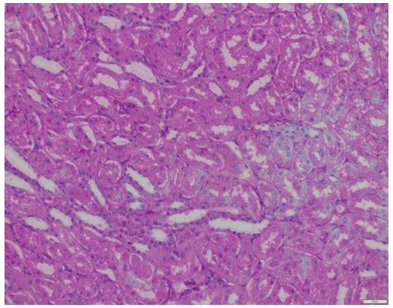 Normal and unscathed kidney tissue morphology.	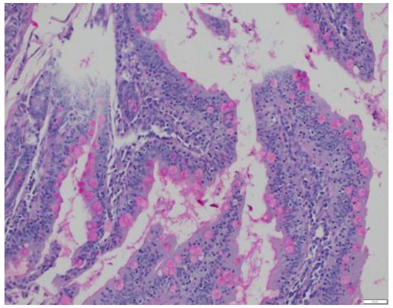 Normal and unscathed small intestine tissue morphology	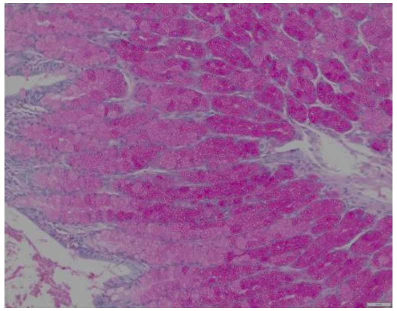 Normal and unscathed colon tissue morphology

2.4. Conclusions

The samples of **3-*Bacillus* mixture ingredient for the products LiveSpo® Preg-mom and LiveSpo® Dia30** were tested for sub-acute toxicity assay on rabbit yielded the following results:

After 28 days of continuous oral administration of test suspension at the two different doses of 0.93 mL (4.65 x 10^9 CFU)/kg rabbit/day (equivalent to the maximum dose for humans of 3 ampoules/person/day or 7.5 x 10^{10} CFU spores of *Bacillus subtilis, Bacillus clausii* and *Bacillus coagulans*/person/day) and 4.65 mL (2.33 x 10^{10} CFU/kg rabbit/day (5-fold higher than the maximum human dose, equivalent to 3.75 x 10^{11} CFU of *Bacillus subtilis, Bacillus clausii* and *Bacillus coagulans* spores/person/day), the weight, physical condition, and movement of the test rabbits were not affected. The rabbits were healthy and gained weight.

The biochemical indicators for liver and kidney functions (activities of enzymes AST, ALT, total protein, total bilirubin, cholesterol, albumin, glucose, urea, creatinine) and hematological indicators (red blood cells, hemoglobin, hematocrit, leukocytes, platelets) were not statistically significant different at the points of before the experimental, day 14, day 28 of experiment and after 14 follow-up days since stopped administration in comparison between the two test groups and the control group.

Macroscopic observation was not found any abnormalities in the heart, lung, liver, spleen, kidney, stomach, and intestines of the test rabbits. Microscopic observation did not show histopathological lesions of liver, kidney, small intestine, colon between the two test groups and the control group.

Ha Noi, July 19th, 2023.

Certified by the Head of Organization	LABORATORY OF PHARMACOLOGY
**(signed and stamped)**	**(Signed)**
**Doan Cao Son**	**Dr. Nguyen Thi Lien**

VIỆN KIỂM NGHIỆM THUỐC TRUNG ƯƠNG

KHOA DƯỢC LÝ

KẾT QUẢ THỬ NGHIỆM
ĐỘC TÍNH CẤP VÀ BÁN TRƯỜNG DIỄN

(Kết quả thử nghiệm chỉ có giá trị với mẫu đem thử)

THÔNG TIN CHUNG

Tên mẫu:	Nguyên liệu 3 – Bacillus Mixture cho sản phẩm probiotic LiveSpo Preg-Mom và LiveSpo Dia30
Nhà sản xuất:	Công ty TNHH LiveSpo Pharma
Lô sản xuất:	1223A
Ngày sản xuất:	01/02/2023
Hạn dùng:	31/01/2025
Nơi gửi mẫu:	Công ty TNHH LiveSpo Pharma
Chỉ tiêu thử:	Độc tính cấp và Bán trường diễn
Tài liệu thử:	1. Phương pháp xác định độc tính của thuốc – Nhà xuất bản Y học 2014<br>2. OECD guidelines for testing of chemicals. Repeated dose 28 - days Oral Toxicity study in Rodents OECD 407, 2008.
Công thức bào chế trên nhãn:	*B. subtilis, B. clausii và B. coagulans* nồng độ 5 tỷ CFU/ml dạng nguyên liệu cho sản phẩm probiotic LiveSpo Preg-Mom và LiveSpo Dia30
Tình trạng mẫu khi mở niêm phong để thử nghiệm:	Mẫu thử được đóng trong ống nhựa màu trắng, mỗi ống 5 mL mẫu thử
Nơi thực hiện:	Khoa Dược lý - Viện Kiểm nghiệm Thuốc Trung Ương
Địa chỉ	Tam Hiệp, Thanh Trì, Hà Nội
Trụ sở	48 Hai Bà Trưng, Hà Nội

KẾT QUẢ THỬ NGHIỆM

1. Thử nghiệm độc tính cấp

Thời gian thực hiện: 24/05/2023 đến ngày 31/05/2023

1.1. Động vật thí nghiệm

- Loài: chuột nhắt trắng giống Swiss.
- Cân nặng: 18 - 22 g.
- Số lượng: 30 con.
- Nguồn gốc cung cấp: Viện Vệ sinh Dịch tễ Trung ương.
- Điều kiện chăm sóc: Chuột được nuôi 6 - 8 con một chuồng trong phòng nuôi có kiểm soát nhiệt độ và độ ẩm thích hợp với thức ăn và nước uống theo nhu cầu. Tất cả các thao tác trên động vật thí nghiệm đều được tuân theo các quy trình về chăm sóc và sử dụng động vật thí nghiệm của Khoa Dược lý - Viện Kiểm nghiệm thuốc TW.

1.2. Thực nghiệm

- Chuột được nhịn ăn 3 - 4 giờ trước khi thử nghiệm, nước uống theo nhu cầu. Kiểm tra cân nặng trước khi thử nghiệm. Chuột đạt các yêu cầu về cân nặng được đưa vào thử nghiệm.
- Cách xử lý và chuẩn bị mẫu thử: Dùng nguyên mẫu.
- Mẫu chứng: nước đun sôi để nguội
- Cách cho chuột uống: Lấy thể tích mẫu thử/mẫu chứng theo quy định đưa thẳng vào dạ dày chuột bằng kim cong đầu tù.

1.2.1. Thử sơ bộ

- Thăm dò ở mức liều dung nạp tối đa:

Dùng 10 chuột, cho mỗi chuột uống 0,5 mL mẫu thử x 4 lần (mỗi lần cách nhau 2 giờ) tương đương mức liều 100,0 mL mẫu thử/kg chuột. Sau 24 giờ theo dõi, không có chuột thí nghiệm bị chết. Theo dõi 7 ngày, không có chuột thí nghiệm bị chết.

1.2.2. Thử nghiệm chính thức

Tiến hành thử nghiệm chính thức trên 20 chuột, chia thành 2 nhóm gồm 1 nhóm chứng và 1 nhóm thử. Các nhóm chuột được dùng mẫu thử và mẫu chứng theo mức liều được trình bày ở Bảng 1.

Bảng 1. Bố trí thử nghiệm thử độc tính cấp

Nhóm *(n = 10)*	Liều dùng (mL/chuột 20g), số lần dùng[a]	Liều dùng		So với liều dùng tối đa dự kiến trên người[b]
		mL/kg chuột	mL/kg chuột	
**Chứng** (C)	0,50 mL nước x 4 lần	----	----	----
**Thử 1** (T1)	0,50 mL mẫu thử (tương đương với 2,5 tỷ CFU bào tử lợi khuẩn *Bacillus*) x 4 lần	100,0 mL mẫu thử/kg, tương đương 5 x 10^{11} CFU bào tử lợi khuẩn *Bacillus*/kg	**99,99 g/kg**	**27,1 lần**

[a] Mỗi lần uống cách nhau 2 giờ

[b] Người cân nặng 50 kg, hệ số quy đổi liều từ người sang chuột là 12,3.

** Thông tin liên quan của mẫu thử*

- Tỷ trọng của mẫu thử: 0,9999 g/mL

- Liều dùng tối đa dự kiến trên người/ngày: 3 ống/người/ngày (5 mL/ống)

Lịch theo dõi

- Theo dõi biểu hiện ngộ độc: Sau khi uống hỗn dịch thử theo dõi các dấu hiệu bất thường (về thể trạng, hành vi, vận động, tình trạng ăn, uống, phân, nước tiểu…) với tần suất khoảng 15 phút 1 lần trong vòng 1 giờ đầu và giãn dần tần suất trong vòng 24 giờ đầu. Tiếp tục theo dõi hoạt động của động vật thí nghiệm mỗi ngày 1 lần trong thời gian 7 ngày sau khi uống.

- Theo dõi số chuột chết trong các nhóm thử và nhóm chứng.

- Theo dõi mức độ tiêu thụ thức ăn, nước uống trong thời gian thử nghiệm.

- Theo dõi khối lượng chuột tại các thời điểm ngay trước khi uống, 1 ngày, 4 ngày và 7 ngày sau khi uống mẫu thử so với nhóm chứng (với các nhóm thử không có chuột thí nghiệm bị chết).

1.3. Trình bày và xử lý số liệu

Số liệu được trình bày dưới dạng giá trị trung bình cộng trừ độ lệch chuẩn (mean ± SD) và được xử lý thống kê bằng trắc nghiệm Student sử dụng phần mềm Prism phiên bản 8.0 (Graph Pad Software). Giá trị $P < 0,05$ được coi là có ý nghĩa thống kê.

1.4. Kết quả

1.4.1. Tiêu thụ thức ăn và nước uống của chuột

- Nhóm chứng: Ăn uống bình thường.

- Các nhóm thử: Sau khi uống thuốc và trong 7 ngày theo dõi nhóm thử, không nhận thấy có biểu hiện gì khác thường. Mức độ tiêu thụ thức ăn nước uống tương đương với nhóm chứng.

1.4.2. Quan sát dấu hiệu ngộ độc

- Không nhận thấy có biểu hiện ngộ độc ở các nhóm thử trong thời gian theo dõi. Chuột khỏe mạnh, nhanh nhẹn, lông mượt, ăn uống, vận động bình thường.

- Không có chuột chết trong quá trình thử nghiệm.

1.4.3. Khối lượng cơ thể chuột

- Kết quả theo dõi khối lượng của chuột ở nhóm chứng và các nhóm thử được thể hiện trong các Bảng 2.

Bảng 2. Kết quả theo dõi khối lượng chuột

Nhóm (n = 10)	Khối lượng chuột *(g)*			
	Trước thử nghiệm	Sau 1 ngày	Sau 4 ngày	Sau 7 ngày
Nhóm chứng	19,38 ± 0,70	20,70 ± 0,72	25,23 ± 0,58	29,47 ± 1,22
Nhóm thử 1	19,40 ± 0,60	20,63 ± 0,55	25,35 ± 0,71	28,83 ± 1,24

- Kết quả so sánh cân nặng của chuột thí nghiệm giữa các nhóm thử và nhóm chứng được thể hiện ở Bảng 3.

Bảng 3. Bảng so sánh cân nặng giữa nhóm chứng và các nhóm thử

Nhóm	Trước thử nghiệm		Sau thử nghiệm		Tăng khối lượng *(%)*	$P_{trước\text{-}sau}$
	Khối lượng chuột *(g)*	$P_{trước}$	Khối lượng chuột *(g)*	P_{sau}		
Chứng (C)	19,38 ± 0,70		29,47 ± 1,22		152,1	$P < 0,001$
Thử 1 (T1)	19,40 ± 0,60	$P_{T1\text{-}C} > 0,05$	28,83 ± 1,24	$P_{T1\text{-}C} > 0,05$	148,6	$P < 0,001$

Nhận xét:

Kết quả theo dõi khối lượng trung bình của chuột trong quá trình thử nghiệm 7 ngày cho thấy:

- Trước khi uống mẫu thử: Khối lượng trung bình của chuột ở nhóm thử trước khi đưa vào thử nghiệm không có sự khác biệt có ý nghĩa thống kê so với nhóm chứng ($P_{(T-C)\ trước} > 0,05$).

- Sau uống mẫu thử 7 ngày: Chuột thí nghiệm ở nhóm chứng và nhóm thử đều tăng cân. Có sự khác biệt đáng kể về khối lượng của chuột khi so sánh với trước thử nghiệm trong mỗi nhóm ($P_{trước-sau} < 0,001$). Không có sự khác biệt có ý nghĩa thống kê về cân nặng trung bình sau thử nghiệm giữa nhóm thử và nhóm chứng ($P_{(T-C)\ sau} > 0,05$).

1.4.4. Kết quả quan sát đại thể

Sau khi kết thúc thử nghiệm, chuột được mổ để quan sát đại thể. Kết quả quan sát đại thể cho thấy không có sự khác biệt ở các cơ quan nội tạng (tim, gan, lách, thận, phổi,...) so với nhóm chứng (Bảng 4).

Bảng 4. Kết quả mổ quan sát đại thể

STT	Nhóm	Các phát hiện đại thể
1	Chứng	Không có biểu hiện bất thường quan sát được
2	Thử 1	Không có sự bất thường so với nhóm chứng

1.5. Kết luận

Mẫu thử **Nguyên liệu 3 – Bacillus Mixture cho sản phẩm LiveSpo Preg-Mom và LiveSpo Dia30** gửi tới yêu cầu thử độc tính cấp trên chuột nhắt trắng có kết quả như sau:

Cho chuột uống mẫu thử với mức liều tối đa có thể cho uống là 100,0 mL mẫu thử (chứa 5×10^{11} CFU bào tử lợi khuẩn *Bacillus subtilis, Bacillus clausii và Bacillus coagulans)* /kg chuột, không nhận thấy có biểu hiện bất thường so với nhóm chứng. Chuột ăn uống, hoạt động bình thường, không có chuột chết. Quan sát đại thể các cơ quan nội tạng của chuột sau khi kết thúc thí nghiệm không nhận thấy bất thường so với nhóm chứng.

Xác định được liều gây chết 50% động vật thí nghiệm (LD_{50}) lớn hơn 100,0 mL (5×10^{11} CFU) mẫu thử/kg chuột (tương đương 99,99 g mẫu thử/ kg chuột). Xác định được

liều không gây chết động vật thí nghiệm (LD_0) là 100,0 mL (5 x 10^{11} CFU) mẫu thử/kg chuột. Xác định được liều không gây biểu hiện bất thường trên động vật thí nghiệm là 100,0 mL (5 x 10^{11} CFU) mẫu thử/kg chuột (cao gấp 27,1 lần liều dùng qui đổi trên người).

Theo phân loại độc tính của GHS (Globally Harmonized System of Classification and Labelling of Chemicals, 2021), những chất/hợp chất có giá trị độc tính cấp LD_{50} lớn hơn 5000 mg/kg chuột theo đường uống được coi là độc tính thấp và không phân loại (unclassified). Dựa trên kết quả thu được của thử nghiệm này có thể kết luận mẫu thử Nguyên liệu 3- Bacillus Mixture có độc tính thấp dưới ngưỡng phân loại của GHS.

2. Thử nghiệm độc tính bán trường diễn

Thời gian thực hiện: Từ 09/05/2023 đến 21/06/2023

2.1. Động vật thí nghiệm

- Loài, giống: Thỏ Newzealand trưởng thành cả hai giống đực và cái, khỏe mạnh, thỏ cái không mang thai hoặc cho con bú, chưa trải qua bất kỳ thử nghiệm nào trước đó, cân nặng khoảng 1,8 – 2,2 kg.

- Số lượng: 21 con được chia ngẫu nhiên thành 3 nhóm thử nghiệm (1 nhóm chứng và 2 nhóm uống mẫu thử), mỗi nhóm 07 con.

- Nguồn gốc: Bộ phận chăn nuôi - Khoa Dược lý - Viện Kiểm nghiệm thuốc Trung Ương.

- Điều kiện chăm sóc: Thỏ được nuôi mỗi con một lồng trong phòng nuôi có kiểm soát nhiệt độ và độ ẩm thích hợp với thức ăn và nước uống theo nhu cầu. Tất cả các thao tác trên động vật thí nghiệm đều được tuân theo các quy trình về chăm sóc và sử dụng động vật thí nghiệm của Khoa Dược lý – Viện Kiểm nghiệm thuốc Trung Ương.

2.2. Tiến hành

2.2.1. Chuẩn bị mẫu thử

- Lựa chọn mức liều thử nghiệm: Dựa trên liều tối đa dự kiến dùng trên người là 3 ống (15 mL hỗn dịch mẫu thử)/người/ngày, tương đương 75 tỷ bào tử lợi khuẩn *Bacillus*/người/ngày và sử dụng hệ số chuyển đổi liều giữa thỏ và người là 3,1 để lựa chọn 2 mức liều thử nghiệm là:

+ Liều tương ứng với mức liều dự kiến cho người: 0,93 mL hỗn dịch mẫu thử/kg thỏ/ngày, tương đương với 4,65 tỷ bào tử lợi khuẩn *Bacillus*/kg thỏ/ngày.

+ Liều cao gấp 5 lần liều dự kiến cho người: 4,65 mL hỗn dịch mẫu thử/kg thỏ/ngày, tương đương với 23,25 tỷ bào tử lợi khuẩn *Bacillus*/kg thỏ/ngày.

- Cách xử lý và chuẩn bị mẫu thử:

+ *Mẫu đối chứng*: Nước

+ *Hỗn dịch A (liều cao gấp 5 liều dự kiến cho người):* Dùng nguyên mẫu

+ *Hỗn dịch B (liều tương ứng với liều dự kiến cho người):* Pha loãng 20 ml hỗn dịch A với nước vừa đủ 100 ml.

2.2.2. Bố trí thử nghiệm

Thử nghiệm được tiến hành trên 21 thỏ, chia thành 3 nhóm: mỗi nhóm 07 con. Bố trí thử nghiệm và thử với các mức liều theo Bảng 5.

Bảng 5. Các mức liều thử nghiệm bán trường diễn trên thỏ

**Nhóm**	**Số thỏ thí nghiệm**	**Thể tích cho uống** *(mL/kg thỏ)*	**Liều dùng** *(mL mẫu thử/kg thỏ)*
Chứng	07	4,65 mL nước/kg thỏ	---
Thử 1 (T1)	07	4,65 mL hỗn dịch B/kg thỏ	0,93 mL/kg thỏ/ngày, tương đương 4,65 tỷ bào tử lợi khuẩn/kg thỏ/ngày
Thử 2 (T2)	07	4,65 mL hỗn dịch A/kg thỏ	4,65 mL/kg thỏ/ngày, tương đương 23,25 tỷ bào tử lợi khuẩn/kg/ngày

2.2.3. Theo dõi và đánh giá

- Theo dõi thỏ hàng ngày về mức độ tiêu thụ thức ăn, nước uống, thể trạng và vận động, tình trạng phân, nước tiểu, các biểu hiện bất thường (nếu có) của thỏ.

- Xác định cân nặng của thỏ tại các thời điểm 0, 7, 14, 21, 28 ngày uống mẫu thử và 14 ngày sau khi ngừng uống mẫu thử.

- Xét nghiệm các chỉ số huyết học liên quan tới chức năng tạo máu (số lượng hồng cầu, bạch cầu, tiểu cầu, hemoglobin, hematocrit), các chỉ số liên quan tới chức năng gan (AST, ALT, protein toàn phần, bilirubin toàn phần, cholesterol, albumin), các chỉ số liên quan tới chức năng thận (creatinin, urea), chỉ số glucose tại các thời điểm 0, 14, 28 ngày uống mẫu thử và 14 ngày sau khi ngừng uống mẫu thử. So sánh kết quả của nhóm thử và nhóm chứng theo phương pháp thống kê.

- Sau thử nghiệm động vật được mổ để quan sát đại thể các tổ chức tim, gan, thận, phổi, dạ dày, ruột của tất cả các thỏ.

- Lấy ngẫu nhiên 03 thỏ/nhóm, tiến hành làm tiêu bản giải phẫu mô bệnh học gan, thận, ruột non, ruột già để đánh giá vi thể các tổ chức trên ngay sau khi ngừng uống mẫu thử.

2.2.4. Trình bày và xử lý số liệu

Số liệu thực nghiệm được trình bày dưới dạng giá trị trung bình cộng trừ độ lệch chuẩn (mean ± SD) và được xử lý thống kê bằng trắc nghiệm Student để so sánh sự khác nhau của cùng một chỉ số giữa nhóm chứng và nhóm thử.

2.3. Kết quả

2.3.1. Tình trạng thỏ

Trong thời gian thử nghiệm, tất cả các thỏ đều hoạt động bình thường, ăn uống tốt, mắt sáng, lông mượt, phân khô. Không có biểu hiện bất thường về thể trạng, ăn uống cũng như vận động.

Theo dõi cân nặng thỏ trong quá trình thử nghiệm cho thấy:

- Trước thử nghiệm (trước khi uống mẫu thử): Cân nặng trung bình của thỏ ở các nhóm thử trước khi đưa vào thử nghiệm không có sự khác biệt so với nhóm chứng ($P_{trước(T1-C)} > 0,05$; $P_{trước(T2-C)} > 0,05$).

- Sau 28 ngày uống mẫu thử: Thỏ ở nhóm chứng và hai nhóm thử đều tăng cân ở mỗi thời điểm đánh giá. Có sự khác biệt có ý nghĩa về cân nặng của thỏ khi so sánh sau 28 ngày thử nghiệm với trước thử nghiệm trong mỗi nhóm ($P_{trước-sau} < 0,01$). Không có sự khác biệt có ý nghĩa về cân nặng trung bình giữa nhóm thử so với nhóm chứng ($P_{sau(T1-C)} > 0,05$; $P_{sau(T2-C)} > 0,05$).

- Sau 14 ngày ngừng uống mẫu thử: Thỏ khỏe mạnh tăng cân tốt, không có sự khác biệt có ý nghĩa về cân nặng trung bình giữa hai nhóm thử so với nhóm chứng ($P_{sau(T1-C)} > 0,05$; $P_{sau(T2-C)} > 0,05$).

Bảng 6. Kết quả theo dõi cân nặng của thỏ trong thời gian uống mẫu thử

Nhóm (n = 7)	Khối lượng cơ thể (kg)					P
	TrướcTN (m_0)	*Sau 7 ngày (m_1)*	*Sau 14 ngày (m_2)*	*Sau 21 ngày(m_3)*	*Sau 28 ngày (m_4)*	
**Chứng (C)**	1,89 ± 0,09	2,01 ± 0,12	2,12 ± 0,12	2,24 ± 0,10	2,32 ± 0,11	$P_{trước-sau} < 0,01$
% so với trước thử nghiệm		106,4 %	112,3 %	118,9 %	123,0 %	

**Thử 1 (T1)**	1,86 ± 0,07	2,06 ± 0,21	2,15 ± 0,17	2,27 ± 0,16	2,34 ± 0,17	$P_{trước\text{-}sau} < 0{,}001$<br>$P_{trước(T1\text{-}C)} > 0{,}05$<br>$P_{sau(T1\text{-}C)} > 0{,}05$
% so với trước thử nghiệm		111,0 %	115,7 %	122,2 %	126,2 %	
**Thử 2 (T2)**	1,98 ± 0,15	2,09 ± 0,21	2,23 ± 0,16	2,32 ± 0,17	2,40 ± 0,13	$P_{trước\text{-}sau} < 0{,}001$<br>$P_{trước(T2\text{-}C)} > 0{,}05$<br>$P_{sau(T2\text{-}C)} > 0{,}05$
% so với trước thử nghiệm		105,9 %	112,5 %	117,2 %	121,7 %	

Bảng 7. Theo dõi cân nặng thỏ sau 14 ngày ngừng uống mẫu thử

**Nhóm (*n* = 4)**	**Khối lượng cơ thể (kg)**	**$P_{sau\ 14\ ngày\ (T\text{-}C)}$**
Chứng (C)	2,45 ± 0,06	
Thử 1 (T1)	2,44 ± 0,12	> 0,05
Thử 2 (T2)	2,47 ± 0,18	> 0,05

2.3.2. Kết quả theo dõi các chỉ số huyết học liên quan tới chức năng tạo máu

<u>a. Trước thử nghiệm (trước khi uống mẫu thử)</u>

Bảng 8. Các chỉ số huyết học trước khi dùng mẫu thử

**Chỉ tiêu**	**Nhóm chứng (*n* = 7)**	**Nhóm T1 (*n* = 7)**	***P* *(T1-C)***	**Nhóm T2 (*n* = 7)**	***P* *(T2-C)***
**Hồng cầu** *(x 10^{12}/l)*	5,6 ± 0,3	5,3 ± 0,5	> 0,05	5,8 ± 0,5	> 0,05
**Bạch cầu** *(x 10^{9}/l)*	5,8 ± 1,2	7,4 ± 2,9	> 0,05	6,8 ± 1,2	> 0,05
**Tiểu cầu** *(x 10^{9}/l)*	383,1 ± 121,0	340,1 ± 87,7	> 0,05	415,1 ± 140,5	> 0,05
**Hematocrit** *(%)*	37,4 ± 2,1	35,2 ± 2,8	> 0,05	38,4 ± 2,4	> 0,05
**Hemoglobin** *(g/dl)*	11,2 ± 0,6	10,5 ± 1,1	> 0,05	11,5 ± 1,0	> 0,05

<u>b. Thời điểm sau 14 ngày uống mẫu thử</u>

Bảng 9. Các chỉ số huyết học sau 14 ngày dùng mẫu thử

Chỉ tiêu	Nhóm chứng (*n* = 7)	Nhóm T1 (*n* = 7)	*P* $_{(T1-C)}$	Nhóm T2 (*n* = 7)	*P* $_{(T2-C)}$
**Hồng cầu** (*x* $10^{12}/l$)	5,5 ± 0,4	5,2 ± 0,3	> 0,05	5,7 ± 0,2	> 0,05
**Bạch cầu** (*x* $10^{9}/l$)	6,6 ± 1,9	7,6 ± 1,7	> 0,05	7,6 ± 1,1	> 0,05
**Tiểu cầu** (*x* $10^{9}/l$)	329,4 ± 88,0	393,3 ± 45,7	> 0,05	338,0 ± 37,6	> 0,05
**Hematocrit** (*%*)	36,1 ± 2,5	34,7 ± 1,5	> 0,05	37,6 ± 1,7	> 0,05
**Hemoglobin** (*g/dl*)	11,3 ± 0,7	10,7 ± 0,7	> 0,05	11,6 ± 0,4	> 0,05

c. Thời điểm sau 28 ngày uống mẫu thử

Bảng 10. Các chỉ số huyết học sau 28 ngày dùng mẫu thử

Chỉ tiêu	Nhóm chứng (*n* = 7)	Nhóm T1 (*n* = 7)	*P* $_{(T1-C)}$	Nhóm T2 (*n* = 7)	*P* $_{(T2-C)}$
**Hồng cầu** (*x* $10^{12}/l$)	5,6 ± 0,3	5,4 ± 0,3	> 0,05	5,6 ± 0,2	> 0,05
**Bạch cầu** (*x* $10^{9}/l$)	8,1 ± 1,6	7,3 ± 1,0	> 0,05	8,6 ± 1,3	> 0,05
**Tiểu cầu** (*x* $10^{9}/l$)	356,3 ± 55,9	384,9 ± 69,9	> 0,05	348,7 ± 99,0	> 0,05
**Hematocrit** (*%*)	36,8 ± 1,7	35,9 ± 1,7	> 0,05	36,9 ± 1,0	> 0,05
**Hemoglobin** (*g/dl*)	11,6 ± 0,6	11,1 ± 0,8	> 0,05	11,5 ± 0,5	> 0,05

d. Thời điểm sau 14 ngày ngừng uống mẫu thử

Bảng 11. Các chỉ số huyết học sau 14 ngày ngừng uống mẫu thử

Chỉ tiêu	Nhóm chứng (*n* = 4)	Nhóm T1 (*n* = 4)	*P* $_{(T1-C)}$	Nhóm T2 (*n* = 4)	*P* $_{(T2-C)}$
**Hồng cầu** (*x* $10^{12}/l$)	5,7 ± 0,2	5,5 ± 0,4	> 0,05	5,6 ± 0,1	> 0,05

**Bạch cầu** *(x 10^9/l)*	9,7 ± 2,1	8,6 ± 1,0	> 0,05	9,1 ± 0,7	> 0,05
**Tiểu cầu** *(x 10^9/l)*	376,8 ± 76,1	406,5 ± 56,4	> 0,05	386,5 ± 58,1	> 0,05
**Hematocrit** *(%)*	37,5 ± 1,3	36,8 ± 2,4	> 0,05	36,4 ± 1,0	> 0,05
**Hemoglobin** *(g/dl)*	12,0 ± 0,4	11,8 ± 1,0	> 0,05	11,6 ± 0,5	> 0,05

Nhận xét

Kết quả xét nghiệm một số chỉ số huyết học cho thấy:

- Trước uống mẫu thử: Không có sự khác biệt có ý nghĩa về chỉ số huyết học giữa nhóm chứng và 2 nhóm thử ($P_{trước\ TN\ (T-C)}$ > ***0,05***).

- Sau 14 ngày, 28 ngày uống mẫu thử và sau 14 ngày ngừng uống mẫu thử: Không có sự khác biệt có ý nghĩa về chỉ số huyết học giữa nhóm chứng và 2 nhóm thử ($P_{sau\ 14ngày\ (T-C)}$> 0,05; $P_{sau\ 28\ ngày\ (T-C)}$ > 0,05), $P_{sau\ 14\ ngày\ ngừng\ uống\ mẫu\ thử(T-C)}$ > ***0,05).***

2.3.3. Kết quả theo dõi các chỉ số liên quan tới chức năng gan

a. Trước thử nghiệm (trước khi uống mẫu thử)

Bảng 12. Các chỉ số liên quan chức năng gan trước khi dùng mẫu thử

**Chỉ tiêu**	**Nhóm chứng (*n* = 7)**	**Nhóm T1 (*n* = 7)**	***P*** *(T1-C)*	**Nhóm T2 (*n* = 7)**	***P*** *(T2-C)*
**AST** *(U/l)*	44,0 ± 15,9	41,9 ± 18,8	> 0,05	39,4 ± 18,6	> 0,05
**ALT** *(U/l)*	53,0 ± 14,3	52,2 ± 14,8	> 0,05	49,6 ± 17,6	> 0,05
**Bilirubin toàn phần** *(µmol/l)*	1,9 ± 0,7	2,0 ± 0,4	> 0,05	1,9 ± 0,6	> 0,05
**Protein toàn phần** *(g/l)*	51,1 ± 3,1	53,0 ± 3,5	> 0,05	54,0 ± 5,8	> 0,05
**Albumin** *(g/dl)*	34,6 ± 1,6	34,6 ± 2,1	> 0,05	35,2 ± 4,8	> 0,05
**Cholesterol** *(mmol/l)*	2,4 ± 0,5	2,1 ± 0,6	> 0,05	2,7 ± 0,5	> 0,05

b. Thời điểm sau 14 ngày uống mẫu thử

Bảng 13. Các chỉ số liên quan chức năng gan sau 14 ngày dùng mẫu thử

Chỉ tiêu	Nhóm chứng (n = 7)	Nhóm T1 (n = 7)	$P_{(T1-C)}$	Nhóm T2 (n = 7)	$P_{(T2-C)}$
**AST** *(U/l)*	34,0 ± 16,5	33,6 ± 16,3	> 0,05	38,5 ± 12,6	> 0,05
**ALT** *(U/l)*	54,2± 17,0	55,9 ± 20,7	> 0,05	54,7 ± 19,4	> 0,05
**Bilirubin toàn phần** *(µmol/l)*	2,3 ± 0,3	2,3 ± 0,4	> 0,05	2,6 ± 0,5	> 0,05
**Protein toàn phần** *(g/l)*	49,5 ± 3,3	51,7 ± 4,9	> 0,05	51,6 ± 2,7	> 0,05
**Albumin** *(g/dl)*	34,2 ± 1,9	34,3 ± 1,5	> 0,05	34,6 ± 2,2	> 0,05
**Cholesterol** *(mmol/l)*	2,4 ± 0,6	2,1 ± 0,8	> 0,05	2,3 ± 1,0	> 0,05

c. Thời điểm sau 28 ngày uống mẫu thử

Bảng 14. Các chỉ số liên quan chức năng gan sau 28 ngày dùng mẫu thử

Chỉ tiêu	Nhóm chứng (n = 7)	Nhóm T1 (n = 7)	$P_{(T1-C)}$	Nhóm T2 (n = 7)	$P_{(T2-C)}$
**AST** *(U/l)*	35,6 ± 14,6	33,8 ± 10,4	> 0,05	34,4 ± 10,3	> 0,05
**ALT** *(U/l)*	58,6 ± 21,7	52,8 ± 15,3	> 0,05	52,6 ± 20,5	> 0,05
Bilirubin **toàn phần** *(µmol/l)*	2,6 ± 0,3	2,6 ± 0,3	> 0,05	2,7 ± 0,3	> 0,05
**Protein toàn phần** *(g/l)*	51,2 ± 1,7	51,1 ± 4,8	> 0,05	52,1 ± 2,0	> 0,05
**Albumin** *(g/dl)*	35,4 ± 1,9	35,8 ± 1,7	> 0,05	35,8 ± 0,9	> 0,05
**Cholesterol** *(mmol/l)*	2,6 ± 1,0	1,9 ± 0,5	> 0,05	1,8 ± 0,5	> 0,05

d. Thời điểm sau 14 ngày ngừng uống mẫu thử

Bảng 15. Các chỉ số liên quan chức năng gan sau 14 ngày ngừng uống mẫu thử

Chỉ tiêu	Nhóm chứng (n = 4)	Nhóm T1 (n = 4)	$P_{(T1-C)}$	Nhóm T2 (n = 4)	$P_{(T2-C)}$
**AST** *(U/l)*	36,9 ± 6,2	32,8 ± 9,0	> 0,05	42,0 ± 15,2	> 0,05
**ALT** *(U/l)*	62,5 ± 16,7	58,0 ± 19,0	> 0,05	58,1 ± 16,7	> 0,05
**Bilirubin toàn phần** *(µmol/l)*	2,5 ± 0,2	2,3 ± 0,2	> 0,05	2,5 ± 0,3	> 0,05
**Protein toàn phần** *(g/l)*	54,6 ± 3,7	52,2 ± 3,6	> 0,05	55,2 ± 3,1	> 0,05
**Albumin** *(g/dl)*	35,8 ± 1,7	36,8 ± 2,0	> 0,05	36,5 ± 0,9	> 0,05
**Cholesterol** *(mmol/l)*	2,1 ± 0,7	1,6 ± 0,2	> 0,05	1,6 ± 0,5	> 0,05

Nhận xét

Kết quả xét nghiệm một số chỉ số chức năng gan cho thấy:

- Trước uống mẫu thử: Không có sự khác biệt có ý nghĩa về các chỉ số liên quan đến chức năng gan giữa nhóm chứng và 2 nhóm thử ($P_{trướcTN\ (T-C)} > 0,05$).

- Sau 14 ngày uống mẫu thử, sau 28 ngày uống mẫu thử và sau 14 ngày ngừng uống mẫu thử: Không có sự khác biệt có ý nghĩa về các chỉ số liên quan đến chức năng gan giữa nhóm chứng và 2 nhóm thử ($P_{sau\ 14\ ngày(T-C)} > 0,05$; $P_{sau\ 28\ ngày(T-C)} > 0,05$), $P_{sau\ 14\ ngày\ ngừng\ uống\ mẫu\ thử(T-C)} > 0,05$).

2.3.4. Kết quả theo dõi các chỉ số liên quan tới chức năng thận

a. Trước thử nghiệm (trước khi uống mẫu thử)

Bảng 16. Các chỉ số liên quan chức năng thận trước khi dùng mẫu thử

Chỉ tiêu	Nhóm chứng (n = 7)	Nhóm T1 (n = 7)	$P_{(T1-C)}$	Nhóm T2 (n = 7)	$P_{(T2-C)}$
**Urea** *(mmol/l)*	4,0 ± 1,0	4,4 ± 0,5	> 0,05	4,6 ± 0,8	> 0,05

**Creatinin** *(µmol/l)*	76,3 ± 9,1	79,8 ± 10,4	> 0,05	82,1 ± 15,2	> 0,05

b. Thời điểm sau 14 ngày uống mẫu thử

Bảng 17. Các chỉ số liên quan chức năng thận sau 14 ngày dùng mẫu thử

Chỉ tiêu	Nhóm chứng (*n* = 7)	Nhóm T1 (*n* = 7)	*P* *(T1-C)*	Nhóm T2 (*n* = 7)	*P* *(T2-C)*
**Urea** *(mmol/l)*	4,3 ± 0,5	3,8 ± 1,0	> 0,05	4,7 ± 1,1	> 0,05
**Creatinin** *(µmol/l)*	92,3 ± 17,7	95,9 ± 16,3	> 0,05	100,0 ± 12,0	> 0,05

c. Thời điểm sau 28 ngày uống mẫu thử

Bảng 18. Các chỉ số liên quan chức năng thận sau 28 ngày dùng mẫu thử

Chỉ tiêu	Nhóm chứng (*n* = 7)	Nhóm T1 (*n* = 7)	*P* *(T1-C)*	Nhóm T2 (*n* = 7)	*P* *(T2-C)*
**Urea** *(mmol/l)*	5,3 ± 0,9	4,6 ± 0,7	> 0,05	4,7 ± 0,5	> 0,05
**Creatinin** *(µmol/l)*	94,8 ± 14,8	93,2 ± 13,4	> 0,05	94,1 ± 14,7	> 0,05

d. Thời điểm sau 14 ngày ngừng uống mẫu thử

Bảng 19. Các chỉ số liên quan chức năng thận sau 14 ngày ngừng uống mẫu thử

Chỉ tiêu	Nhóm chứng (*n* = 4)	Nhóm T1 (*n* = 4)	*P* *(T1-C)*	Nhóm T2 (*n* = 4)	*P* *(T2-C)*
**Urea** *(mmol/l)*	5,5 ± 0,4	4,9 ± 0,4	> 0,05	5,1 ± 1,1	> 0,05
**Creatinin** *(µmol/l)*	99,3 ± 6,7	93,0 ± 7,6	> 0,05	98,5 ± 17,8	> 0,05

Nhận xét

Kết quả xét nghiệm urea và creatinin cho thấy:

- Trước uống mẫu thử: Không có sự khác biệt có ý nghĩa về các chỉ số liên quan đến chức năng thận giữa nhóm chứng và 2 nhóm thử (**$P_{trướcTN\ (T-C)}$ > 0,05**).

- Sau 14 ngày uống mẫu thử, sau 28 ngày uống mẫu thử và sau 14 ngày ngừng uống mẫu thử: Không có sự khác biệt có ý nghĩa về các chỉ số liên quan đến chức năng thận giữa nhóm chứng và 2 nhóm thử ($P_{sau\ 14\ ngày(T-C)}$> 0,05; $P_{sau\ 28\ ngày(T-C)}$ > 0,05), $P_{sau\ 14\ ngày\ ngừng\ uống\ mẫu\ thử(T-C)}$ > 0,05).

2.3.5. Kết quả theo dõi chỉ số glucose trong huyết tương

a. Trước thử nghiệm (trước khi uống mẫu thử)

Bảng 20. Chỉ số glucose trước khi dùng mẫu thử

**Chỉ tiêu**	**Nhóm chứng (*n* = 7)**	**Nhóm T1 (*n* = 7)**	***P** (T1-C)*	**Nhóm T2 (*n* = 7)**	***P** (T2-C)*
**Glucose** *(mmol/l)*	6,7 ± 1,2	6,6 ± 0,6	> 0,05	6,1 ± 1,1	> 0,05

b. Thời điểm sau 14 ngày uống mẫu thử

Bảng 21. Chỉ số glucose sau 14 ngày dùng mẫu thử

**Chỉ tiêu**	**Nhóm chứng (*n* = 7)**	**Nhóm T1 (*n* = 7)**	***P** (T1-C)*	**Nhóm T2 (*n* = 7)**	***P** (T2-C)*
**Glucose** *(mmol/l)*	5,9 ± 1,0	5,3 ± 0,9	> 0,05	5,4 ± 1,4	> 0,05

c. Thời điểm sau 28 ngày uống mẫu thử

Bảng 22. Chỉ số glucose sau 28 ngày dùng mẫu thử

**Chỉ tiêu**	**Nhóm chứng (*n* = 7)**	**Nhóm T1 (*n* = 7)**	***P** (T1-C)*	**Nhóm T2 (*n* = 7)**	***P** (T2-C)*
**Glucose** *(mmol/l)*	4,4 ± 1,1	4,1 ± 0,7	> 0,05	4,5 ± 0,8	> 0,05

d. Thời điểm sau 14 ngày ngừng uống mẫu thử

Bảng 23. Chỉ số glucose sau 14 ngày ngừng uống mẫu thử

Chỉ tiêu	Nhóm chứng (n = 4)	Nhóm T1 (n = 4)	$P_{(T1-C)}$	Nhóm T2 (n = 4)	$P_{(T2-C)}$
**Glucose** *(mmol/l)*	4,9 ± 0,3	5,5 ± 0,7	> 0,05	4,9 ± 0,9	> 0,05

Nhận xét

Kết quả xét nghiệm glucose cho thấy:

- Trước uống mẫu thử: Không có sự khác biệt có ý nghĩa về chỉ số glucose giữa nhóm chứng và 2 nhóm thử (***$P_{trướcTN\ (T-C)}$ > 0,05***).

- Sau 14 ngày uống mẫu thử, sau 28 ngày uống mẫu thử và sau 14 ngày ngừng uống mẫu thử: Không có sự khác biệt có ý nghĩa về các chỉ số glucose giữa nhóm chứng và 2 nhóm thử ($P_{sau\ 14\ ngày(T-C)}$ > 0,05; $P_{sau\ 28\ ngày(T-C)}$> 0,05), $P_{sau\ 14\ ngày\ ngừng\ uống\ mẫu\ thử(T-C)}$ > 0,05).

2.3.6. Quan sát đại thể

Kết quả quan sát đại thể các cơ quan nội tạng của tất cả các thỏ thử nghiệm cho thấy: Không có biểu hiện khác thường về hình dạng bên ngoài, màu sắc của các tổ chức tim, phổi, gan, lách, thận, dạ dày, ruột của các thỏ nhóm thử so với nhóm chứng sau thử nghiệm.

Bảng 24. Hình ảnh đại thể các cơ quan nội tạng

**Hình ảnh đại thể**		
Nhóm Chứng	***Nhóm Thử 1 (liều thấp)***	***Nhóm Thử 2 (liều cao)***
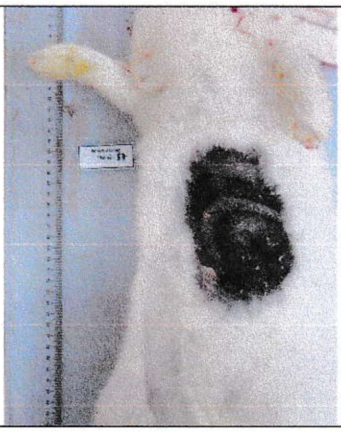	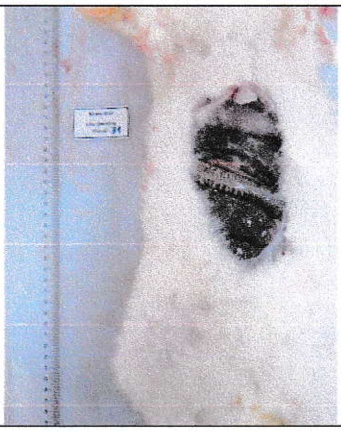	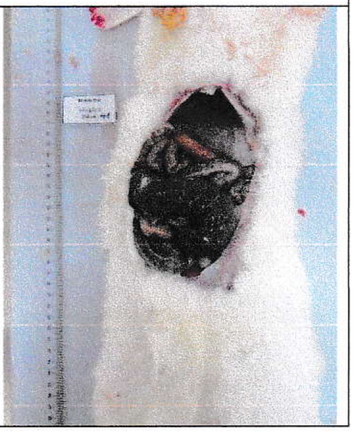

2.3.7. Quan sát vi thể

Tiêu bản gan, thận, ruột non, đại tràng được cố định bằng Formalin 10 %, nhuộm bằng dung dịch nhuộm Hematoxylin Eosin (HE) và Perioric Acid Shiff (PAS) và quan sát dưới kính hiển vi quang học.

Kết quả quan sát vi thể do Bộ môn Giải phẫu sinh lý bệnh - Trường Đại học Y Hà Nội thực hiện cho thấy: Các thỏ thử nghiệm đều có gan, thận, ruột non, đại tràng không bị tổn thương, hình ảnh cấu trúc trong giới hạn bình thường. Không có các triệu chứng bất thường liên quan đến mẫu thử với 2 mức liều khác nhau so với nhóm chứng.

Cụ thể như sau:

Gan:

- Tế bào gan: Không thấy tổn thương.
- Mao mạch nan hoa: Bình thường.
- Tĩnh mạch trung tâm: Bình thường.
- Khoảng cửa: Bình thường.
- Mô kẽ: Bình thường.

Kết luận: Mô gan bình thường, không thấy tổn thương.

Thận:

- Tiểu cầu thận (mao mạch, gian mạch, bao Baomann): Bình thường.
- Ống thận: Bình thường.
- Đài bể thận: Bình thường.
- Mô kẽ: Bình thường.

Kết luận: Mô thận bình thường, không thấy tổn thương.

Ruột non:

- Niêm mạc (nhung mao, tế bào hấp thu, tế bào hình đài, mô đệm, cơ niêm): Bình thường.
- Dưới niêm mạc: Bình thường.
- Lớp cơ: Bình thường.
- Thanh mạc: Bình thường.

Kết luận: Mô ruột non bình thường, không thấy tổn thương.

Đại tràng:

- Niêm mạc (biểu mô trụ đơn, tế bào hình đài tiết nhầy, tuyến Lieberkuhn, mô đệm, cơ niêm): Bình thường.
- Dưới niêm mạc: Bình thường.
- Lớp cơ: Bình thường.
- Thanh mạc: Bình thường.

Kết luận: Mô đại tràng bình thường, không thấy tổn thương.

Bảng 25. Hình ảnh giải phẫu mô bệnh học gan, thận, ruột non, đại tràng (Hình ảnh nhuộm HE, Độ phóng đại 400 lần)

STT	*Nhóm*	*Hình ảnh mô gan*	*Hình ảnh mô thận*	*Hình ảnh mô ruột non*	*Hình ảnh mô đại tràng*
1	*Chứng*	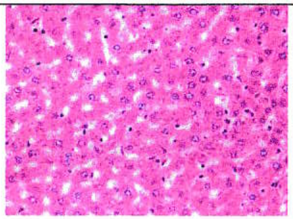<br>Mô gan bình thường, không thấy tổn thương.	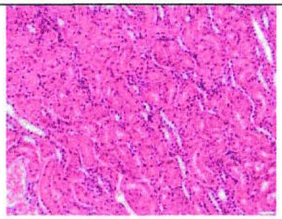<br>Mô thận bình thường, không thấy tổn thương.	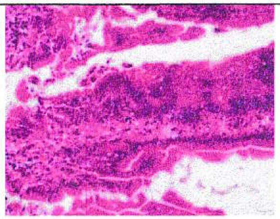<br>Mô ruột non bình thường, không thấy tổn thương.	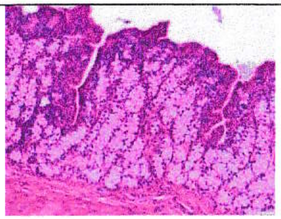<br>Mô đại tràng bình thường, không thấy tổn thương.
2	*Thử 1 (liều thấp)*	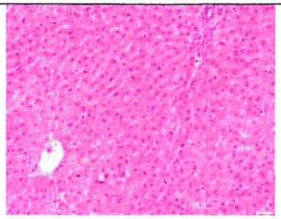<br>Mô gan bình thường, không thấy tổn thương.	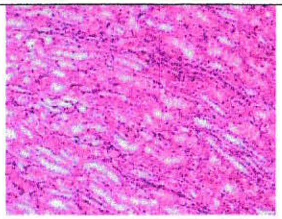<br>Mô thận bình thường, không thấy tổn thương.	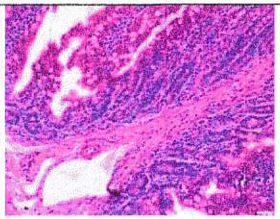<br>Mô ruột non bình thường, không thấy tổn thương.	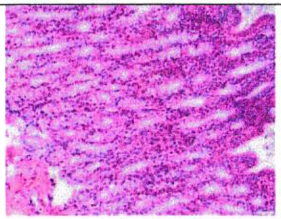<br>Mô đại tràng bình thường, không thấy tổn thương.
3	*Thử 2 (liều cao)*	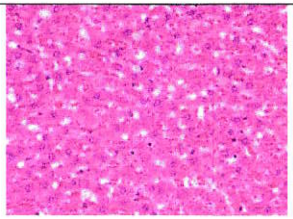<br>Mô gan bình thường, không thấy tổn thương.	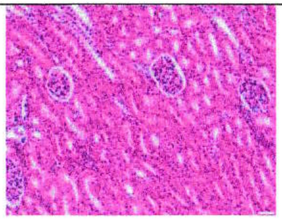<br>Mô thận bình thường, không thấy tổn thương.	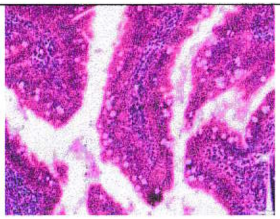<br>Mô ruột non bình thường, không thấy tổn thương.	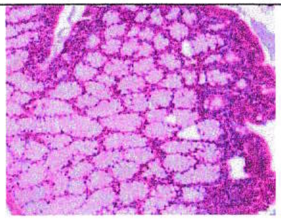<br>Mô đại tràng bình thường, không thấy tổn thương.

Bảng 26. Hình ảnh giải phẫu mô bệnh học gan, thận, ruột non, đại tràng (Hình ảnh nhuộm PAS, Độ phóng đại 400 lần)

STT	*Nhóm*	*Hình ảnh mô gan*	*Hình ảnh mô thận*	*Hình ảnh mô ruột non*	*Hình ảnh mô đại tràng*
1	***Chứng***	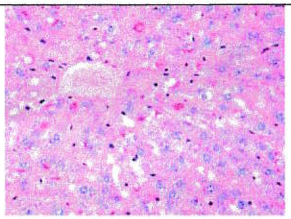 Mô gan bình thường, không thấy tổn thương.	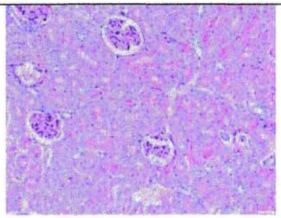 Mô thận bình thường, không thấy tổn thương.	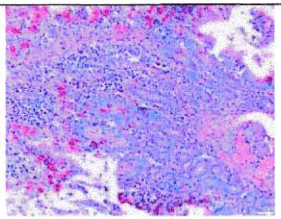 Mô ruột non bình thường, không thấy tổn thương.	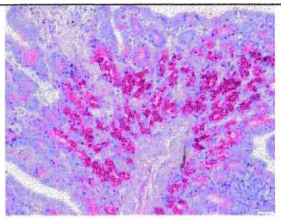 Mô đại tràng bình thường, không thấy tổn thương.
2	***Thử 1 (liều thấp)***	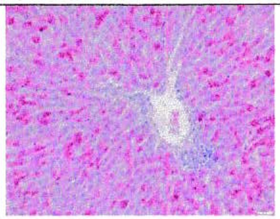 Mô gan bình thường, không thấy tổn thương.	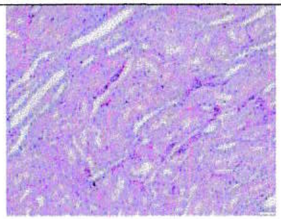 Mô thận bình thường, không thấy tổn thương.	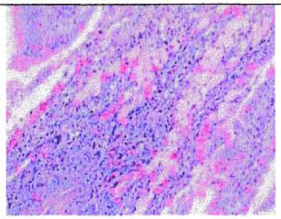 Mô ruột non bình thường, không thấy tổn thương.	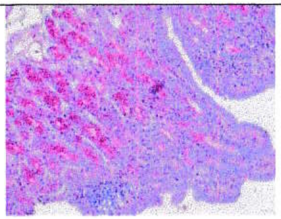 Mô đại tràng bình thường, không thấy tổn thương.
3	***Thử 2 (liều cao)***	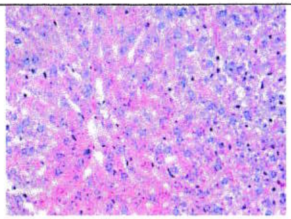 Mô gan bình thường, không thấy tổn thương.	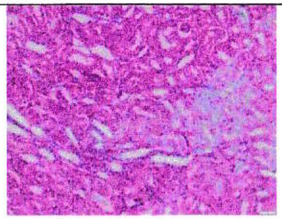 Mô thận bình thường, không thấy tổn thương.	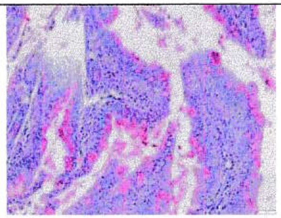 Mô ruột non bình thường, không thấy tổn thương.	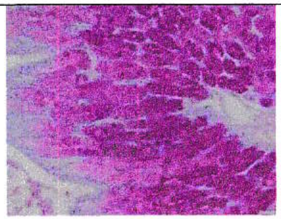 Mô đại tràng bình thường, không thấy tổn thương.

2.4. Kết luận

Mẫu thử **Nguyên liệu 3 – Bacillus Mixture cho sản phẩm LiveSpo Preg-Mom và LiveSpo Dia30** gửi tới yêu cầu thử độc tính bán trường diễn trên thỏ có kết quả như sau:

Sau khi cho thỏ uống hỗn dịch mẫu thử liên tục trong 28 ngày với 2 mức liều khác nhau là 0,93 mL (4,65 tỷ CFU)/kg thỏ/ngày (tương ứng với mức liều tối đa dùng cho người là 3 ống/người/ngày hay tương đương với 75 tỷ CFU *Bacillus subtilis, Bacillus clausii* và *Bacillus coagulans*/người/ngày) và 4,65 mL (23,25 tỷ CFU)/kg thỏ/ngày (cao gấp 5 lần so với liều dùng tối đa ngoại suy từ liều dùng của người, tương đương với tương đương với 375 tỷ CFU *Bacillus subtilis, Bacillus clausii* và *Bacillus coagulans*/người/ngày), mẫu thử không gây ảnh hưởng đến cân nặng, thể trạng, vận động của thỏ thí nghiệm. Thỏ khỏe mạnh, tăng cân.

Về các chỉ số sinh hóa đánh giá chức năng gan, thận (hoạt độ các enzyme AST, ALT, protein toàn phần, bilirubin toàn phần, cholesterol, albumin, glucose, urea, creatinin) và các chỉ số huyết học (hồng cầu, hemoglobin, hematocrit, bạch cầu, tiểu cầu) không có sự khác biệt có ý nghĩa ở trước thử nghiệm, sau 14 ngày uống mẫu thử, sau 28 ngày uống mẫu thử, và sau 14 ngày ngừng uống mẫu thử giữa hai nhóm thử nghiệm so với nhóm chứng.

Không nhận thấy bất thường ở các tổ chức tim, phổi, gan, lách, thận, dạ dày, ruột của thỏ thí nghiệm khi quan sát đại thể cũng như không nhận thấy tổn thương mô bệnh học của gan, thận, ruột non, đại tràng khi quan sát vi thể giữa hai nhóm thử và nhóm chứng.

Hà Nội, ngày 19 tháng 7.. năm 2023

Xác nhận của cơ quan

VIỆN TRƯỞNG

BỘ Y TẾ VIỆN KIỂM NGHIỆM THUỐC TRUNG ƯƠNG

Đoàn Cao Sơn

KHOA DƯỢC LÝ

TS. Nguyễn Thị Liên
